# ﻿*Papaver* recircumscribed: A review of neighbouring Papaveraceae genera, including *Afropapaver* nom. et stat. nov. and *Oreomecon*, a large, Arctic-Alpine genus

**DOI:** 10.3897/phytokeys.248.121011

**Published:** 2024-10-29

**Authors:** Arve Elvebakk, Jarle W. Bjerke

**Affiliations:** 1 Arctic University Museum of Norway, UiT The Arctic University, PO Box 6050 Langnes, NO-9037 Tromsø, Norway UiT The Arctic University Tromsø Norway; 2 Norwegian Institute for Nature Research, FRAM – High North Research Centre for Climate and the Environment, PO Box 6606 Langnes, NO-9296 Tromsø, Norway Norwegian Institute for Nature Research Tromsø Norway

**Keywords:** Distribution, *Meconopsis*, *Parameconopsis*, phylogeny, poppies, species diversity, *Stylomecon*, taxonomy

## Abstract

Papaveraceae tribus Papavereae includes an American and a mainly Eurasian group of genera. The latter is proposed here to include eight genera. Amongst these, the recently described genus *Oreomecon* is phylogenetically a sister group to *Meconopsis*, a genus from Himalaya and central China, which is reviewed here as including 95 species and 21 subspecies. By contrast, *Oreomecon* has a circumpolar northern alpine and Arctic distribution, including incompletely understood taxa, many threatened by climatic warming. Based on a review of literature and phylogenies, it is proposed here that *Oreomecon* includes 68 species and 29 subspecies. *Oreomeconaurantiaca*, *O.cornwallisensis*, *O.keelei*, *O.ochotensis* and *O.uschakovii*, 29 subspecies and four varieties are placed in *Oreomecon* here, 29 of these as recombinations, the remaining ones as nomenclatural novelties. A total of 21 existing *Oreomecon* names are placed into synonymy. The taxonomically challenging *O.alpina* group from Central Europe is treated as comprising three species, with the remaining entities positioned at the subspecies level pending further studies. The much-studied Nordic species *O.radicata* is treated with eight subspecies here, based on morphometric studies, whereas four accepted entities are provisionally recombined at the variety level. The name *Papavertenellum* and the basionyms of *Oreomeconalborosea*, O.alpinasubsp.corona-sancti-stephani, O.alpinasubsp.degenii, *O.anomala*, O.lapeyrouseanasubsp.endressii, O.lapponicasubsp.laestadiana and *O.nivalis* are lectotypified here. Two replacement names, Oreomeconalpinasubsp.markgrafiana and O.radicatasubsp.knabeniana, are introduced.

*Papaver*, as currently understood, is recircumscribed here to represent four genera. The isolated sectionHorrida, from southern Africa, is raised to genus level with the new name *Afropapaver* and its only species is recombined as *Afropapaveraculeatum*. Papaversect.Californica from California and adjacent Mexico is treated as the genus *Stylomecon*. The name has been applied to one of the two species of this group and we now recombine the other one as *S.crassifolia*, based on an older basionym replacing *Papavercalifornicum*. *Papavercambricum* is accepted in its alternative position as the monotypic genus *Parameconopsis*. As reviewed here, *Papaver* comprises 59 species and 14 subspecies and is only the third-largest genus in the group. Based on the distribution of its closest relatives and oldest sections, it is hypothesised here that *Papaver* arose in the western Mediterranean. Its poricidal capsule dehiscence serves as an excellent adaptation to seed dispersal in open, arid environments, possibly explaining its later success in the Türkiye-Caucasus-Middle East area, where its diversity both at species and section level is highest.

## ﻿Introduction

*Papaver* L. and *Meconopsis* Vig. are two large plant genera with striking flowers and both are very popular in gardens, the latter restricted to areas with cool climates (Grey-Wilson 2014, 2017; Stevens 2015). In addition, *Papaver* is also very important in medicinal research (Sariyar 2002; Catania et al. 2022), while *Meconopsis* has been extensively used in regional ethno-medicine (Guo et al. 2016). These genera belong to the tribus Papavereae Dumontier of the subfamily Papaveroideae Eaton of the large family Papaveraceae with its long evolutionary history as shown by its estimated crown age of 120 Ma (Stevens 2001 onwards; Peng et al. 2023). Papavereae, with its embedded tribe Platystemoneae Spach, was split into a North American and a Eurasian clade in sister group positions after a dispersal event from Asia to North America at 81.5 Ma (Peng et al. 2023).

*Papaver* and *Meconopsis* are the largest genera within the latter group. However, the differentiation between these genera has been phylogenetically problematic. Consequently, Kadereit et al. (2011) proposed five actions to obtain monophyly. The first required action was a reclassification of *Meconopsiscambrica* (L.) Vig. to its former basionym *Papavercambricum* L. accompanied by a necessary lectotypification, the latter made by Ferrer-Gallego (2015). The second was defining a new generitype for *Meconopsis* replacing *M.cambrica* after the recircumscription of the genus, which was done by Grey-Wilson (2012).

The long evolutionary history of *Papavercambricum* shown by Valtueña et al. (2012), combined with its distinct morphology, calls for a status as a separate genus. Thus, the new genus *Parameconopsis* Grey-Wilson was proposed (Grey-Wilson 2014), albeit without any thorough phylogenetic discussion. Valtueña et al. (2012) showed that *Papavercambricum* represents a phylogenetic sister group to most sections of the remaining parts of *Papaver*, except for two clades with a strongly deviating distribution. One clade consists of the southern African species *P.aculeatum* Thunb. and the second clade comprises the species pair *P.californicum* A.Gray and *P.heterophyllum* (Benth.) Greene from California and northwesternmost Mexico. Some authors have positioned the latter species within the genus *Stylomecon* G.Taylor.

A narrow concept of *Papaver* s.str. had been argued for by Kadereit et al. (1997:93, 2011:83), but Kadereit and Baldwin (2011) compared the alternatives of maintaining several lineages within *Papaver* s.lat. vs. their segregation as new genera. In the latter alternative, the lineage with *Stylomecon* had an existing name alternative. However, three of the other lineages did not and Kadereit and Baldwin (2011) favoured a more widely defined interpretation of the genus *Papaver*, which avoided splitting and nomenclatural changes. Their arguments also stated that the deviating stylar capsules of *Papavercambricum* and *P.heterophyllum* had evolved independently.

The third action proposed by Kadereit et al. (2011) to obtain monophyly within Papavereae was to define *Meconopsis* species from basal parts of phylograms as the genus *Cathcartia* Hook.f. This proposal was strongly supported by later phylogenies (Liu et al. 2014; Xie et al. 2014; Xiao and Simpson 2015), which focused on *Meconopsis*, but included other groups. The additional recombinations needed to complete the recircumscription of *Cathcartia* were made in the extensive *Meconopsis* monograph by Grey-Wilson (2014). The fourth proposal by Kadereit et al. (2011) was to expand *Roemeria* Medik. by also transferring species of Papaversect.Argemonidium Spach (Kadereit 1986a) into *Roemeria*. Based on the most recent revision of this section by Aghababyan (2011a), Banfi et al. (2022) completed this process by transferring nine species to *Roemeria*.

Another phylogenetically challenging clade within *Papaver* s.lat. is P.sect.Meconella Spach. Its species are perennial, scapose, mostly with bristly capsules, with deep incisions between the stigmatic rays and white or yellow to orange and pink flowers (Carolan et al. 2006). In her monograph of the section, Rändel (1974) accepted 24 species and 15 additional subspecies and presented a distribution map and a map of ploidy levels indicating possible migration routes. As an East German researcher, she profitted from close contact with botanists in the former Soviet Union, where most of the species of this group had been described. She later added three additional species and two subspecies from North America (Rändel 1977). The section included 30 species, according to Carolan et al. (2006), whereas Solstad et al. (2009), in a thesis, monographed the group, included a determination key and accepted 54 species and 15 subspecies, based on taxonomy concepts which are largely identical in the Pan-Arctic Flora review by Elven et al. (2011).

Phylogenetic analyses now clearly identify this section as a sister clade to *Meconopsis* and not as a subgroup within *Papaver* (Carolan et al. 2006; Kadereit et al. 2011; Liu et al. 2014; Xie et al. 2014). Consequently, the fifth recommendation by Kadereit et al. (2011) was that this section should be described as a new separate genus, with a new name as the genus name *Meconella* Nutt. is already in use for three unrelated species from North America (Hannan 1997).

Carolan et al. (2006) also discussed two other alternatives for obtaining monophyly within this part of Papaveraceae: PapaverSect.Meconella could be merged into *Meconopsis* or *Meconopsis* could be merged into *Papaver*. Christenhusz et al. (2018) argued for the latter alternative, proposing it as an operating taxonomy by providing numerous new recombinations of *Meconopsis* names to be positioned within *Papaver*, also including some recombinations of *Cathcartia* and *Roemeria* names.

To our knowledge, the latter classification alternative has not been adopted by any other major study or database. However, until recently, this was still the only alternative showing monophyly within a significant part of the presently polyphyletic classification of *Papaver*. Then, Banfi et al. (2022) described the new genus *Oreomecon* Banfi et al., with Papaversect.Meconella as basionym. A total of six species and one subspecies were recombined into the new genus. This included the well-known species *Papaveralpinum* L., which they interpreted in a broad sense following the classification by Schönswetter et al. (2009). The remaining five recombined species were referred to as being “Arctic”, but their distributions are not within the Arctic as defined by Walker et al. (2005).

Recently, Galasso et al. (2023) transferred another 12 species and one subspecies to *Oreomecon* with the intention “to provide names for all the taxa now included in *Oreomecon*”. In a coordinated paper, Grey-Wilson (2023) recombined another two species and one subspecies into this genus. Altogether, these additions cover only a small number of the taxa previously included in Papaversect.Meconella, with Krivenko (2023) recombining another 61 taxa into *Oreomecon*. This apparently finalised the replacement of Papaversect.Meconella names into the new genus, now totalling 81 species and three subspecies, but Krivenko (2023) did not accept any taxa at the subspecific level. He instead raised several previous names at variety and subspecies level to the rank of species without any discussions related to their original descriptions, whereas Xue et al. (2020) argued against using such a narrow species concept in treatments on a global scale. The only *Oreomecon* taxon which has been thoroughly dealt with is the subspecies treated by Banfi et al. (2022), a taxon discussed further and raised to the species level recently by Ferrer-Gallego (2024).

In our opinion, a critical review of studies dealing with Papaversect.Meconella is needed as a basis for an evaluation of which taxa to accept and treat within *Oreomecon*. This will be done for separate geographical areas below. A state-of-the-art phylogram of existing ITS sequences will also be presented, even if ITS has not, so far, been found to be a handy phylogenetic marker for this group (Carolan et al. 2006; Solstad et al. 2009). Solstad et al. (2009) also included an extensive genetic AFLP analysis and concluded that this method is useful in comparing related taxa and populations, like in the amphi-Atlantic area, which was most densely sampled. However, it does not reflect the group’s evolutionary history on a broader scope.

Examples of threatened species from all parts of the distribution area of *Oreomecon* are also presented. This is because their High Arctic and high alpine habitats are warming faster than most other biomes in the world and these species are, therefore, threatened by faster-growing forbs and woody species (Myers-Smith et al. 2020).

The present study aims to present a revised and monophyletic, generic classification of the Eurasian Papavereae group and to include an updated survey of accepted taxa within each genus. Based on existing phylogenies, evolutionary old and distinct lineages with morphological characteristics and distribution patterns are proposed as separate genera. A review of the literature on the genera and sections in the case of *Meconopsis* and *Papaver* is provided. For each of these groups, a key reference is provided for more extensive information. The global distributions of all genera are mapped with indications of their total numbers of accepted species.

## ﻿Material and methods

The present study relies primarily on critical surveys of taxonomic and phylogenetic literature, although supported by experiences from field studies and comparative cultivation of ca. 60 species of this group in Tromsø Arctic-Alpine Botanic Garden in Tromsø, northern Norway. The plants were grown in mineral-dominated soil in rock landscapes exposed to the local climate at almost 70°N latitude and cited specimens have been in cultivation for several years.

DNA sequences were retrieved from GenBank (National Center for Biotechnology Information (NCBI), USA) via the software Geneious Prime (ver. 2023.0.1, Biomatters, Auckland, New Zealand). The same software was used for tree alignment. All sequences available for species belonging to the genus *Oreomecon* (see Results section for species) were downloaded and checked for origins and sizes. Most sequences were from published sources, while a few sequences had not previously been used in published trees. Phylogeny was inferred from ITS-1, 5.8 S rRNA and ITS-2 sequences. Genetic distance was calculated using the Tamura-Nei genetic distance model (Tamura and Nei 1993).

For this study, species delineation within each treated genus or a major section of a genus relies on core monograph studies, as defined in Table 1. These are referred to for supplementary information, including lists of synonyms. In cases where our taxonomic apprehension deviates from or supplements these reference studies, the rationale behind our revisions is provided. The general delimitation of the Arctic follows Walker et al. (2005).

**Table 1.** List of reference studies on genera and subgroups of genera referred to for further information. Number of species and subspecies (in parentheses) of each group and subgroup are indicated following the supplementary revisions and additions provided in the studies referred to in the running text.

Genus	Subgroup	References	Species no.
*Cathcartia*		Grey-Wilson (2014)	4
*Roemeria*		Hassler (2023b)	16 (+1)
*Oreomecon*	Non-Arctic Siberia and Central Asia	Peschkova (1994)	24
	The Asian Far East	Bezdeleva (1987), Bezdeleva et al. (2006)	9 (+1)
	Arctic Asia	Tolmachev (1975), Petrovsky (1983, 1985)	14(+6)
	Arctic Alaska and Yukon and adjacent Cordilleras	Elven et al. (2011)	8 (+1)
	The major part of the North American Cordilleras	Björk (2019)	5
	Central and Eastern Canada, Greenland, Arctic Europe	Elven et al. (2011)	4 (+2)
	Non-Arctic northern Europe	Nilsson (2001)	1 (+8)
	Central Europe	Schönswetter et al. (2009)	3 (+11)
	Total		68 (+29)
*Meconopsis*		Grey-Wilson (2014)	95 (+21)
*Afropapaver*		Kadereit (1988b)	1
*Stylomecon*		Kadereit and Baldwin (2011)	2
*Papaver*	sect.Papaver	Kadereit (1986b)	4
	sect.Carinatae	Kadereit (1987)	1
	sect.Macrantha	Lack (2019a; b)	3
	sect.Rhoeadium	Kadereit (1989)	34 (+3)
	sect.Meconidium	Kadereit (1993)	7 (+5)
	sect.Pilosa	Kadereit (1996)	1
	sect.Pseudopilosa	Kadereit (1996)	8 (+2)
	Total		59 (+10)
*Parameconopsis*		Grey-Wilson (2014)	1
**Grand Total**			**246 (+61)**

POWO (2023) and “World Plants” (Hassler 2023b) were used extensively in search of names and interpretations, although their opinions were not automatically accepted. Nevertheless, they remain a significant source of synonyms and publication information. “The International Plant Names Index” (IPNI 2023) has also been used, as has the GBIF (Global Biodiversity Information Facility) Secretariat (2023). JSTOR Global Plants (2023) was also used as a supplementary source for information on type specimens. The cited sources above are referred to for information on heterotypic synonyms of *Oreomecon* names, in addition to those referred to in the text. Selected texts in Russian were interpreted using Google Translator.

Many of the names newly introduced here have basionyms that previously have been included in *Oreomecon* or previously treated at the same rank. They are, therefore, neither “stat. nov.” nor “comb. nov.” and are here referred to as nomenclatural novelties (“nomencl. nov.”), following the Code (Art. 6.10; Turland et al. (2018)).

**Figure 1.**
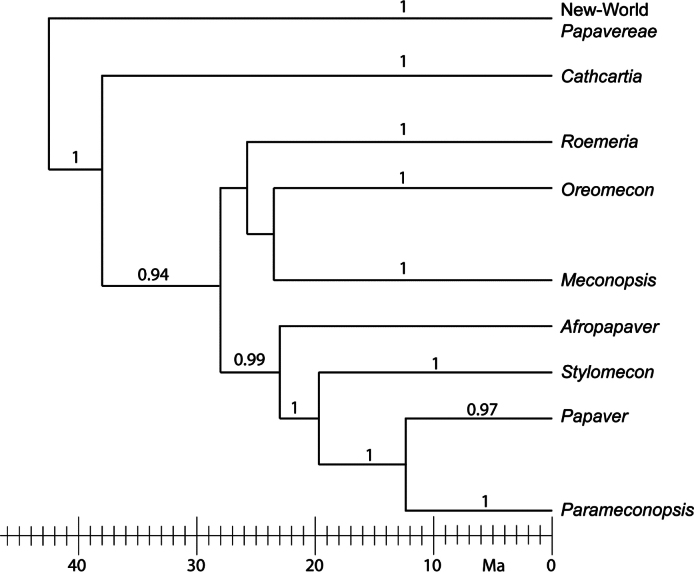
A summary version of the phylogram with chronology from Kadereit et al. (2011) with the names of the clades adopted in the present study.

The eight genera treated here are dealt with according to their sequences shown in Fig. 1. The review of Papaveris organised by thesectionconceptintroducedbyKiger (1985) and later adapted by Kadereit (1988a). *Meconopsis* is also reviewed section-wise, following Grey-Wilson (2014), although sections with no or minor later changes have been lumped. In the case of *Oreomecon*, all accepted species and subspecies names are listed and they are organised by their distributions within eight geographic areas. As this northern genus is particularly vulnerable to climatic change effects, literature sources summarising endangered populations and Red List statuses are also included. The treatments below do not include hybrids, varieties and named forms, except for taxa within the much-studied Nordic species *Oreomeconradicata* (Rottb.) Banfi et al.

## ﻿Results

A simplified summary of the ITS-based phylogram by Kadereit et al. (2011) is shown here as Fig. 1 with our name conclusions for the major clades.

### ﻿Taxonomy

#### ﻿1. Cathcartia

Taxon classificationPlantaeRanunculalesPapaveraceae

Hook.f. ex Hook., Curtis’s Bot. Mag. 77: t. 4596 (1851)

E55E89C2-E6E6-584F-9374-D54C9646E998

##### Type species.

*Cathcartiavillosa* Hook.f. ex Hook.

**Figure 2.**
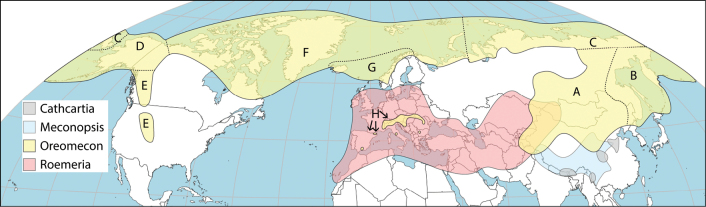
World distribution of the genera *Cathcartia* (grey), *Meconopsis* (sky blue), *Oreomecon* (yellow) and *Roemeria* (red). The total distribution of *Oreomecon* is divided into eight geographical areas (A–H) both on the map and in the textual treatment. Area E consists of two almost equally large subareas, while area H consists of one main subarea and four much smaller subareas (see arrows). Note also that the total distribution of *Cathcartia* consists of four different subareas. See Table 1 for the numbers of species and subspecies per genus and for each of the subareas of *Oreomecon*.

The genus includes four species as described by Grey-Wilson (2014), all with separate distribution areas in the East Himalayas and central parts of China, reproduced in Fig. 2. These species are accepted here.

#### ﻿2. Roemeria

Taxon classificationPlantaeRanunculalesPapaveraceae

Medik., Ann. Bot. (Usteri) 1(3): 15. 1792

7A3F5A0E-D075-5A34-BE06-11F9A2DB56BB

##### Type species.

*Roemeriaviolacea* Medik., nom. illeg. [≡ *R.hybrida* (L.) DC.]

##### Notes.

The genus is summarised here as including 16 species and one subspecies according to the treatment by Hassler (2023b). As shown by Fig. 2, the genus has a similar distribution to *Papaver*, but is absent from most parts of the Macaronesian Islands. It is present in Great Britain and southernmost Scandinavia, while it is absent from large parts of Central European Russia, the Arabian Peninsula and the Himalayan foothills. *Roemeria* may have had a long presence in Central Asia prior to the uplift of the Qinghai-Tibetan Plateau, based on its phylogenetic history illustrated in Fig. 1. Today, it extends as far east as Mongolia, represented by the species *R.refracta* DC. (Baasanmunkh et al. 2022).

#### ﻿3. Oreomecon

Taxon classificationPlantaeRanunculalesPapaveraceae

Banfi, Bartolucci, J.-M.Tison & Galasso, Nat. Hist. Sci. 9(1): 68. 2021

9EBB94AC-D471-59F3-B03E-B89294398CE8

##### Type species.

*Papaveralpinum* L., Sp. Pl. 507. 1753.

##### Notes.

When this genus was introduced by Banfi et al. (2022), the relationship between the basionym and the new name was not designated by adding the expected information “nom. et stat. nov.”. However, according to the International Code of Nomenclature for algae, fungi and plants (Shenzhen Code), this is a recommendation (Rec. 32A; Turland et al. (2018)) and not a requirement and the lack of this information does not affect the validity of the new genus. The species belonging to the recently described genus *Oreomecon* are treated here according to their distributions within eight geographical areas (see groups A–H below). Within each area, in cases where our interpretations deviate from the cited reference studies, they are discussed and used as a basis for the conclusive list of accepted species.

Overall, this treatment shows that *Oreomecon* contains 68 species and 29 subspecies. Only *O.lapponica* and *O.nudicaulis* are listed from more than one of the geographical areas defined above, however, with different subspecies. In the geography-based enumeration of species, only areas where the nominate subspecies occur are included. A total of 38 *Oreomecon* names are newly introduced below, 29 as recombinations and nine as nomenclatural novelties, whereas 21 existing *Oreomecon* names are put into synonymy.

##### Phylogeny of *Oreomecon*.

All ITS sequences found in the GenBank of taxa belonging to *Oreomecon* and some unpublished data were used to construct a state-of-the-art phylogram shown in the supporting document (Suppl. material 1). It is already known that this marker does not discriminate between closely-related taxa such as those of the *Oreomeconalpina* complex (Schönswetter et al. 2009). However, with this analysis, we wanted to check whether any major clades appeared within the genus. We found that the sequences included several misidentifications and that it was impossible to trace information on the origin of many of the samples. Our own alternative interpretations of the identities of most of the samples are, therefore, shown as a right-hand column in the figure.

Four clades were identified, although they should be interpreted with care. In branch I, three samples of *O.alpina* s.lat. are widely different from other samples. Branch IV probably includes cultivated material of “Iceland Poppy”, most commonly interpreted as *O.crocea* (Elven et al. 2011). In contrast, the single sample in Branch II might represent true *O.nudicaulis*, as it was labelled *P. nudicaule subsp. nudicaule* by Carolan et al. (2006). The most exciting result from the present phylogram was the concentration of taxa from Far East Asia in Branch III, although several samples remain uninterpreted.

### ﻿3.1. Non-Arctic Siberia and Central Asia

#### ﻿Notes

In her monograph on Siberian species, Peschkova (1994) accepted 30 species and two subspecies and provided distribution maps of all taxa. Her study is used as a reference for treating the geographical area that is dealt with here. However, as her geographical area also included Arctic parts of Siberia, five species and two subspecies from the Arctic are instead treated in group C below, together with other Asian Arctic species. Peschkova (1994) did not treat species from the Russian Far East, which is also in a geographical area different from the one dealt with here. Mongolia, the Central Asian republics and northern China, which belong to the present geographical area, were also outside the scope of her treatment. A recent flora checklist from Mongolia (Baasanmunkh et al. 2022) follows the species concepts of Peschkova (1994), except for two accepted species not present in Siberia: *Papaverbaitagense* Kamelin & Gubanov and *P.pseudotenellum* Grubov. However, Baasanmunkh et al. (2021) considered these species as synonyms of or very closely related to *P.croceum* Ledeb. and they are therefore synonymised below.

For a long time, the only species recognised from this area were *P.nudicaule* L. and *P.croceum* Ledeb. In addition, there were several taxa at infraspecific rank, two of which have intricate histories, which will be dealt with below. Papavernudicaulevar.rubro-aurantiacum Fisch. ex DC., was introduced by De Candolle (1821), although it has been treated as if it were a *nomen nudum* by several later authors. De Candolle (1821) had referred to a collection from Dahuria sent by Fischer, who apparently had suggested the name “in litt.”. However, later in his treatment, below a comparison of his three varieties of *P.nudicaule*, De Candolle (1821) presented a short diagnosis for var.rubro-aurantiacum, for which he wrote that it might represent a true new species. His name for this taxon is, therefore, accepted as a basionym here.

Peschkova (1994) cited the taxon as *P.rubro-aurantiacum* Fisch. ex R. Sweet. However, Sweet (1830) only listed the name at the species level in a horticultural magazine without any attempt at a taxonomic treatment, without citing the treatment by De Candolle (1821) and Sweet’s citation to the year “1822” instead refers to its introduction into British gardens. However, Hassler (2023b) cited the taxon as *P.rubro-aurantiacum* Fisch. ex Steud. Steudel (1841) only included the name in an enumeration where it was interpreted as a synonym of *P.croceum*. Fedde (1909) recombined the taxon as a subspecies. Lundström (1923) studied the holotype material collected in Dahuria by Fischer and sent to De Candolle, recombined the taxon at the species level and provided an extended description. His author citation deviates from the citation format followed by us, namely *P.rubroaurantiacum* (Fisch. ex DC.) C.E.Lundstr. The hyphen inserted by Lundström is deleted according to the Code (Article 60.11; Turland et al. (2018)). The name citation applied here is also in accordance with the treatment by POWO (2023). The type material was not explicitly cited by Lundström (1923), is not deposited at BG as indicated by Popov (1937) and Rändel (1974) and its herbarium affiliation is unknown to us.

Peschkova (1994) considered *P.rubroaurantiacum* to be heterogeneous, also including *P.ledebourianum* C.E.Lundstr., but mapped it as very common in southern Siberia, largely overlapping with the distribution area of *P.nudicaule*, but stated that they differ in flower colour and pubescence of sepals and capsules. Zhang and Grey-Wilson (2008), however, treated *P.rubroaurantiacum* as a synonym of *P.nudicaule*. Kamelin and Gubanov (1990) described *P.changaicum* Kamelin from Mongolia, differing from *P.rubroaurantiacum* by white flowers and it is treated as a synonym of the latter here. They also described P.rubroaurantiacumsubsp.chalchorum Kamelin from calcareous steppes in Mongolia, but this taxon was not accepted by Baasanmunkh et al. (2022).

Another early described taxon is *Papaverleiocarpum* (Turcz.) Popov, with its basionym Papavernudicaulevar.leiocarpum Turcz., published in 1838. IPNI (2023) cites the basionym as *P.leiocarpum* Turcz., referring to the same publication source, which agrees with the citation in Popov (1937). However, the original publication only lists “*P.lejocarpon* m.” which is a *nomen nudum* as it lacks an accompanying description. The significance of the added “m.” is unknown and possibly refers to a manuscript. Therefore, it appears that the publication source of the basionym cited by Peschkova (1994) as “1842–1845, Fl. Baic.-Dahur. 1: 98” instead represents its description. Krivenko (2023) cited the *nomen nudum* from 1838 instead of the basionym published by Peschkova (1994). However, the Code (Art. 41.6; Turland et al. (2018)) allows for erroneous basionym citations. Thus, the name *Oreomeconleiocarpa* (Turcz.) Krivenko is considered validly published, with the correct basionym citation supplied here.

In his treatment of *Papaver* for Flora SSSR, Popov (1937) included two Central Asian species of sect.Meconella described by Tolmachev (1931), as well as his own descriptions of five new species. Two of these, *Papaverpseudostubendorfii* Popov and *P.ajanense* Popov, were both synonymised with *P.stubendorfii* Tolm. by Peschkova (1994), whereas *P.pseudocanescens* Popov was accepted, while *P.involucratum* Popov was outside her study area. *Papavertianschanicum* Pavlov was introduced as a *nomen nudum* in 1933. When described by Popov a year later, the name *P.tianschanicum* Popov would have been more appropriately cited as “*Papavertianschanicum* Pavlov ex Popov”, as done by Hassler (2023b). However, as this is only an alternative in the Code (Art. 46.5; Turland et al. (2018)), this correction is not followed here. The species was re-described by Popov (1937) as an isonym and was accepted by Peschkova (1994). It was synonymised with *P.canescens* Tolm. by Zhang and Grey-Wilson (2008), but accepted by Galasso et al. (2023).

*Papaveramurense* (N.Busch) N.Busch ex Tolm. was accepted by Peschkova (1994). In contrast, Zhang and Grey-Wilson (2008) treated it as P.nudicaulef.amurense (N.Busch) H.Chuang. POWO (2023) and Hassler (2023b) cited the species as *P.amurense* (N.Busch) Karrer, based on Karrer (1935). However, the latter is a short and non-taxonomical notice in a horticultural magazine, without even an indirect reference to a basionym as required prior to 1953 by the Code (Art. 41.3; Turland et al. (2018)) and this is not a valid recombination. Tolmachev (1971) dealt specifically with this species, but did not cite its type.

A taxon known as *Papavertenellum* Tolm. was accepted by both Popov (1937) and Czerepanov (1995) and also by Peschkova (1994), although her statement that it “evidently represents a shade form of *P.pseudocanescens*” leads us to reject this species. Tolmachev (1930) treated it as *P.tenellum* (Korsh.) Tolm., based on a name “in sched.” by Korshinsky at LE. Its author citation is, therefore, given as ‘Korsh. ex Tolm.’ and the Korshinsky specimen illustrated by Tolmachev (1930) is designated as lectotype here. *Papavertenellum* was considered a synonym of *P.nudicaule* by Zhang & Grey-Wilson (2008) but accepted by Solstad et al. (2009) and POWO (2023). Following the argument by Peschkova (1994), *Oreomecontenella* (Tolm.) Krivenko is listed below as a synonym of *Papaverpseudocanescens* Popov.

Papaverrubroaurantiacumsubsp.longiscapum Rändel was recombined into species level by Krivenko (2023). However, the taxon was considered “simply the full-grown forms of *P.rubro-aurantiacum*” by Peschkova (1994) and her opinion is followed here. With the exceptions noticed above, we accept all species treated by Peschkova (1994), i.e. 23 species in total, of which six were described as new to science by her. The list also includes four species recombined by Peschkova (1994).

The diploid species *Papaverkuvajevii* Schaulo & Sonnikova (Shaulo and Sonnikova 2003) was described from a single locality in Krasnoyarsk Krai in Khakassia, Russia and is included in the list below. Only two more species have been described from the area south of Peschkova’s study area. The endemic *Papaverinvolucratum* Popov from 2800–3300 m alt. in the western Pamir-Alay mountains in Tajikistan has been accepted by most later authorities and is also included in the list below. It has also been collected from the Afghan side of the border as shown by GBIF (Global Biodiversity Information Facility) Secretariat (2023). *Papaverangrenicum* Pazij from the western Tian Shan mountains in Uzbekistan was accepted by Rändel (1974), based on several morphological characters. It has been treated as a synonym of *P.croceum* in flora lists by Czerepanov (1995) and Sennikov and Tojibaev (2021). The latter interpretation is followed here, although a re-study vs. neighbouring species is needed to finally reject the conclusion by Rändel (1974). Papaverinvolucratumsubsp.nigrescentihirsutum Tolm. was accepted by Solstad et al. (2009), but not by POWO (2023) nor by us. The report by Jafri and Qaiser (2011) of *Oreomeconnudicaulis* s.lat. from Pakistan probably refers to *O.crocea* in the sense of Peschkova (1994) and Sennikov and Tojibaev (2021). Notably, *Oreomecon* has not migrated westwards into the high mountains of Iran, Caucasus and Türkiye.

#### ﻿Distribution

Fig. 2 shows the distribution of the genus *Oreomecon* in this geographical area, which is considered to include 24 species. *Papavercroceum* was shown by Peschkova (1994) to have a south-western Siberian distribution pattern that does not overlap with the south-eastern one of *P.nudicaule*. *Papaveramurense* overlaps with *P.nudicaule* in south-eastern Siberia, but extends into the Russian Far East and southeastwards into China (Peschkova 1994). It also occurs in North Korea (Chang et al. 2014). *Papaverpseudocanescens* Popov and *P.rubroaurantiacum* were mapped as particularly widespread and abundant by Peschkova (1994).

#### ﻿Rare and red-listed species

*Papaverturczaninovii* is an endemic with a limited distribution south-east of Lake Baikal, while *P.kuvajevii* is only known from the two localities in East Sajan, as reported by Shaulo and Sonnikova (2003). An’kova et al. (2018) also briefly listed it from China, but no localities or samples were reported. In GBIF (Global Biodiversity Information Facility) Secretariat (2023), only the two localities by Shaulo and Sonnikova (2003) are available. The endemic *Papaverolchonense* and *P.popovii* were only mapped from the Lake Baikal area by Peschkova (1994), although with quite many localities and with a single locality of *P.olchonense* in the Republic of Sakha ca. 1,300 km north of the northern edge of Lake Baikal. *Papaverpopovii* was cited as rare and endangered along Lake Baikal shorelines by Bukharova et al. (2021). A new locality of this species was recently recorded from Agara River, ca. 500 km west of the northern edge of Lake Baikal as shown by GBIF (Global Biodiversity Information Facility) Secretariat (2023). *Papaverinvolucratum* was described as endemic and occurring in several areas of the western Pamir-Alai mountains in Tadzhikistan (Popov 1937). *Papaverturczaninovii* was treated as a stenotopic endemic by Peschkova (1994), being restricted to outcrops of marble limestones at the southeastern shore of Lake Baikal. Eight of the species were listed as Nearly Threatened (NT) in Russia by Xue et al. (2023), with only *P.tenellum* (included in *P.rubroaurantiacum* above) listed as Vulnerable (VU).

#### ﻿Accepted taxa

##### ﻿3.1.1. Oreomecon ammophila

Taxon classificationPlantaeRanunculalesPapaveraceae

(Turcz.) Krivenko, Nov. Syst. Pl. Vasc. 54: e06:1. 2023

F8344018-51E1-586E-A7B3-16014FE18FEA

≡ Papavernudicaulevar.ammophilum Turcz., Bull. Soc. Imp. Naturalistes Moscou 15: 98. 1842. Type: [Russia] Copiosissime crescit ad littus arenosum Baicalis prope monasterium Posdsolskoy, *N. Turczaninov* (not found) ≡ Papaverledebourianumvar.ammophilum (Turcz.) Peschkova, Fl. Tsentral’noi Sibiri 1: 378. 1979 ≡ Papaverammophilum (Turcz.) Peschkova, Fl. Sibir. 7: 16. 1994.

##### ﻿3.1.2. Oreomecon amurensis

Taxon classificationPlantaeRanunculalesPapaveraceae

(N.Busch) Galasso, Banfi & Bertolucci, Pl. Rev. 5(4): 58. 2023

44993722-20BB-53C6-9592-5A581EFF9AEC

≡ Papavernudicaulesubsp.amurense N.Busch, Fl. Sibir. Orient. Extremi 1: 21. 1913 ≡ Papaveramurense (N.Busch) N.Busch ex Tolm., Fl. Transbaikal 4: 410. 1941 ≡ Papavernudicaulef.amurense (N.Busch) H.Chuang, Fl. Reipubl. Popularis Sin. 32: 58. 1999.

##### ﻿3.1.3. Oreomecon canescens

Taxon classificationPlantaeRanunculalesPapaveraceae

(Tolm.) Krivenko, Nov. Syst. Pl. Vasc. 54: e06:2. 2023

1FB84176-B848-56AD-BE44-84F86B5EC0F6

≡ Papavercanescens Tolm., Zhurn. Russk. Bot. Obshch. 16: 77. 1931. Type: Sklony i vershiny khredta Saur [Kazakhstan, in jugo montium Saur], *Reznichenko* (LE: holotype).

##### ﻿3.1.4. Oreomecon chakassica

Taxon classificationPlantaeRanunculalesPapaveraceae

(Peschkova) Krivenko, Nov. Syst. Pl. Vasc. 54: e06:2. 2023

C685FD6E-E405-52B9-81D9-C674331D4A60

≡ Papaverchakassicum Peschkova, Fl. Sibir. 7: 18. 1994. Type: [Russia] Regio autonoma Chakassia distr. Askiz, in vicinitate vici Kamyschta, stepa lapidosa, 8 June 1970, *E. Erschova & T. Volkova s.n.* (holotype: NS)

##### ﻿3.1.5. Oreomecon crocea

Taxon classificationPlantaeRanunculalesPapaveraceae

(Ledeb.) Banfi, Bartolucci, J.-M.Tison & Galasso, Nat. Hist. Sci. 9(1): 71. 2022

11AF076B-6466-5796-928B-9639E4E5DB96

≡ Papavercroceum Ledeb., Fl. Altaic. 2: 271. 1830. Type: [Russia] Altai, *Ledebour* (lectotype: LE) ≡ Papavernudicaulevar.croceum (Ledeb.) Elkan, Tent. Monogr. Papaver 17. 1839 ≡ Papaveralpinumvar.croceum (Ledeb.) Ledeb., Fl. Ross. 1: 87. 1841.
= Papaverbaitagense Kamelin & Gubanov, Byull. Moskovsk. Obshch. Isp. Prir., Otd. Biol. 95(2): 86. 1990. Type: Jugo-zapadnaya Mongoliya, Dzhungariya, severnyi makrosklon khr. Baytag-Bogdo dolina r. Nariyn-Khargaityn-gol v 8 km na vostok ot zastavy Baitag-Bog-do Kobposkogo aimaka, 31 July 1988, *I.A.Gubanov & E.Gaubold 2562* (holotype: MW no. 0592489) ≡ Oreomeconbaitagensis (Kamelin & Gubanov) Krivenko, Nov. Syst. Pl. Vasc. 54: e06:2. 2023.
= Papaverangrenicum Pazij, Bot. Mater. Gerb. Bot. Inst. Uzbekistansk. Fil. Akad. Nauk S.S.S.R. 3: 31. 1941. Type: [Uzbekistan] Westlicher Tien-schan, Bassin des Flusses Angren, 15 Aug 1937, *Zakirov 173843* (TAK: holotypus).
= Papaverpseudotenellum Grubov, Bot. Mater. Gerb. Bot. Inst. Komarova Akad. Nauk S.S.S.R 17: 14. 1955. Type: [Mongolia] Altai Gobicus, jugum Gurban-Bogdo, mons Iche-Bogdo, fauces Narin-Churumt, latus orientale, ca. 2900 m, in fissuris rupium, 28 Aug 1948, *V. Grubov 6197* (holotype: LE).

##### ﻿3.1.6. Oreomecon involucrata

Taxon classificationPlantaeRanunculalesPapaveraceae

(Popov) Galasso, Banfi & Bertolucci, Pl. Rev. 5(4): 58. 2023

FF352DA9-C0BB-5CB8-B653-A7D998C979DA

≡ Papaverinvolucratum Popov; Fl. URSS 7: 748. 1937. Type: [Tadzhikistan] Asia Media, Pamir-Alai, ad fl. Zeravschan superior (holotype: LE).

##### ﻿3.1.7. Oreomecon jacutica

Taxon classificationPlantaeRanunculalesPapaveraceae

(Peschkova) Krivenko, Nov. Syst. Pl. Vasc. 54: e06:2. 2023

6D4172A5-E789-562E-BD7F-E58B743BA2E1

≡ Papavernudicaulesubsp.gracile Tolm., Bot. Mater. Gerb. Bot. Inst. Komarova Akad. Nauk SSSR 20: 166. 1960. Type: Russia, E Siberia, Yakutskaya ASSR, okrestnosti g. Yakutska, Urochishche Chuchur-Muran....na peschanoy pochve, 28 Jun 1956, *A.I. Tolmachev s.n*. (holotype: LE; typified by Elven et al. [2009], p. 989) ≡ Papaverjacuticum Peschkova, Fl. Sibir. 7: 19. 1994.

##### ﻿3.1.8. Oreomecon kuvajevii

Taxon classificationPlantaeRanunculalesPapaveraceae

(Schaulo & Sonnikova) Krivenko, Nov. Syst. Pl. Vasc. 54: e06:2. 2023

3FE008FF-946D-5378-AF43-AE0B8A4AAC6F

≡ Papaverkuvajevii Schaulo & Sonnikova, Turczaninowia 6(4): 5. 2003. Type: [Russia: Sajanum Occidentale. Jugum Chemtschikskij, declive generale septentrionis. Vallis fluminis Kolbak-Mis, circa ostium. Clivo montano saxoso. Schistosa]. 28 May 1982, *A.E. Sonnikova s.n.* (holotype: NS; isotype: SSB).

##### ﻿3.1.9. Oreomecon leiocarpa

Taxon classificationPlantaeRanunculalesPapaveraceae

(Turcz.) Krivenko, Nov. Syst. Pl. Vasc. 54: e06:2. 2023

93385971-79FE-5385-9CBE-B24949632BFF

≡ Papavernudicaulevar.leiocarpum Turcz., Fl. Baic.-Dahur.1: 98. 1842–1845. Type: Russia, ad torri Bugussony, *Kuznetzoff s.n*., 1834, (holotype: LE; isotypes: P [barcodes P00744601, P00744603], K [barcode K00065319]) ≡ Papaverleiocarpum (Turcz.) Popov., in V.L. Komarov (ed.) Fl. SSSR 7: 604. 1937.
– Papavernudicaulevar.leiocarpum Turcz., Bull. Imp. Naturalistes Moscou 11: 86. 1838, nom. nud.

##### ﻿3.1.10. Oreomecon leucotricha

Taxon classificationPlantaeRanunculalesPapaveraceae

(Tolm.) Krivenko, Nov. Syst. Pl. Vasc. 54: e06:2. 2023

84F5D719-D05A-5809-960D-A9ACDF8551EE

≡ Papaverleucotrichum Tolm., Bot. Mater. Gerb. Bot. Inst. Komarova Akad. Nauk SSSR. 20: 176. 1960. Type: [Russia], Siberia: Yakutia, [in jugo montium Tuora-Siss, ad ripam dextram fluminis Lenae inferioris, in cucumine montis Sokujdach], 11 Aug 1957, *B. Yurtsev and B. Norin* (holotype: LE).

##### ﻿3.1.11. Oreomecon nivalis

Taxon classificationPlantaeRanunculalesPapaveraceae

(Tolm.) Krivenko, Nov. Syst. Pl. Vasc. 54: e06:2. 2023

5FE2471C-7D0F-5229-811A-A4EC6F968E44

≡ Papavernivale Tolm., Svensk Bot. Tidskr. 24: 42. 1930. Type: [Russia] Werchojanski-Gebirge, im Tal des Tukulan, 914–977 m, 24 Jul 1935, *S. Nedrigailow* (lectotype: S, corresponding to major part of illustration in Tolmachev [1930: 41]; isolectotype: LE, designated here).

##### ﻿3.1.12. Oreomecon nudicaulis

Taxon classificationPlantaeRanunculalesPapaveraceae

(L.) Banfi, Bartolucci, J.-M.Tison & Galasso, Nat. Hist. Sci. 9(1): 71. 2022

28B58A44-8875-547A-965A-D694A6E9349A

≡ Papavernudicaule L., Sp. Pl.: 507. 1753. Type: J. Dillenius, Hortus Elthamiensis 1732, t. 224, fig. 291 (lectotype).

##### ﻿3.3.13. Oreomecon nudicaulis subsp. nudicaulis

Taxon classificationPlantaeRanunculalesPapaveraceae

(L.) Banfi, Bartolucci, J.-M.Tison & Galasso, Nat. Hist. Sci. 9(1): 71. 2022

DE9F3A04-45B0-5C0D-A7CA-D9988EC39F94

≡ Papavernudicaule L., Sp. Pl.: 507. 1753. Type: J. Dillenius, Hortus Elthamiensis 1732, t. 224, fig. 291 (lectotype).

##### ﻿3.1.14. Oreomecon olchonensis

Taxon classificationPlantaeRanunculalesPapaveraceae

(Peschkova) Galasso, Banfi & Bertolucci, Pl. Rev. 5(4): 58. 2023

BBC7A405-CC9D-5D27-8F08-3C424BDEB010

≡ Papaverolchonense Peschkova, Fl. Sibir. 7: 23. 1994. Type: [Russia: Ora Maris Minoris (“Malomorskoe”) lacus Baical, ins. Olchon, prope vicum Chonchoi, declive boreali-occidentale], 17 Jun 1957, *G. Peschkova s.n.* (holotype: NSK).

##### ﻿3.1.15. Oreomecon popovii

Taxon classificationPlantaeRanunculalesPapaveraceae

(Sipliv.) Galasso, Banfi & Bertolucci, Pl. Rev. 5(4): 58. 2023

58A2E4EE-3C08-55C0-9E36-02523F84314E

≡ Papaverpopovii Sipliv., Novosti Sist. Vyssh. Rast. 10: 360. 1973. Type: [Russia] Baikal, Chivyrkuisky zaliv, ostrov Lokhmatyi Kaltygei, skaly severnogo berega, 8 Sep 1971, *V.N. Siplivinskiy s.n.* (holotype LE; isotype TK).

##### ﻿3.1.16. Oreomecon pseudocanescens

Taxon classificationPlantaeRanunculalesPapaveraceae

(Popov) Galasso, Banfi & Bertolucci, Pl. Rev. 5(4): 58. 2023

A3C0BE3D-FB92-51E7-8E58-A3C3EF0C7D33

≡ Papaverpseudocanescens Popov, in V.L. Komarov (ed.) Fl. SSSR 7: 749. 1937. Type: [Russia] Altai, in alpinis fluvii Topczugan, 1913, *Kusnetzov and Tripolitova 2670* (holotype: LE).
= Papavertenellum Korsh. ex Tolm., Sv. Bot. Tidskr. 24: 40. 1930, syn. nov.; Type: [Kasakhstan] Karkaraly-Gebirge, in schattigen Schluchten, 18–20 Jun 1890, *Korshinsky* (lectotype: LE, illustrated by Tolmachev [1930: 40], designated here) ≡ Oreomecontenella (Tolm.) Krivenko, Nov. Syst. Pl. Vasc. 54: e06:3. 2023.

##### ﻿3.1.17. Oreomecon rubroaurantiaca

Taxon classificationPlantaeRanunculalesPapaveraceae

(Fisch. ex DC.) Krivenko, Nov. Syst. Pl. Vasc. 54: e06:3. 2023

12C0A182-B3E0-50A5-A2C7-A5C97C9633F0

≡ Papavernudicaulevar.rubroaurantiacum Fisch. ex DC., Syst. Nat. 2: 70. 1821; Type: Baikalien (Dahurien), *Fischer* (holotype) ≡ Papaverrubroaurantiacum (Fisch. ex DC.) C.E.Lundstr., Acta Horti Berg. 7: 417. 1923
≡ Papavernudicaulesubsp.rubroaurantiacum (Fisch. ex DC.) Fedde, in Engler, H.G.A. (ed.) Pflanzenr. IV, 104: 381. 1909.
= Papaverrubroaurantiacumsubsp.longiscapum Rändel, Feddes Repert. 84, 9–10: 683. 1974. Type: [Russia] Im Tal des Flusses Amur, am Berghang, Nähe der Siedlung Dshilinda, 17 Jun 1913, *Kazanskij* (holotype [‘lectotype]’) ≡ Oreomeconlongiscapa (Rändel) Krivenko, Nov. Syst. Pl. Vasc. 54: e06:2. 2023.
= Papaverchangaicum Kamelin, Byull. Moskovsk. Obshch. Isp. Prir., Otd. Biol. 95(2): 87. 1990. Type: Mongolia centralis, regio Uber-Changai, ad oriente ab urb. Charcharin (Karakorum), in valle fl. Tarany-gol prope montem Cecerleg-ula, 24 Jul 1983, *I.A.Gubanov 7496* (holotype: MW).
= Papaverrubroaurantiacumsubsp.chalchorum Kamelin, Byull. Moskovsk. Obshch. Isp. Prir., Otd. Biol. 95(2): 88. 1990. Type: Mongolia centralis, steppa chalchorum, mons Saan-Schire, ca. 80 km in via Under-Chan-Manchan, 19 Jun 1987, *A.L.Budantzev et al. 20* (holotype: LE).

##### ﻿3.1.18. Oreomecon saichanensis

Taxon classificationPlantaeRanunculalesPapaveraceae

(Grubov) Krivenko, Nov. Syst. Pl. Vasc. 54: e06:3. 2023

A24F5882-1627-58C1-8692-DD796BBBA66E

≡ Papaversaichanense Grubov, Bot. Mater. Gerb. Bot. Inst. Komarova Akad. Nauk. SSSR 17: 15. 1955. Type: [Mongolia] Altai Gobicus, jugum Gurban-Saichan, mons Dzun-Saichan, in faucibus Jalo-Ama, ad fl. Tzagan-Gol, sub rupibus, 20 Aug 1931, *N. Ikonnikov-Galitzky 4192* (holotype: LE) ≡ Papaverrubroaurantiacumsubsp.saichanense (Grubov) Kamelin & Gubanov, in Gubanov I.A. Konsp. Fl. Vneshnei Mongolii: 52. 1996.

##### ﻿3.1.19. Oreomecon setosa

Taxon classificationPlantaeRanunculalesPapaveraceae

(Tolm.) Krivenko, Nov. Syst. Pl. Vasc. 54: e06:3. 2023

75710126-F48A-5113-9BA2-04B2737313AC

≡ Papaverrubroaurantiacumsubsp.setosum Tolm., Svensk Bot. Tidskr. 24: 39. 1930 ≡ Papaversetosum (Tolm.) Peschkova, Stepnaya Fl. Baikal’skoi Sibiri: 59. 1972.
= Papaveralpinumvar.hispidissimum Ledeb., Fl. Ross. 1: 87. 1842 ≡ Papaveranomalumvar.hispidatissimum (Ledeb.) Tolm., Novosti Sist. Vyssh. Rast. 7: 157. 1971;

##### ﻿3.1.20. Oreomecon smirnovii

Taxon classificationPlantaeRanunculalesPapaveraceae

(Peschkova) Krivenko, Nov. Syst. Pl. Vasc. 54: e06:3. 2023

594219F1-21D7-5CBD-91A9-E4C5F624CC95

≡ Papaversmirnovii Peschkova, Novosti Sist. Vyssh. Rast. 14: 239. 1977. Type: [Russia] Systema fl. Onon, in viciniis pag. Czindant-2, locus «Zavodskaya», in declivi schistoso-stepposo, 30 May 1911, *V. Smirnov 270* (holotype and four isotypes: LE).

##### ﻿3.1.21. Oreomecon stanovensis

Taxon classificationPlantaeRanunculalesPapaveraceae

(Petroch.) Krivenko, Nov. Syst. Pl. Vasc. 54: e06:3. 2023

A5DEC9E0-B166-537C-B47C-E66140685F68

≡ Papavercroceumsubsp.stanovense Petroch., in L.I. Malyschev (ed.). Vysokogornaya Fl. Stanovogo Nagor’ya 96. 1972. Type: Russia, Buryatiya, Stanovoe Nagorye, Yuzhno-Muyskiy mountain range, the origins of the Barguzin River, in the alpine zone,1900 m alt., on the convex matted gravelly calcareous slope, 55°N, 111°E., 19 Aug 1968, *Yu. Petrochenko 513* (NSK: holotype) ≡ Papaverstanovense (Petroch.) Peschkova, Fl. Sibir. 7: 28. 1994.

##### ﻿3.1.22. Oreomecon stubendorfii

Taxon classificationPlantaeRanunculalesPapaveraceae

(Tolm.) Krivenko, Nov. Syst. Pl. Vasc. 54: e06:3. 2023

9D769AB0-8612-551F-88B4-48283948FB7C

≡ Papaverstubendorfii Tolm., Zhurn. Russk. Bot. Obshch. 16: 80. 1931. Type: [Russia] Allakh-jun, na puti iz Jakutska v Okhotsk, *Shtubendorf* (LE: holotype).

##### ﻿3.1.23. Oreomecon tianschanica

Taxon classificationPlantaeRanunculalesPapaveraceae

(Popov) Galasso, Banfi & Bertolucci, Pl. Rev. 5(4): 58: 2023

8663D780-4055-5A06-9E54-CE807BE83899

≡ Papavertianschanicum Popov, Trudy Sredne-Aziatsk. Gosud. Univ., Ser. 8b, Bot. 17: 84. 1934. Type: [Kyrgyzstan] Asia Media, Tian-Schan, Jugum Kungei-Alatau, ad fl. Kebin, *Abolin 3264* (LE); Papavertianschanicum Popov, in V.L. Komarov (ed.) Fl. SSSR 7: 748. 1937, isonym. ≡ Papavercroceumsubsp.tianschanicum (Popov) Kamelin, Fl. Ushchel. Reki Varzob: 140. 2021.
– Papavertianschanicum Pavlov, Byull. Moskovs. Obshch. Isp. Prir., Otd. Biol. 1933. n.s. xiii: 126, nom. nud.

##### ﻿3.1.24. Oreomecon turczaninovii

Taxon classificationPlantaeRanunculalesPapaveraceae

(Peschkova) Krivenko, Nov. Syst. Pl. Vasc. 54: e06:3. 2023

97E1B4BD-6625-5C55-BA80-5F3D738A023B

≡ Papaverturczaninovii Peschkova, Fl. Sibir. 7: 30. 1994, nom. nov. Papavernudicaulevar.calcareum Peschkova, Fl. Tsentr. Sib. 1: 379. 1979. Type: Russia, Irkutskaya Oblast’, Hamar-Daban mountain range, River Slyudyanka, talus near the marble quarry, 51°N, 105°E, 15 Jul 1964, *M. Ivanova* (holotype: NSK).

##### ﻿3.1.25. Oreomecon udocanica

Taxon classificationPlantaeRanunculalesPapaveraceae

(Peschkova) Krivenko, Nov. Syst. Pl. Vasc. 54: e06:3. 2023

584F33CD-F1DC-5FC2-9918-C19E7D7E2EE9

≡ Papaverpseudocanescenssubsp.udocanicum Peschkova, Novosti Sist. Vyssh. Rast. 14: 238. 1977. Type: Russia, Chitinskaya Oblast’, Stanovoye Nagorje, Udokan Ridge, the upper reaches of Naminga River, at the upper border of the forest, 1350 m alt., on the river gravel, 56°34'41" N, 118°29'58" E, 26 Jun 1964, *L. Malyschev & Yu. Petrochenko 350* (holotype: LE; isotype: NSK) ≡ Papaverudocanicum (Peschkova) Peschkova, Fl. Sibir. 7: 30. 1994.

### ﻿3.2. The Asian Far East

#### ﻿Notes

This area is defined as comprising north-eastern China (Liaoning, Jilin, Heilongjiang), the Korean Peninsula, Japan and non-Arctic areas of the Russian Far East, the latter defined as the eight easternmost administrative units in Russia. This area does not overlap geographically with the administrative units comprising Siberia, as dealt with by Peschkova (1994). The *Papaver* flora of the former Soviet Far East was presented by Bezdeleva (1987) and later updated for the Russian Far East by Bezdeleva et al. (2006). These two studies are used here as a combined reference study for this geographical area.

Altogether, these studies included 34 species, 30 of them presented with distribution maps, although the majority of the species are from the Arctic parts. In the present treatment, the latter are included in the next geographic group, except for three species with most of their occurrences on the American side. Therefore, they are treated in the group of Arctic Alaskan and Yukon species. Only a few of the Siberian species treated by Peschkova (1994) have marginal occurrences within the presently-defined area. To conclude, seven species dealt with by Bezdeleva (1987) and Bezdeleva et al. (2006) are exclusive to the presently-defined geographical area and are commented on below.

The earliest name from the area is *Papavermicrocarpum* DC. described from Kamchatka by De Candolle (1821), based on a P.S. Pallas collection, although the true collector was possibly C.H. Merk in 1788, who passed his plant collections over to Pallas (Yakubov et al. 2001). Tolmachev (1931) described *Papaverochotense* Tolm., but later, Tolmachev (1975) recombined it as one of four subspecies of *P.microcarpum*, another one being P.microcarpumsubsp.alaskanum (Hultén) Tolm. In his distribution map, Papavermicrocarpumsubsp.ochotense (Tolm.) Tolm. was shown to have a distinct southern distribution compared to P.microcarpumsubsp.microcarpum with an Arctic distribution in Chukotka and P.microcarpumsubsp.czekanowskii (Tolm.) Tolm., both occurring from Chukotka to much further to the west in Arctic Yakutia. These distribution patterns were confirmed by Petrovsky (1999).

These studies dealt with the Arctic and, for the Far East, Bezdeleva (1987) mapped *Papavermicrocarpum* southwards to the Magadan area and southernmost parts of Kamchatka as a widely defined species without accepted subspecies, a concept also followed by Bezdeleva et al. (2006). Grey-Wilson (2023) did not even accept *P.macrocarpum* as a separate species, but recombined it as a subspecies of *Oreomeconnudicaulis*. Czerepanov (1995) and Pavlova (1999) accepted *Papaverochotense* Tolm. as a separate species, whereas Krivenko (2023) did not recognise this taxon, but recombined *Papaverczekanowskii* Tolm. at the species level in *Oreomecon*.

Elven et al. (2011) treated the complex as three subspecies, but added that “it may consist of three (or more) separate species” and cited unpublished studies where *P.czekanowskii* and *P.microcarpum* s.str. were surprisingly different genetically. They also mentioned *Papaveromolonense* Khokr., supposedly described from the Magadan area, a name we could not find in any of the cited sources. Solstad et al. (2009) also reported on a genetically distinct taxon from Karaginsky Island off northern Kamchatka referred to as “P.sp. aff.microcarpum”. It is tetraploid, whereas *microcarpum* is diploid. Following the hypothesis by Elven et al. (2011) and the evolutionary pattern within the genus in the Asian Far East, the three taxa are treated as separate species within *Oreomecon* here, with *O.microcarpa* (DC.) Krivenko as primarily a non-Arctic species. Chukotkan specimens of P.microcarpumsubsp.microcarpum are left for interpretation by future studies.

Another early name from the area is *Papaveranomalum* Fedde (Fedde 1909). It was accepted by Popov (1937), who lumped it with P.nudicaulesubsp.amurense N.Busch., a conclusion which V. Komarov (1937), the Editor of “Flora SSSR”, opposed. Rändel (1974) agreed that *P.anomalum* sensu Fedde is different from *P.amuren*se. However, she treated it as a subspecies of *P.croceum*. Bezdeleva (1987) provided a key separating this species from its most closely-related species, *P.amurense* and mapped the latter as very common in the southern part of the Far East, particularly near the Chinese border, whereas *P.anomalum* is much rarer. Peschkova (1994) and Zhang & Grey-Wilson (2008) omitted *P.anomalum* from their studies, but Peschkova (1994) treated P.anomalumvar.hispidissimum (Ledeb.) Tolm. as a synonym of the Siberian *P.setosum* (Tolm.) Peschkova. *Papaveranomalum* was recombined into *Oreomecon* by Banfi et al. (2022) and POWO (2023) and Hassler (2023b) accepted this name. As emphasised by Fedde (1909), the species is very distinct by its almost globose, mostly glabrous capsules and the name is lectotypified here, based on a specimen in B where both capsule and flowers are developed. This sheet would have been readily available to Fedde and apparently carries his handwriting.

*Papaveralboroseum* Hultén was not typified by Hultén (1928) and, as shown by Björk (2019), two duplicates of the type exist. Here, we designate the collection at S as lectotype, as capsules are much better developed than on the alternative sheet at GB. The latter is defined as isolectotype here, related to its previous and unpublished annotation as “isotype”. According to Yakubov et al. (2001), *P.alboroseum* and *P.microcarpum* co-occur on the Avachinsky Volcano in Kamchatka, where both species are frequent.

Pavlova (1999) described *P.tolmatschevianum* N.S.Pavlova from Sakhalin. This species had already been described and illustrated in Flora of Sakhalin by Sugawara (1937–1940) under the name *Papaverochotense* Miyabe & Tatew., which is an illegitimate homonym of *P.ochotense* Tolm., described by Tolmachev (1931).

Krivenko (2023) recombined Papaveranomalumvar.hirsutum Tolm. as *Oreomeconhirsuta* (Tolm.) Krivenko without making any reference to the synonym *Papaversokolovskajae* Prob., which apparently has priority at the species level. Probatova in Bezdeleva et al. (2006) described *P.sokolovskajae* as a white-flowered species from supralittoral habitats along the coast near Vladivostok. It has conspicuously glabrous and subglobose capsules, a character also noted by Krivenko (2023). However, *P.sokolovskajae* was not described as a new species, but as a nom. nov. and stat. nov. with Papaveranomalumvar.hirsutum Tolm. described by Tolmachev (1971) as the basionym. Probatova did not give any arguments for introducing a replacement name and we cannot find any existing and competing “*Papaverhirsutum*” name justifying the choice. Based on the Code (Art. 6.10–11; Turland et al. (2018)), we therefore consider *P.sokolovskajae* as an illegitimate name and instead follow the recombination made by Krivenko (2023).

For the “Flora of China”, Zhang and Grey-Wilson (2008) listed Papaverradicatumvar.pseudoradicatum (Kitag.) Kitag. from above 1,600 m alt. on Changbai Shan in the Province of Jilin close to North Korea, also listing the taxon from Korea. Bezdeleva et al. (2006) mapped a single locality of *P.pseudoradicatum* Kitag. from the Russian side and POWO (2023) indicated this taxon to occur in Korea, “Manchuria” and the “administrative region of Khabarovsk” in the Russian Far East. Chang et al. (2014) also included *P.pseudoradicatum* from North Korea in their list of species from Korea.

Lee et al. (2011) reported *P.coreanum* Nakai to be “widely distributed in an alpine belt of Baekdu/Changbaek”, a mountain chain shared by North Korea and China, also referred to as Paektu-san in Korea and Changbai Shan in China. They also cited the species to be protected in China, although the species name applied was not indicated. Zhang and Grey-Wilson (2008) and Chang et al. (2014) did not mention *P.coreanum*, whereas POWO (2023) listed both *P.coreanum* and *P.pseudoradicatum*. The Flora of Korea (Kim 2017) accepted *P.coreanum*, but did not mention *P.pseudoradicatum*. Due to their identical distributions in North Korea and the adjacent parts of China and their highly similar morphological descriptions, it is possible that these reports refer to the same species. In that case, *P.coreanum*, described in 1928, would have priority over *P.pseudoradicatum*, described in 1942. Krivenko (2023) accepted both these species and recombined them in *Oreomecon*. We accept *P.coreanum* and provisionally place *P.pseudoradicatum* in synonymy pending future studies, but have not been able to cite their types.

A species from the Kurile Islands known as *P.miyabeanum* Tatew. is closely related to *P.fauriei* (Fedde) Fedde ex Miyabe & Tatew., a local endemic from Rishiri, a volcanic island just west of the northernmost tip of Hokkaido in Japan (Takahashi and Yamagishi 2020). The former was recombined into *Oreomecon* by Banfi et al. (2022), citing a basionym originating from Miyabe and Tatewaki (1936). However, the year before, Miyabe and Tatewaki (1935) had described Papavernudicaulesubsp.xanthopetalumvar.shimshirense Miyabe & Tatewaki listing the new name *P.miyabeanum* Tatewaki as a synonym “in sched.”. Takahashi and Yamagishi (2020) restudied the complex and reduced the former to a subspecies of *P.fauriei* under the name P.faurieisubsp.shimshirense (Miyabe & Tatew.) Hideki Takah. They concluded that the simultaneous publication of *P.miyabeanum* as a synonym of Papavernudicaulesubsp.xanthopetalumvar.shimshirense by Miyabe and Tatewaki (1935) made the former name illegitimate, also when Miyabe and Tatewaki (1936) intended to name the taxon at the species level and when the taxon was recombined as *Oreomeconmiyabeana* (Art. 6.4 and 58.1 in the Code; Turland et al. (2018)). *Papaverfauriei* was originally published as Papavernudicaulesubsp.xanthopetalumvar.fauriei Fedde by Fedde (1909), who defined the specimen *Faurie 3015* at B as the holotype, which, according to Takahashi and Yamagishi (2020), has not been relocated and only isotypes are therefore listed below.

#### ﻿Distribution

We conclude that this area includes nine species and one subspecies. The distribution of the genus *Oreomecon* in this area is shown in Fig. 2.

#### ﻿Rare or red-listed taxa

Yamagishi et al. (2010, 2018) reported a small population of P.faurieisubsp.fauriei to be Endangered (EN) due to the threat of hybridisation with a cultivated and undetermined *Papaver* sp., which is determined here as P.faurieisubsp.shimshirense based on the data shown by Takahashi and Yamagishi (2020) and the results in our phylogram. This would then be a case of infraspecific hybridisation. Xue et al. (2023) listed *P.anomalum* as Vulnerable (VU) in Russia under the name P.nudicaulevar.aquilegifolium Fedde, which is considered a synonym of *P.ammophilum* by POWO (2023). *Papaveranadyrense* V.V.Petrovsky and *P.tolmatschevianum* were listed as Nearly Threatened (NT) by Xue et al. (2023).

#### ﻿Accepted taxa

##### ﻿3.2.1. Oreomecon alborosea

Taxon classificationPlantaeRanunculalesPapaveraceae

(Hultén) Galasso, Banfi & Bertolucci, Pl. Rev. 5(4): 58. 2023

27DC9F4C-4880-5522-AA96-82946422431D

≡ Papaveralboroseum Hultén, Kongl. Svenska Vetensk. Akad. Handl., Ser. 3, 5(2): 141. 1928. Type: [Russia] Kamtchatka australis, Avatcha Volcano, 675 m alt., 30 Jul 1920, *E. Hultén 508b* (lectotype: S [no. S-G-4522] lectotype designated here; isolectotype: GB [barcode GB0048356]).

##### ﻿3.2.2. Oreomecon anadyrensis

Taxon classificationPlantaeRanunculalesPapaveraceae

(V.V.Petrovsky) Krivenko, Nov. Syst. Pl. Vasc. 54: e06:1. 2023

0159D900-FF81-50C4-A568-0E8ED56FC2DD

≡ Papaveranadyrense V.V.Petrovsky, Bot. Zhurn. (Moscow & Leningrad) 68: 229. 1983. Type: [Russia] Terra Tschuktschorum australis, districtus Anadyrensis, prope pagum Otrozhnyj, in valle fl. Mavrina, in summitate monticuli, tundra schistosa, in Dryadeta, 14 Aug 1977, *P. Zhukova 77-379* (holotype: LE).

##### ﻿3.2.3. Oreomecon anomala

Taxon classificationPlantaeRanunculalesPapaveraceae

(Fedde) Banfi, Bartolucci, J.-M.Tison & Galasso, Nat. Hist. Sci. 9(1): 71. 2022

59794236-3DE8-5738-B905-CA44FB0895E1

≡ Papaveranomalum Fedde, Pflanzenr. (Engler) IV.104(40).1909. Type: Central China, West Hupeh, Jun 1901, *E.H. Wilson 2421* (lectotype: B [barcode B 10 0279403], lectotype designated here; isolectotypes: P [barcode P00738904], US [barcode US00099714] and LE).

##### ﻿3.2.4. Oreomecon coreana

Taxon classificationPlantaeRanunculalesPapaveraceae

(Nakai) Krivenko, Nov. Syst. Pl. Vasc. 54: e06:2. 2023

A4D0E710-432B-56A7-ABE3-9BCC86321B20

≡ Papavercoreanum Nakai, Sci. Knowl. 8: 42. 1928.
= Papaverpseudoradicatum Kitag., Rep. Inst. Sci. Res. Manchoukuo, 6, 4: 122. 1942, “pseudo-radicatum”, **syn. nov.** ≡ Papaverradicatumvar.pseudoradicatum (Kitag.) Kitag., Neolin. Fl. Manshur.: 325 (1979) ≡ Oreomeconpseudoradicatum (Kitag.) Krivenko, Nov. Syst. Pl. Vasc. 54: e06:2. 2023.

##### ﻿3.2.5. Oreomecon fauriei

Taxon classificationPlantaeRanunculalesPapaveraceae

(Fedde) Galasso, Banfi & Bertolucci, Pl. Rev. 5(4): 58. 2023

24392B1C-2AEF-535A-9738-EA5E2570A7CC

≡ Papavernudicaulesubsp.xanthopetalumvar.fauriei Fedde, Repert. Spec. Nov. Regni Veg. 7: 257. 1909. Type: Japan, Hokkaido, Isl. Rishiri, *Faurie 3015*, 25 Jul 1899 (isotypes KYO; P [barcode P00744502] and P. [barcode P00744599] ≡ Papaverfauriei (Fedde) Fedde ex Miyabe & Tatew., Trans. Sapporo Nat. Hist. Soc. 14: 258.1936.

##### ﻿3.2.6. Oreomecon fauriei subsp. fauriei

Taxon classificationPlantaeRanunculalesPapaveraceae

(Fedde) Galasso, Banfi & Bertolucci, Pl. Rev. 5(4): 58. 2023

F4DB6F67-A5D2-5E3C-8552-FC3B16FE5CD4

≡ Papavernudicaulesubsp.xanthopetalumvar.fauriei Fedde, Repert. Spec. Nov. Regni Veg. 7: 257. 1909. Type: Japan, Hokkaido, Isl. Rishiri, *Faurie 3015*, 25 Jul 1899 (isotypes KYO; P [barcode P00744502] and P [barcode P00744599].

##### ﻿3.2.7. Oreomecon fauriei subsp. shimshirensis

Taxon classificationPlantaeRanunculalesPapaveraceae

(Miyabe & Tatew.) Elvebakk & Bjerke comb. nov.

8D3787D8-1038-5314-B6C3-83A774DC1823

urn:lsid:ipni.org:names:77351066-1

≡ PapavernudicauleL.subsp.xanthopetalumFeddevar.shimshirense Miyabe & Tatew., Trans. Sapporo Nat. Hist. Soc. 14: 5. 1935. Type: Middle Kurils, Isl. Shimshir, Broughtonzaki, *M. Tatewaki & Y. Tokunaga 11569*, 13 Aug 1928 (SAPS no. 036731: holotype) ≡ Papaverfaurieisubsp.shimshirense (Miyabe & Tatew.) Hideki Takah, Acta Phytotax. Geobot. 71: 154. 2020.
– Papavermiyabeanum Tatew., Trans. Sapporo Nat. Hist. Soc. 14: 259. 1936, nom. illeg.; – Oreomeconmiyabeana (Tatew.) Banfi, Bartolucci, J.-M.Tison & Galasso, Nat. Hist. Sci. 9(1): 71, 2022, nom. illeg.

##### ﻿3.2.8. Oreomecon hirsuta

Taxon classificationPlantaeRanunculalesPapaveraceae

(Tolm.) Krivenko, Nov. Syst. Pl. Vasc. 54: e06:2. 2023

F7EB092F-A7E4-522A-BBCE-B78CA3FBA4F0

≡ P.anomalumvar.hirsutum Tolm., Novosti Sist. Vyssh. Rast. 7: 157. 1971. Type: [Russia] Primorskiy Krai, Pos’etskiy r.-n., p[aluostr]ov Peschanyi, Kosa, 31 Jul 1931, *V. Petrov* (holotype: LE) ≡ Papaversokolovskajae Prob., Fl. Ross. Dal’nego Vostoka: 63. 2006, nom. illeg.

##### ﻿3.2.9. Oreomecon microcarpa

Taxon classificationPlantaeRanunculalesPapaveraceae

(DC.) Krivenko, Nov. Syst. Pl. Vasc. 54: e06:2. 2023

EEDD6F2C-E430-5912-BD17-6578CC7C1643

≡ Papavermicrocarpum DC., Syst. Nat. 2: 71. 1821. Type: Kamchatka, *P.S. Pallas* (holotype: G-DC-166725/1) ≡ Papavernudicaulesubsp.microcarpum (DC.) Elkan, Monogr. Papav.: 17. 1839 ≡ Papaveralpinumvar.microcarpum (DC.) Ledeb., Fl. Ross. 1: 87. 1841 ≡ Oreomeconnudicaulissubsp.microcarpa (DC.) Grey-Wilson, Pl. Rev. 5 (4): 57. 2023.

##### ﻿3.2.10. Oreomecon ochotensis

Taxon classificationPlantaeRanunculalesPapaveraceae

(Tolm.) Elvebakk & Bjerke comb. nov.

C772203C-85ED-523A-B9B8-F88375CC2DC5

urn:lsid:ipni.org:names:77350924-1

≡ Papaverochotense Tolm., Zhurn. Russk. Bot. Obshch. 16: 82. 1931. Type: [Russia] Bassein r. Penshiny. Na krutom kamenistom sklone k r. Pal’matkinoy v 22 km ot ust’ya. 7 Aug 1930, *V.B. Sochava* (holotype: LE) ≡ Papavermicrocarponsubsp.ochotense (Tolm.) Tolm., in V.L. Komarov (ed.) Fl. SSSR 7: 31. 1975.

##### ﻿3.2.11. Oreomecon tolmatscheviana

Taxon classificationPlantaeRanunculalesPapaveraceae

(N.S.Pavlova) Krivenko, Nov. Syst. Pl. Vasc. 54: e06:3. 2023

763EA369-16E2-5B05-B784-B794D758C96E

≡ Papavertolmatschevianum N.S. Pavlova, Bot. Zhurn. (Moscow & Leningrad) 84 (2): 112. 1999. Type: [Russia: Insula Sachalin, districtus Poronajskij, brachia orientalia montium Sachalinensium Orientalium, cacumen montis Slannikovi (343 m s. m.), 2–3 km ad boreali-occidentem a promontorio Sheltingi, regio subalpina, in locis schistosis in pineto raro (Pinuspumila)], 16 Aug.1991, *N. S. Pavlova s.n.* (holotype: VLA; isotype: LE).
– Papaverochotense Miyabe & Tatew., in Sugawara, S. Ill. Fl. Saghal. 3: 985. 1940, homonym.

### ﻿3.3. Arctic Asia

#### ﻿Notes

The primary treatment of this group is Tolmachev (1975) in *Arkticheskaya Flora SSSR*. He included 16 species and seven additional subspecies and he was the author or co-author of no less than 19 of these taxa, the first ones described 52 years earlier, in 1923. Two species were described together with his pupil V.V.Petrovsky (Tolmachev and Petrovsky 1973), who continued with *Papaver* studies until his early 90s, also representing an almost 50-year-long career. After the passing-away of Tolmachev in 1979, another nine species and one subspecies were described by Petrovsky (1983, 1985). The study by Tolmachev (1975), which includes an identification key and distribution maps and the two studies by Petrovsky are here considered as a combined monograph, covering 24 species and eight subspecies. The treatment below only includes new or deviating information on the cited reference studies.

The species *Papaveranadyrense*, *P.leucotrichum*, *P.microcarpum*, *P.nivale* and *P.ochotense* listed by the cited monograph source have been removed from this part, as they do not or scarcely reach the Arctic (Elven et al. 2011) and *P.microcarpum* was discussed above as a primarily non-Arctic species. They are here, instead, treated in other geographical sections. Despite being accepted by POWO (2023), *Papaverminutiflorum* Tolm. had been synonymised with P.lapponicumsubsp.orientale Tolm. by Petrovsky (1999) and Elven et al. (2011). These sources are followed here, whereas the recently-recombined name *Oreomeconminutiflora* (Tolm.) Krivenko and the recently-changed status of *O.orientalis* (Tolm.) Krivenko are both placed in synonymy. *Papaverindigirkense* Jurtzev was synonymised with *P.minutiflorum* by Peschkova (1994). Papaverradicatumsubsp.occidentale C.E.Lundstr., mapped from Wrangell Island and the Chukotka Peninsula by Tolmachev (1975), was subsumed under *P.radicatum* Rottb. by POWO (2023). However, Petrovsky (1999) had already explained a broad interpretation of *P.radicatum* as a confusion with several morphologically similar Siberian species instead. This was in agreement with Solstad et al. (1999), who considered *P.radicatum* to be a Nordic species.

The remaining taxa from the monograph source used here were accepted by Elven et al. (2011), although four of them only provisionally. However, the need to better understand most of the taxa was underlined. Petrovsky et al. (2019) described three subspecies of the *P.pulvinatum* Tolm. complex. The previously misinterpreted name P.pulvinatumsubsp.lenaense Tolm. was shown by Petrovsky et al. (2019) to be a synonym of P.nudicaulevar.riparium V.V.Petrovsky. Chepinoga et al. (2023) lifted these subspecies to the species level with later recombinations into *Oreomecon* by Krivenko (2023). Similar recombinations and status changes were done for P.lapponicumsubsp.jugoricum Tolm. and P.microcarpumsubsp.czekanowskii Tolm. by Krivenko (2023), whereas a status change for P.nudicaulesubsp.insulare V.V.Petrovsky was undertaken by Chepinoga et al. (2023). These species-level changes were done, based on the authors’ general non-acceptance of subspecific taxa and not by evaluating existing classifications in the *Papaver* literature, which is the preferred alternative here.

The Wrangel Island endemic *Papaveruschakovii* Tolm. & V.V.Petrovsky was accepted by Petrovsky (1999), Solstad et al. (2009), with molecular support and Elven et al. (2011) and is recombined into *Oreomecon* below. It was included in *Papaverpolare* Tolm. by Xue et al. (2023) and POWO (2023) and was not treated by Krivenko (2023). According to Elven et al. (2011), Papaveruschakoviisubsp.tichomirovii Kozhevn. from Chukotka, does not belong in *P.uschakovii*, nor in *P.dahlianum* s.lat. and may have affiliation with a still undescribed species.

A particular case concerns the species group with amphi-Beringian distributions, which comprise four species treated in the monograph sources. *Papaverdetritophilum* Petrovsky has most of its distribution area on the Russian side and is treated within this group of Asian-Arctic species. *Papaverkeelei* A.E.Pors., *P.gorodkovii* Tolm. & Petrovsky and *P.walpolei* A.E.Pors. have their major distribution ranges on the American side and are treated in the section on taxa from Arctic Alaska and Yukon; see below. We conclude that *Oreomecon* is represented by 14 species and six subspecies in Arctic Asia. *Oreomeconlapponica* and *O.nudicaulis* occur with separate subspecies in this area and their nominate subspecies are treated as taxa 3.6.6 and 3.1.13, respectively, below the areas where they occur.

#### ﻿Distribution

**Figure 3.**
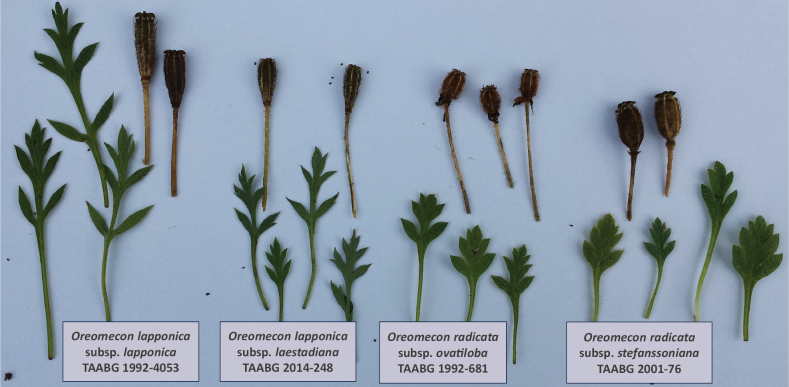
Specimens of Oreomeconlapponicasubsp.lapponica, O.lapponicasubsp.laestadiana, O.radicatasubsp.ovatiloba (1992-681) and O.radicatasubsp.stefanssoniana (2001-76) under comparative cultivation in Tromsø Arctic-Alpine Botanic Garden (TAABG).

The distribution map in Fig. 3 shows a gap in Central Siberia between groups A and C. The distribution maps by Peschkova (1994) only show a connection in easternmost Siberia. However, some occurrences are shown by GBIF (Global Biodiversity Information Facility) Secretariat (2023) along the Verkhoyansk Mountain Range and we map the connection here.

#### ﻿Rare and threatened species

The most exclusive ones in this group are the endemic taxa from Wrangel Island, which were treated by Petrovsky (1997, 1999) and mapped by Petrovsky in Talbot et al. (1999), each shown from 4 - 8 localities and protected in the Wrangel Island State Reserve. *Papavergorodkovii* Tolm. & Petrovsky was included by Petrovsky (1997), but, according to Petrovsky (1999), the disjunct and large Wrangel Island population of this mostly American species is polymorphic and insufficiently understood. The remaining five species and one subspecies mapped by Talbot et al. (1999) are endemic to Wrangel Island, except *Papaveratrovirens* V.V.Petrovsky also occurring on the adjacent mainland and *P.calcareum* V.V.Petrovsky also from north-eastern Chukchi Peninsula (Elven et al. 2011).

Xue et al. (2023) did not list any Endangered (EN) or Vulnerable (VU) species from the Asian Arctic, but treated seven taxa as Near Threatened (NT). These did not include the rare species from Wrangel Island, but instead widespread taxa such as *P.czekanowskii* and P.lapponicumsubsp.jugoricum (Tolm.) S.V.I. Gudoshn. They also accepted *P.indigirkense*, which we, however, consider a synonym of P.lapponicumsubsp.orientale.

#### ﻿Accepted taxa

##### ﻿3.3.1. Oreomecon angustifolia

Taxon classificationPlantaeRanunculalesPapaveraceae

(Tolm.) Krivenko, Nov. Syst. Pl. Vasc. 54: e06:1. 2023

E26C49F5-D356-51D9-8BD5-5D8849714263

≡ Papaverangustifolium Tolm., Trudy Bot. Muz. 22: 369. 1930. Type: [Russia] Siberia: Gydan Tundra, Obvalivayushchiesya beregovye sklony u NO vhodnogo mysa Yurackoy Guby, 15 Aug 1926, *A.I. Tolmachev 589* (lectotype: LE, lectotypified by Elven et al. [2009], p. 988).

##### ﻿3.3.2. Oreomecon anjuica

Taxon classificationPlantaeRanunculalesPapaveraceae

(Tolm.) Krivenko, Nov. Syst. Pl. Vasc. 54: e06:2. 2023

83974B62-A663-5690-B5C6-926E2780D7CA

≡ Papaveranjuicum Tolm., in V.L. Komarov (ed.) Arkt. Fl. SSSR 7: 25. 1975. Type: Russian Far East: West Chukotka, [in parte septentrionali montium Anjuicum, ad fontes fluminis Erguveem], 11 Jul 1967, *E. Zimarskaja, A. Korobkov & B. Yurtsev s.n.*, (holotype: LE).

##### ﻿3.3.3. Oreomecon atrovirens

Taxon classificationPlantaeRanunculalesPapaveraceae

(V.V.Petrovsky) Krivenko, Nov. Syst. Pl. Vasc. 54: e06:2. 2023

964328A4-E28A-5A60-AFA1-C95B1E50DD57

≡ Papaveratrovirens V.V.Petrovsky, Bot. Zhurn. (Moscow & Leningrad) 68: 231. 1983. Type: [Russia: Insula Wrangelii, ad litus meridionale, ad sinum Somnitelnaja], 16 Jul 1971, fl. et fr. immat., *V. Petrovsky 71-33* (holotype and isotypes: LE).

##### ﻿3.3.4. Oreomecon calcarea

Taxon classificationPlantaeRanunculalesPapaveraceae

(V.V.Petrovsky) Krivenko, Nov. Syst. Pl. Vasc. 54: e06:2. 2023

56615823-CFF7-5A43-BE79-FE9854692D2F

≡ Papavercalcareum V.V.Petrovsky, Bot. Zhurn. (Moscow & Leningrad) 68: 232. 1983. Type: [Russia: Insula Wrangelii, ad fl. Gussinaja, in declivibus glareosis calcareis], 14 Jul 1969, fl. et fr. immat., *V. Petrovsky s.n.* (holotype: LE).

##### ﻿3.3.5. Oreomecon chionophila

Taxon classificationPlantaeRanunculalesPapaveraceae

(V.V.Petrovsky) Krivenko, Nov. Syst. Pl. Vasc. 54: e06:2. 2023

79195353-78C5-58D3-9DAF-602D8FA0E25B

≡ Papaverchionophilum V.V.Petrovsky, Bot. Zhurn. (Moscow & Leningrad) 68: 233. 1983. Type: [Russia: Insula Wrangelii, ad sinum Somnitelnaya, ad fl. Somnitelnaja, alluvium], 5 Aug 1971, fl., *V. Petrovsky 71-357* (holotype and isotypes: LE).

##### ﻿3.3.6. Oreomecon czekanowskii

Taxon classificationPlantaeRanunculalesPapaveraceae

(Tolm.) Krivenko, Nov. Syst. Pl. Vasc. 54: e06:2. 2023

C4E028DD-4141-515B-A678-5913F209A1F1

≡ Papaverczekanowskii Tolm., Bot. Mater. Gerb. Bot. Inst. Komarova Akad. Nauk S.S.S.R 20: 172. 1960. Type: [Russia: Siberia, Yakutia, ad brachium delta Lenae fluminis Olenekskaja protoka dictum, ad pagum Czaj-Tumus], 19 Jul 1956, *A. Tolmatchev* (holotype: LE) ≡ Papavermicrocarpumsubsp.czekanowskii (Tolm.) Tolm., Fl. Arct. URSS 7: 31. 1975.

##### ﻿3.3.7. Oreomecon detritophila

Taxon classificationPlantaeRanunculalesPapaveraceae

(V.V.Petrovsky) Krivenko, Nov. Syst. Pl. Vasc. 54: e06:2. 2023

B790D3EB-AF81-57B9-84D6-0B80326070DE

≡ Papaverdetritophilum V.V.Petrovsky, Bot. Zhurn. (Moscow & Leningrad) 70: 114. 1985. Type: Russian Far East: West Chukotka, [jugum Anjujensis, in systemate fl. Anjuj Magnus, in valle fl. Bystrjanka], 25 Jul 1980, *V.V.Petrovsky 80-59* (holotype: LE).

##### ﻿3.3.8. Oreomecon hypsipetes

Taxon classificationPlantaeRanunculalesPapaveraceae

(V.V.Petrovsky) Krivenko, Nov. Syst. Pl. Vasc. 54: e06:2. 2023

A47422FF-F8F0-5ABF-8388-0CEADC49AF1A

≡ Papaverhypsipetes V.V.Petrovsky, Bot. Zhurn. (Moscow & Leningrad) 70: 113. 1985. Type: Russian Far East: West Chukotka, [districtus Bilibinskij, montes Anjujensis, jugum Ilirnejensis, ad lacus Ilirnej Superior], 18 Jul 1973, *V.V.Petrovsky 73-26* (holotype: LE).

##### ﻿3.3.9. Oreomecon lapponica subsp. jugorica

Taxon classificationPlantaeRanunculalesPapaveraceae

(Tolm.) Elvebakk & Bjerke nomencl. nov.

E3E8124A-732A-5421-B96A-CD4DE9144D23

urn:lsid:ipni.org:names:77350925-1

≡ Papaverradicatumsubsp.jugoricum Tolm., Bot. Mater. Gerb. Inst. Komarova Akad. Nauk S.S.S.R. 4:86. 1923. Type: [northern European Russia] Ostrov Vaigach, sev. podereshchie, bukhta Varneka, na otmeli, 2 Sep 1921, *A. Tolmatchev 352* (lectotype: LE, designated by Egorova [1998] p. 99) ≡ Papaverlapponicumsubsp.jugoricum (Tolm.) Gudoschn., Fl. Krasnoy. Kraya 5 (4): 6. 1975 ≡ Oreomeconjugorica (Tolm.) Krivenko, Nov. Syst. Pl. Vasc. 54: e06:2. 2023.

##### ﻿3.3.10. Oreomecon lapponica subsp. orientalis

Taxon classificationPlantaeRanunculalesPapaveraceae

(Tolm.) Elvebakk & Bjerke nomencl. nov.

1F0F5086-6B8F-5430-8F6D-4949BCF448C4

urn:lsid:ipni.org:names:77350926-1

≡ Papaverlapponicumsubsp.orientale Tolm., Trudy Polyarn. Komiss. 13: 131. 1932. Type: Siberia: Vostochniy Taimyr. Nizoviya r. Yamu-Neri (bassein Taimyrskogo ozera), raion letoviya ekspeditsii (74°50'N, 106°E), 5 Aug 1928, *A.I. Tolmachev 582* (lectotype: LE, lectotype designated by Elven et al. [2009: 987]) ≡ Oreomeconorientalis (Tolm.) Krivenko, Nov. Syst. Pl. Vasc. 54: e06:2. 2023 ≡ Papaverpospelovae Barkalov & Chepinoga, Botanica Pacifica 12, 2: 124. 2023, nom. nov., non P.orientale L. 1753.
= Papaverminutiflorum Tolm., Bot. Mater. Gerb. Bot. Inst. Komarova Akad. Nauk S.S.S.R. 20: 180. 1960; Type: Russia, E Siberia, Yakutskaya ASSR, Tomponskii rayon. Bassein r. Tompo r. Seyule, 5 Aug. 1956, *I.D.Kildjushevskii 30/1*, (LE, lectotype, selected by Elven et al. [2009: 987]) ≡ Oreomeconminutiflora (Tolm.) Krivenko, Nov. Syst. Pl. Vasc. 54: e06:2. 2023.
= Papaverindigirkense Jurtzev, Novosti Sist. Vyssh. Rast. 2: 310. 1965. Type: [Russia] Jacutia orientalis, in parte superiore fl. Indigirka, ad fl. Chugutjan, in declivi substepposo, 1 Jul 1958, *B.A. Jurtzev s.n*. (LE, holotype).

##### ﻿3.3.11. Oreomecon multiradiata

Taxon classificationPlantaeRanunculalesPapaveraceae

(V.V.Petrovsky) Krivenko, Nov. Syst. Pl. Vasc. 54: e06:2. 2023

FAF51EEE-B58D-57F4-B2F6-FA78B0ACD44D

≡ Papavermultiradiatum V.V.Petrovsky, Bot. Zhurn. (Moscow & Leningrad) 68: 284. 1983. Type: Russian Far East: Wrangel Island, [ad sinum Somnitelnaja], 23 Jul 1965, *V.V.Petrovsky* (holotype: LE).

##### ﻿3.3.12. Oreomecon nudicaulis subsp. insularis

Taxon classificationPlantaeRanunculalesPapaveraceae

(V.V.Petrovsky) Elvebakk & Bjerke comb. nov.

CD7BCA39-7D24-54AE-82D6-F5DDB976F7FD

urn:lsid:ipni.org:names:77350927-1

≡ Papavernudicaulesubsp.insulare V.V.Petrovsky, Bot. Zhurn. 68: 236. 1983. Type: Russian Far East: Wrangel Island, [ad sinum Somnitelnaja], 10 Aug 1979, *V.V.Petrovsky 79-96* (holotype: LE) ≡ Papaverinsulare (V.V.Petrovsky) Barkalov & Chepinoga, Botanica Pacifica 12, 2: 124. 2023.

##### ﻿3.3.13. Oreomecon paucistamina

Taxon classificationPlantaeRanunculalesPapaveraceae

(Tolm. & V.V.Petrovsky) Krivenko, Nov. Syst. Pl. Vasc. 54: e06:3. 2023

1B23C95F-539A-5754-BF22-9636D4FD13E0

≡ Papaverpaucistaminum Tolm. & V.V.Petrovsky, Bot. Zhurn. (Moscow & Leningrad) 58: 1129. 1973. Type: Russian Far East: West Chukotka, [in montibus partis centralis Terrae Tschuktschorum, in ditione fluminis Quëkvun], 26 Jul 1966, *V.V.Petrovsky s.n.* (holotype: LE).

##### ﻿3.3.14. Oreomecon pulvinata (Tolm.) Krivenko subsp. pulvinata

Taxon classificationPlantaeRanunculalesPapaveraceae

, Nov. Syst. Pl. Vasc. 54: e06:2. 2023

95AAE4AF-7A4B-5911-90D1-5BE18829042C

≡ Papaverpulvinatum Tolm., Trudy Bot. Muz. 24: 269. 1932. Type: [Russia]: Siberia, Taimyr, Nizovya r. Yamu-Tarida (bassein Taimyrskogo ozera), raion vesnovki ekspeditsii (74°27'N, 102°50'E), 5 Aug 1928, *A.I. Tolmachev 135* (lectotype: LE, lectotypified by Elven et al. [2009] p. 988).

##### ﻿3.3.15. Oreomecon pulvinata subsp. alexandri

Taxon classificationPlantaeRanunculalesPapaveraceae

(V.V.Petrovsky) Elvebakk & Bjerke nomencl. nov.

68246F4F-81D1-55B6-AC8C-740DBC4212EB

urn:lsid:ipni.org:names:77350928-1

≡ Papaverpulvinatumsubsp.alexandri V.V.Petrovsky, Ann. Bot. Fenn. 56: 371. 2019. Type: Russia. [NE Siberia] Yakut ASSR, N end of Kharaulakhskii Range, right bank of Bykovskaya branch (delta of the Lena River), environs of Sokol settlement (72°20'N, 125°40'E), Kiries-Khamo Bay, sandy terrace, 19 Aug 1956 *T.G. Polozova, B.A. Yurtsev s.n* (holotype: LE [barcode LE 01026076], fig. 2, illustrated by Petrovsky et al. [2019]: 372; isotypes: LE [barcode LE 01026077], LE [barcode LE 01026078]) ≡ Papaveralexandri (V.V.Petrovsky) Barkalov & Chepinoga, Botanica Pacifica 12, 2: 124. 2023 ≡ Oreomeconalexandri (V.V.Petrovsky) Krivenko, Nov. Syst. Pl. Vasc. 54: e06:1. 2023 ≡ Papaverpulvinatumsubsp.lenaense Tolm., Arkt. Fl. SSSR 7: 24. 1975, nom. illeg.

##### ﻿3.3.16. Oreomecon pulvinata subsp. interior

Taxon classificationPlantaeRanunculalesPapaveraceae

(V.V.Petrovsky) Elvebakk & Bjerke nomencl. nov.

0FF4C421-C7A1-51C0-960B-6D89B64CBA82

urn:lsid:ipni.org:names:77350929-1

≡ Papaverpulvinatumsubsp.interius V.V.Petrovsky, Bot. Zhurn. (Moscow & Leningrad) 65: 657. 1980) Type: Russia. West Chukotka, Anyui Upland, northern part, middle reaches of the Kytep-Guiten’ryveem River, Baraniy brook ravine, alluvial fan of a tributary, 17 Aug 1977 *V. Petrovsky 77-44P* (holotype: LE [barcode LE 01035172]; isotype: LE [barcode LE 01035173]) ≡ Papaverinterius (V.V.Petrovsky) Barkalov & Chepinoga, Botanica Pacifica 12, 2: 124. 2023 ≡ Oreomeconinterior (V.V.Petrovsky) Krivenko, Nov. Syst. Pl. Vasc. 54: e06:2. 2023, “interius”.

##### ﻿3.3.17. Oreomecon pulvinata subsp. tschuktschorum

Taxon classificationPlantaeRanunculalesPapaveraceae

(Tolm.) Elvebakk & Bjerke nomencl. nov.

B4C78436-8458-516F-ADA2-6C59E85C734F

urn:lsid:ipni.org:names:77350930-1

≡ Papaverpulvinatumsubsp.tschuktschorum Tolm., Arkt. Fl. SSSR 7: 24. 1975. Type: N. Russian Far East, Ostrov Vrangelya, buchta Somnitelnaya, r. Somnitelnaya, 20 Jul 1964, *V.V.Petrovsky s.n*. (lectotype: LE [barcode LE 01042299], designated by Elven et al. [2009: 988]; illustrated by Petrovsky et al. [2019] p. 374) ≡ Oreomecontschuktschorum (Tolm.) Krivenko, Nov. Syst. Pl. Vasc. 54: e06:3. 2023.

##### ﻿3.3.18. Oreomecon schamurinii

Taxon classificationPlantaeRanunculalesPapaveraceae

(V.V.Petrovsky) Krivenko, Nov. Syst. Pl. Vasc. 54: e06:3. 2023

19A56235-743D-5264-A9B2-30E442ED6E14

≡ Papaverschamurinii V.V.Petrovsky, Bot. Zhurn. (Moscow & Leningrad) 70: 116. 1985. Type: Russian Far East: Wrangel Island, [sinus Somnitelnaja, ad litus lacunae Bazovaja], 12 Jul 1971, *V.V.Petrovsky 71-200* (holotype: LE).

##### ﻿3.3.19. Oreomecon uschakovii

Taxon classificationPlantaeRanunculalesPapaveraceae

(Tolm. & V.V.Petrovsky) Elvebakk & Bjerke comb. nov.

CD24E35A-08BD-5744-B946-FB1DE57C97B0

urn:lsid:ipni.org:names:77350931-1

≡ Papaveruschakovii Tolm. & V.V.Petrovsky, Bot. Zhurn. 58: 1128. 1973. Type: Russian Far East: Wrangel Island, [ad sinum Rogersii], 27 Jun 1969, *V.V.Petrovsky s.n*. (holotype: LE).

##### ﻿3.3.20. Oreomecon variegata

Taxon classificationPlantaeRanunculalesPapaveraceae

(Tolm.) Krivenko, Nov. Syst. Pl. Vasc. 54: e06:3. 2023

7A0D85F4-5FC3-5D54-A83B-C97F550565DA

≡ Papavervariegatum Tolm., in V.L. Komarov (ed.) Arkt. Fl. SSSR 7: 20. 1975. Type: Siberia: Putorana, [ad litus meridionalis lacu Khantaika, prope ostium fluminis Mogaddy], 7 Aug 1970, *A. Tolmachev* (holotype: LE).

### ﻿3.4. Arctic Alaska and Yukon and adjacent Cordilleras

#### ﻿Notes

Most of the taxa of Papaversect.Meconella in this area are shared between the Arctic parts of Alaska and Yukon as defined by Walker et al. (2005) and the northernmost part of the North American Cordilleras, whereas other species predominate further south (Kiger and Murray 1997; Björk 2019; GBIF (Global Biodiversity Information Facility) Secretariat 2023). Distribution area D is, therefore, defined as Arctic Alaska and Yukon and adjacent parts of the northern North American Cordilleras delimited southwards by the border with British Columbia and eastwards by the Mackenzie River. The primary source for area D is Elven et al. (2011), where seven species centred in Arctic Alaska were included. Only three of these species were accepted under the same names by “Flora of North America” (Kiger and Murray 1997).

The list below includes amphi-Beringian species, including *P.walpolei* A.E.Porsild, which occurs in eastern Chukotka and *P.gorodkovii* Tolm. & V.V.Petrovsky, which also extends across the Bering Strait to Wrangel Island. According to Elven et al. (2011), *P.keelei* A.E.Porsild is by far the most common species in the Arctic part of the area, ranging from Chukotka to the western mainland of the Northwest Territories. It was not treated by Galasso et al. (2023), Grey-Wilson (2023) or Krivenko (2023) and is recombined into *Oreomecon* below. *Papaverhultenii* Knaben is common in Alaska and also occurs eastwards to Nunavut, but its presence in Chukotka is uncertain, according to Elven et al. (2011). None of these species occurs south of area D, except *P.hultenii*, known from the Pink Mt. area in British Columbia (Björk 2019). Björk (2019) reported that *P.roseoalbum* Björk is mainly from south-central Alaska, with a few collections outside area D in the far north-western part of British Columbia.

Neither Björk (2019) nor Krivenko (2023) referred to P.nudicaulesubsp.americanum Rändel ex D.F.Murray, which was described by Rändel (1977) and validated by Murray (1995). The taxon was studied by morphology and AFLP by Solstad et al. (2009). They concluded that it was hexaploid as opposed to the typical forms of *P.nudicaule* and that it was the only American representative from southern and eastern Alaska and Yukon of a large complex including this species. It was also accepted by Elven et al. (2011), who did not recognise it from the Arctic parts of North America. In contrast, Kiger and Murray (1997) did not discriminate between this taxon and introduced “Iceland poppies” (*P.croceum*). The nominate subspecies of *O.nudicaulis* is treated here as taxon 3.1.13.

Krivenko (2023) omitted the author name in the citation of the basionym in his recombination of *Oreomeconalaskanum* (Hultén) Krivenko. Following the Code (Art 41.6; Turland et al. (2018)), this is considered to be within the range of allowed erroneous basionym citations. Krivenko (2023) also transferred *Papaverdenalii* Gjærevoll, a species previously accepted by Björk (2019), to *Oreomecon*. However, Solstad et al. (2009) and Elven et al. (2011) considered it a synonym of *P.mcconnellii* Hultén. Galasso et al. (2023) treated P.macouniivar.discolor Hultén as a subspecies of *Oreomecon*, while Krivenko (2023) raised it to species level. In contrast, Solstad et al. (2009) and Elven et al. (2011) considered it a synonym of *P.keelei*. The latter authors are followed here in both cases.

*Papavermacounii* Greene is probably the rarest species in this area, known from only a few scattered sites (Elven et al. 2011). *Papaveralaskanum* Hultén is also a rare species, but was considered heterogeneous by Elven et al. (2011). This heterogeneity has not yet been resolved. Cortés-Burns et al. (2009) included *Papavergorodkovii* and mapped its distribution in their treatment of the rare plants of the Alaskan North Slope. However, there is no coordinated evaluation of the conservation status of Papaversect.Meconella in North America. A total of eight species and one subspecies are listed below. *Papavermcconnellii* was listed as VU and *P.walpolei* as NT in Russia by Xue et al. (2023).

#### ﻿Accepted taxa

##### ﻿3.4.1. Oreomecon alaskana

Taxon classificationPlantaeRanunculalesPapaveraceae

(Hultén) Krivenko, Nov. Syst. Pl. Vasc. 54: e06:1. 2023

975CDD60-8ED9-517D-AFE6-71A9D7151A30

≡ Papaveralaskanum Hultén, Fl. Aleutian Isl.: 190. 1937. Type: [USA] Alaska: the Aleutian Islands, Unalaska, 2 Aug 1932, *E. Hultén 7197* (holotype: S [no. S-G-4519] ≡ Papaverradicatumsubsp.alaskanum (Hultén) J.P. Anderson, Fl. Alaska: 244. 1959 ≡ Papavermicrocarpumsubsp.alaskanum (Hultén) Tolm., Fl. Arct. URSS 7: 30. 1975.

##### ﻿3.4.2. Oreomecon gorodkovii

Taxon classificationPlantaeRanunculalesPapaveraceae

(Tolm. & V.V.Petrovsky) Krivenko, Nov. Syst. Pl. Vasc. 54: e06:2. 2023

E636A585-5C6C-5F10-B2C4-45D9791D3847

≡ Papavergorodkovii Tolm. & V.V.Petrovsky, Bot. Zhurn. (Moscow & Leningrad) 58: 1128. 1973. Type: Russian Far East: Wrangel Island, [ad sinum Somnitelnaja], 24 Jul 1971, V.V.Petrovsky & N. Taraskina s.n. (holotype: LE).

##### ﻿3.4.3. Oreomecon hultenii

Taxon classificationPlantaeRanunculalesPapaveraceae

(Knaben) Krivenko, Nov. Syst. Pl. Vasc. 54: e06:2. 2023

32A1F743-289A-580C-830F-308876A1A60D

≡ Papaverhultenii Knaben, Opera Bot. 3(3): 49. 1959. Type: Canada: Nunavut, Copper Mine River, *M. Hammer*, plants cultivated in Oslo from seeds collected in 1948 (holotype: O [barcode O-V-2014581]).

##### ﻿3.4.4. Oreomecon keelei

Taxon classificationPlantaeRanunculalesPapaveraceae

(A.E.Porsild) Elvebakk & Bjerke comb. nov.

64288D94-7712-5749-92C2-C3150EC7774F

urn:lsid:ipni.org:names:77350932-1

≡ Papaverkeelei A.E.Porsild, Bull. Natl. Mus. Canada 101: 20. 1945. Type: Canada: the Yukon Territory, Canal Road, Mackenzie Range, small tributary to Little Keele River, Mile 51, 8 Sep 1944, *A.E.Porsild and A.J. Breitung 11,782* (holotype: CAN; isotype S [no. S-G-4528]).
= Papavermacouniivar.discolor Hultén, Acta Univ. Lund., n. s., sect. 2, 41, 1: 803. 1945. Type: Alaska: Seward Peninsula, Nome, hillside, 11 Jul 1938, *J.P. Anderson 3250* (holotype S) ≡ Papavermacouniisubsp.discolor (Hultén) Rändel ex D.F.Murray, Novon 5: 294. 1995
≡ Oreomeconmacouniisubsp.discolor (Hultén) Galasso, Banfi & Bertolucci, Pl. Rev. 5(4): 58. 2023 ≡ Oreomecondiscolor (Hultén) Krivenko, Nov. Syst. Pl. Vasc. 54: e06:2. 2023.

##### ﻿3.4.5. Oreomecon macounii

Taxon classificationPlantaeRanunculalesPapaveraceae

(Greene) Galasso, Banfi & Bertolucci, Pl. Rev. 5(4): 58. 2023

0CEA3D57-96E8-5A2E-BE5F-8A949C8DE89F

≡ Papavermacounii Greene, Pittonia 3: 247. 1897. Type: Alaska: the Pribilof Islands, St. Paul Island, Jun-Jul 1897, *J.M. Macoun s.n.* (CAN, C [barcode C10016380]; K [barcode K 000653200]; NDG [barcode NDG20296].

##### ﻿3.4.6. Oreomecon mcconnellii

Taxon classificationPlantaeRanunculalesPapaveraceae

(Hultén) Krivenko, Nov. Syst. Pl. Vasc. 54: e06:2. 2023

332C76FC-9341-5611-A954-BD7E8119A364

≡ Papavermcconnellii Hultén, Acta Univ. Lund. 2, 41(1): 803. 1945. Type: Canada: Northwest Territories, mountains between Peel River and La Pierre House, 1 Jul 1888, *McConnell s.n.* (holotype in unknown herbarium; photograph and fragment of holotype at S [no. S-G-4530]).
= Papaverdenalii Gjærev., Kongel. Norske Vidensk. Selsk. Skr. (Trondheim) 1963: 4:42. 1964. Type: Alaska, the Alaska Range, McKinley Park, Cathedral Mountain, 20 Jul 1959, *O. Gjærevoll* (holotype: TRH) ≡ Oreomecondenalii (Gjærev.) Krivenko, Nov. Syst. Pl. Vasc. 54: e06:2. 2023.

##### ﻿3.4.7. Oreomecon nudicaulis subsp. americana

Taxon classificationPlantaeRanunculalesPapaveraceae

(Rändel ex D.F.Murray) Elvebakk & Bjerke comb. nov.

C3DADFF0-CCF5-5734-A7C3-23E14646E4EC

urn:lsid:ipni.org:names:77350933-1

≡ Papavernudicaulesubsp.americanum Rändel ex D.F.Murray, Novon 5: 295. 1995. Type: Canada: the Yukon Territory, the Klotassin area, southwest of Yukon River, between Selkirk and White River, Aug 1916, *D.D. Cairnes 91890* (holotype: CAN).

##### ﻿3.4.8. Oreomecon roseoalba

Taxon classificationPlantaeRanunculalesPapaveraceae

(Björk) Krivenko, Nov. Syst. Pl. Vasc. 54: e06:2. 2023

C626CC0E-EFF0-504D-92B2-E082537F30B5

≡ Papaverroseoalbum Björk, Phytoneuron 2019-6: 10. 2019. Type: USA: Alaska. Populus-Alnus thicket, at mouth of small canyon, W of Portage Glacier, 12 Jul 1968, *Welsh 8146* (holotype: ALA [barcode ALA274045]).

##### ﻿3.4.9. Oreomecon walpolei

Taxon classificationPlantaeRanunculalesPapaveraceae

(A.E.Porsild) Krivenko, Nov. Syst. Pl. Vasc. 54: e06:3. 2023

1EE41A18-8F21-5492-A984-293211F5080A

≡ Papaverwalpolei A.E.Porsild; Rhodora 41: 231 (1939). Type: Alaska: Seward Peninsula, Anvil Hill, *A.E.Porsild 1352* (holotype: CAN).

### ﻿3.5. North American Cordilleras

#### ﻿Notes

The delimitation northwards of area E is the northern boundary of British Columbia. The reference study for this area is Björk (2019), primarily focusing on British Columbia, but also dealing with material from other parts and making significant changes to the taxonomy of the group. *Papavercolumbianum* Fedde ex Björk and *P.kluanense* D.Löve only extend into adjacent areas of southern Yukon, according to Björk (2019), who also showed that most of the plants from the US part of the Rocky Mountains represent *Papavercoloradense* (Fedde) Fedde ex Wooton & Standley. The central part of the North American Cordilleras now includes five species.

#### ﻿Distribution

The distribution map in Fig. 2 also integrated distribution maps presented by Kiger and Murray (1997).

#### ﻿Rare and protected species

Björk (2019) stated that both *P.columbianum* Fedde ex Björk and *P.luculentum* Björk may be rare. The former is only known from four collections. However, they are so far apart that the occurrence of undiscovered localities was cited to be likely.

#### ﻿Accepted taxa

##### ﻿3.5.1. Oreomecon coloradensis

Taxon classificationPlantaeRanunculalesPapaveraceae

(Fedde) Krivenko, Nov. Syst. Pl. Vasc. 54: e06:2. 2023

8ECDC4F7-341F-5F2D-81C9-DA1C0235D7F6

≡ Papavernudicaulevar.coloradense Fedde, Repert. Spec. Nov. Regni Veg. 7: 256. 1909. Type: USA: Colorado. East of Middle Park, 1867, *Parry 147* (lectotype: BM [barcode BM574948], designated by Björk [2019], p. 17) ≡ Papavercoloradense (Fedde) Fedde ex Wooton & Standl., Contr. U.S. Natl. Herb. 19: 262. 1915.

##### ﻿3.5.2. Oreomecon columbiana

Taxon classificationPlantaeRanunculalesPapaveraceae

(Fedde ex Björk) Krivenko, Nov. Syst. Pl. Vasc. 54: e06:2. 2023

AFFDC3DC-3A72-52C3-9BE8-CE60D7ED3B90

≡ Papavercolumbianum Fedde ex Björk, Phytoneuron 2019-6: 5. 2019. Type: Canada: British Columbia, mountains at Kicking [Horse], [possibly Yoho National Park], 8000 ft, 14 Aug 1890, *Macoun s.n.* (holotype: US, no. 99717) ≡ Papavernudicaulevar.columbianum Fedde; Repert. Spec. Nov. Regni Veg. 7: 255 (1909), nom. illeg.

##### ﻿3.5.3. Oreomecon kluanensis

Taxon classificationPlantaeRanunculalesPapaveraceae

(D.Löve) Galasso, Banfi & Bertolucci, Pl. Rev. 5(4): 58. 2023

E84D0924-B3C7-55EB-8CAC-F7AC86E5F5A5

≡ Papaverkluanense D.Löve, Bot. Not. 109: 178. 1956. Type: Canada: Yukon Territory. North of Quill Creek Camp, alt. ca. 5000 ft, 20 mi W of Burwash Landing, 15 Jun 1953, *Freedman s.n.* (holotype: MAN; fragment and photograph of holotype at S [no. S S08-261]) ≡ Papaverradicatumsubsp.kluanense (D.Löve) D.F.Murray, Novon 5: 294. 1995.

##### ﻿3.5.4. Oreomecon luculenta

Taxon classificationPlantaeRanunculalesPapaveraceae

(Björk) Krivenko, Nov. Syst. Pl. Vasc. 54: e06:2. 2023

95C0A11D-3FBE-5565-906C-A988FF3F681D

≡ Papaverluculentum Björk, Phytoneuron 2019–6: 7. 2019. Type: Canada: British Columbia. Boundary Ranges, ridge N of North Treaty Creek, near Bowser Lake, W of Bell Irving River, 56°38'6.88"N, 129°52'13.18"W, on fine argillite gravel scree, windblown alpine ridge, 4 Jul 2013, *Björk 32373* (holotype: UBC).

##### ﻿3.5.5. Oreomecon pygmaea

Taxon classificationPlantaeRanunculalesPapaveraceae

(Rydb.) Krivenko, Nov. Syst. Pl. Vasc. 54: e06:2. 2023

8C952994-9298-527C-BBFE-62486C87FD7B

≡ Papaverpygmaeum Rydb., Bull. Torrey Bot. Club 29: 159. 1902. Type: USA: Montana. Mountain above Stanton Lake, 1 Aug 1894, *Williams 992* (lectotype: NY [NY99719], designated by Björk [2019], p. 2).

### ﻿3.6. Central and eastern Arctic Canada, Greenland and Arctic Europe

#### ﻿Notes

The taxa present in the Canadian Arctic Archipelago were treated by Solstad (2007). However, the reference study for this area, where two widely distributed High-Arctic taxa are characteristic, is Elven et al. (2011). One of these taxa is *Papavercornwallisense* D.Löve, while the other is Papaverdahlianumsubsp.polare (Tolm.) Elven & Ö.Nilsson. Solstad et al. (2014) reported both to be very common in Svalbard and equally widespread in Arctic North America. Elven et al. (2011) had left the basionym of Papaverdahlianumsubsp.polare unassigned, as its type housed at LE is from Svalbard and could potentially represent *P.cornwallisense*, a more recently described taxon. However, Solstad et al. (2014) confirmed that the type represents *P.dahlianum* s.lat. Hence, *P.cornwallisense* remains the name of this distinct taxon. It was not treated in any recent *Oreomecon* studies and is, therefore, transferred to *Oreomecon* here.

Russian authors have traditionally treated *P.polare* Tolm. as distinct at the species level and this view was recently maintained by Xue et al. (2023), POWO (2023) and Krivenko (2023), the latter recombining it into *Oreomecon*. However, all recent studies comparing this taxon with *P.dahlianum* Nordh., partly with molecular support (Nilsson 2001; Solstad et al. 2003; 2009; 2014; Elven et al. 2011), conclude that these taxa are conspecific and that the older name *P.dahlianum* holds priority. Nilsson (2001) divided Papaverdahlianuminto a widespread High-Arcticsubsp.polareand a Low-Arcticsubsp.dahlianum Nordh. Papaverlapponicumsubsp.dasycarpum Tolm., recorded from Novaya Zemlya by Tolmachev (1975), was tentatively treated within *P.dahlianum* by Elven et al. (2011) and an interpretation within subsp. *polare* is followed here.

The latter subspecies occurs in the southernmost part of the Arctic in Finnmark, Norway and adjacent mountains in the Kola Peninsula, where it was described as *P.lujaurense* N.Semenova (Semenova-Tian-Shanskaya 1956). Based on an AFLP-based molecular analysis, Solstad et al. (2009) rejected the segregation into two distinct subspecies and this view on *Papaverdahlianum* was shared by Elven et al. (2011). However, Elven et al. (2022) indicated this might not be a final conclusion given the existing morphological differences and the genetic markers used. They also indicated that P.dahlianumssp.polare has recently been discovered in the southeastern part of the Municipality of Porsanger/Porsáŋggu/Porsangin in Finnmark, ca. 100 km S of the polar treeline. Recently, J.O. Olsen and others posted on Artsdatabanken (2024) a number of additional localities of *Papaverdahlianum* from a small area in the Municipality of Gáivuotna/Kåfjord/Kaivuonu another 200 km further to the southwest. As further studies of this complex continue, we maintain Nilsson’s interpretation (2001), which involves the acceptance of two separate subspecies of *P.dahlianum*.

Papaverlapponicumsubsp.occidentale (C.E.Lundstr.) Knaben occurs in Canada and Greenland, whereas P.lapponicumsubsp.lapponicum occurs from east Greenland eastwards to the westernmost parts of Siberia (Elven et al. 2011). The latter taxon was described from mountains just south of the Arctic border in the Kola Peninsula and two species described by Semenova-Tian-Shanskaya (1956) are considered synonyms. Papaverlapponicumsubsp.lapponicum also occurs in the Municipalities Alta and Kvænangen in North Norway, ca. 80 km S of the Arctic tree line as defined by Elvebakk and Karlsen (2022). These populations have been treated as separate subspecies, but are included within subsp. *lapponicum* by recent authors, including Elven et al. (2022). Here, they are considered peripheral populations of an Arctic taxon and are not included amongst taxa listed from non-Arctic northern Europe.

*Papaverlabradoricum* (Fedde) Solstad & Elven from Canada and Greenland was recombined at the species level by Elven and Murray (2008). The present treatment thus includes four species and two subspecies from this area and the only taxon restricted to the vast Arctic European area is P.dahlianumsubsp.dahlianum.

#### ﻿Distribution

The distribution shown in Fig. 2 includes all of Arctic Canada, Greenland and the European Arctic.

#### ﻿Rare and red-listed species

*Papaverdahlianum* s.lat. and *P.lapponicum* are both endangered (EN) in mainland Norway (Artsdatabanken 2021). Papaverlapponicumsubsp.lapponicum is Red-listed at the regional and national level in Russia and its populations on the Khibiny and Lovozerskie Mountains in the Kola Peninsula are protected within nature monuments as shown by Andreeva and Uotila (1998), where *P.lujaurense* was included within *P.lapponicum*.

##### ﻿3.6.1. Oreomecon cornwallisensis

Taxon classificationPlantaeRanunculalesPapaveraceae

(D.Löve) Elvebakk & Bjerke comb. nov.

0DFD8644-2DEF-55CB-8255-DA39F044DD50

urn:lsid:ipni.org:names:77350934-1

≡ Papavercornwallisense D.Löve, Bot. Not. 109: 176. 1956. Type: Canada: Nunavut, ex Insula Cornwallis, in Archipelago Arctico Americae, 31 Jul 1954, *J. Ritchie 663* (holotype: WIN).

##### ﻿3.6.2. Oreomecon dahliana

Taxon classificationPlantaeRanunculalesPapaveraceae

(Nordh.) Galasso, Banfi & Bertolucci, Pl. Rev. 5(4): 58. 2023

4CD0789E-03AF-5C91-AA6A-8A169B2347D3

≡ Papaverdahlianum Nordh., Bergens Mus. Årb. 1931, Naturvidensk. Rekke 2: 46. 1932. Type: Norway: Båtsfjord, Syltefjorden, Østerelven, på grus, 4 Jul 1930, *R. Nordhagen s.n.* (lectotype: O [barcode O-V-2014577], lectotype designated by Elven & Nilsson in Jonsell [2001] p. 521) ≡ Papaverradicatumsubsp.dahlianum (Nordh.) Rändel; Feddes Repert. 84: 694 (1974).
= Papaverlujaurense N.Semenova, Fl. Murmansk. Obl. 3: 369. 1956. Type: [Russia] Peninsula Kola, in montibus Lovoserskye-Tundry, prope pag. Revda, 25 Aug 1955, *N. Semenova-Tian-Shanskaya 185* (holotype: LE).

##### ﻿3.6.3. Oreomecon dahliana subsp. dahliana

Taxon classificationPlantaeRanunculalesPapaveraceae

(Nordh.) Galasso, Banfi & Bertolucci, Pl. Rev. 5(4): 58. 2023

54C80895-C0BB-5742-A74B-D72922D26107

≡ Papaverdahlianum Nordh., Bergens Mus. Årb. 1931, Naturvidensk. Rekke 2: 46. 1932. Type: Norway: Båtsfjord, Syltefjorden, Østerelven, på grus, 4 Jul 1930, *R. Nordhagen s.n.* (lectotype: O [barcode O-V-2014577], lectotype designated by Elven & Nilsson in Jonsell [2001] p. 521).

##### ﻿3.6.4. Oreomecon dahliana subsp. polaris

Taxon classificationPlantaeRanunculalesPapaveraceae

(Tolm.) Elvebakk & Bjerke nomencl. nov.

554AE533-718D-5062-9057-09BB2F1D86AF

urn:lsid:ipni.org:names:77350935-1

≡ Papaverradicatumsubsp.polare Tolm., Bot. Mater, Gerb. Glavn. Bot. Sada RSFSR 4: 87. 1923. Type: Norway: Svalbard, Advent Bay, 5–30 Jul 1898, *Semenkevich* (lectotype: LE, lectotype designated by Egorova [1998], p. 101) ≡ Papaverpolare (Tolm.) Perfil., in S.S. Stankov & V.I. Taliev, Syst. Classif. Vasc. Pl. Eur. Russ. 133. 1949 ≡ Papaverdahlianumsubsp.polare (Tolm.) Elven & Ö.Nilsson, Nordic J. Bot. 20: 522. 2001 ≡ Oreomeconpolaris (Tolm.) Krivenko, Nov. Syst. Pl. Vasc. 54: e06:3. 2023.
= Papaverlapponicumsubsp.dasycarpum Tolm., Trudy Bot. Muz. 25: 101. 1932. Not lectotypified, syntypes were listed from Matotschkin Schar in Novaya Zemlya by Egorova (1998).

##### ﻿3.6.5. Oreomecon labradorica

Taxon classificationPlantaeRanunculalesPapaveraceae

(Fedde) Krivenko, Nov. Syst. Pl. Vasc. 54: e06:2. 2023

6C4631C5-8073-5B93-B9CC-E073C61D0CE3

≡ Papavernudicaulevar.labradoricum Fedde, H.G.A. Engler (ed.) Pflanzenr., IV, 104: 377. 1909. Type: Greenland: Flora Groenlandiae boreali-occidentalis. Gebiet des Umanakfjords (70–71 N. Br.), 9 Aug 1893, *E. Vanhöffen 35(94)* (lectotype: B [barcode B10 0267999], lectotype designated by Elven et al. (2009) p. 986) ≡ Papaverradicatumsubsp.labradoricum (Fedde) Fedde, in Engl. & Prantl, Nat. Pflanzenfam., ed. 2, 17b: 120. 1936 ≡ Papaverlabradoricum (Fedde) Solstad & Elven, J. Bot. Res. Inst. Texas 2: 438. 2008 ≡ Papaverlapponicumsubsp.labradoricum (Fedde) Knaben, Blyttia 16: 78. 1958.

##### ﻿3.6.6. Oreomecon lapponica subsp. lapponica

Taxon classificationPlantaeRanunculalesPapaveraceae

(Tolm.) Galasso, Banfi & Bertolucci, Pl. Rev. 5(4): 58. 2023

4FC3E5B5-EC86-51E3-91A5-31D51FA9B681

≡ Papaverradicatumsubsp.lapponicum Tolm., Bot. Mater. Gerb. Glavn. Bot. Sada R.S.F.S.R., 4: 86. 1923. Type: Russia, Kola Peninsula, Oz. Imandra, 1 Aug 1911, *Pohle s.n.* (lectotype: LE, designated by Egorova [1998] p. 98) ≡ Papaverlapponicum (Tolm.) Nordh., Bergens Mus. Årbog (Årbok), Naturvidensk. Rekke 2: 45. 1931.
= Papavernudicaulesubsp.kvaenangense C.E.Lundstr., Acta Horti Berg. 7: 416. 1923; Type: Norway, Troms, Kvænangen, Burfjorddalen, Jul-Aug 1901, *A.Notø* (lectotype: TROM, lectotype designated by Löve [1962a] p. 133 ≡ Papaverlapponicumsubsp.kvaenangense (C.E.Lundstr.) Ö.Nilsson, Nordic J. Bot. 20: 522. 2001;
= Papaverlapponicumsubsp.scandinavicum Knaben, Opera Bot. 2; 3: 56. 1959. Type: Norway: Finnmark, Alta, Talvik, Vassbotndalen, *S.E. Olsen* (holotype: O).
= Papaverchibinense Semenova, Fl. Murmansk. Obl. 3: 368. 1956. Type: [Russia, Kola Peninsula] Khibinskii gornyi massiv, dolina ozera M. Vud’javr, kamenistyi otkos za morenoi meshchdy gorami Poachvumchorr i Takhtavumchorr, 8 Aug 1954, *N. Semenova-Tjan-Schanskaja 127* (holotype: LE).
= Papavernorvegicum Semenova, Fl. Murmansk. Obl. 3: 369. 1956. Type: Norway: Finnmark, Alta, Talvik, Vassbotndalen, 1930, *R. Nordhagen* (holotype: S).
= Papavertolmatchevii Semenova, Fl. Murmansk. Obl. 3: 369. 1956. Type: [Russia, Kola Peninsula] In montibus Chibinensibus ad declivitatem austro-orientalem montis Rasvumchorr, 4 Jul 1955, *N. Semenova-Tjan-Schanskaja 88* (holotype: LE).

##### ﻿3.6.7. Oreomecon lapponica subsp. occidentalis

Taxon classificationPlantaeRanunculalesPapaveraceae

(C.E.Lundstr.) Elvebakk & Bjerke comb. nov.

1620D5FA-9E72-5F5C-B62C-74F0810A1D99

urn:lsid:ipni.org:names:77350936-1

≡ Papaverradicatumsubsp.occidentale C.E.Lundstr., Acta Horti Berg. 7, 5: 413. 1923. Type: [Greenland]: Groenlandia orientalis, Sabine Island, 10 Jul 1899, *P. Dusén 325* (lectotype: S [barcode S07-10363], lectotype designated by Elven et al. [2009] p. 987) ≡ Papaverlapponicumsubsp.occidentale (C.E.Lundstr.) Knaben; Opera Bot. 2, 3: 413 (1959).
= Papaverlapponicasubsp.porsildii Knaben, Blyttia 16: 79. 1958. Type: Canada: Nunavut, “Middle Territories, Foxe Basin, Prince Charles Island”, *A.E.Porsild* (holotype: CAN).

### ﻿3.7. Non-Arctic Northern Europe

#### ﻿Notes

In his monograph on the alpine *Papaver* taxa in Scandinavia, Nordhagen (1932) described three new species. In addition, he described four new subspecies and two new varieties, including one variety of *P.radicatum* Rottb. He also briefly mentioned a ‘doubtful race’ (“± zweifelhaften Rasse”), which was intermediate between P.radicatumsubsp.dovrense Nordh. and *P.relictum* (E.Lundstr.) Nordh., but it was not described. His suggested name, P.radicatumsubsp.dovrensevar.intermedium Nordh., for this taxon is, therefore, a *nomen nudum*, which makes later homotypic recombinations of this name illegitimate. A replacement name is, therefore, introduced below and a diagnosis is supplied; for a further description, see Nilsson (2001).

In a flora treatment, Löve (1945) described P.radicatumsubsp.stefanssonii Á.Löve from Iceland, including both the white- or pink-flowered plants P.radicatumf.albiflora Stefánsson and P.radicatumf.rubriflora Stefánsson presented in the flora by Stefánsson (1901). However, like these, the taxon is illegitimate as no Latin diagnosis was provided (Art. 39.1 in the Code; Turman et al. (2018)) and the name cannot be applied to homotypic recombinations (Art. 6.4 in the Code; Turman et al. (2018)). Löve (1955, 1962b) interpreted the type material of *P.radicatum* to originate from Greenland and considered this species to be limited to Greenland and Canada. Löve (1955) considered Nordic material to belong to four species, namely *P.steindorssonianum* Á.Löve and *P.stefanssonianum* Á.Löve from Iceland and *P.relictum* and *P.nordhagenianum* Á.Löve from Scandinavia. In addition, he accepted *P.lapponicum* and *P.laestadianum* (Nordh.) Nordh. from northernmost Fennoscandia. By an epithet name change, *Papaverstefanssonianum* became the valid basionym of the taxon now often treated as P.radicatumsubsp.stefanssonii, for example, by Nilsson (2001) and Wąsowicz (2020).

Löve (1962a) revised his concepts and transferred the octoploid species *P.lapponicum* and *P.laestadianum* to the subspecies level of his concept of the North American octoploid *P.radicatum*. In contrast, all Nordic decaploid taxa were united within one species. When merging *P.nordhagenianum* and *P.relictum*, he described five subspecies of *P.nordhagenianum*, three of which also have several varieties. His selected species is the younger of the two alternatives and, as already shown by Knaben and Hylander (1970), the recombinations are not in accordance with the priority rules. Thus, eight of the names published by Löve (1962a) are here considered illegitimate. Löve later reached the same conclusion, as he stated that all these taxa instead belong within *P.relictum* (Löve 1970). However, he only provided valid recombinations of the Icelandic-Faroese taxa.

Knaben (1958) and Knaben and Hylander (1970) argued convincingly why the type of *P.radicatum* originated in Iceland. The view that *P.radicatum* is a North Atlantic taxon known primarily from alpine localities in Norway, Sweden, Iceland (with secondary localities in lowland screes and river banks) and the Faroe Islands has been maintained later (Elven et al. 2011; 2022) and subspecific taxa described from elsewhere all represent other species (Solstad et al. 2003). Knaben (1970) stated that her intention was to treat the *P.radicatum* taxa at the variety level. However, she later concluded that this might have led to confusion in light of the high number of synonyms already published (Knaben 1985).

Most of the Scandinavian taxa were studied morphometrically by Selin and Prentice (1988) and Selin (1998, 2000), who referred to these taxa as subspecies. Nilsson (2001) treated 13 Nordic taxa at the subspecies level and provided morphological descriptions and a determination key. Solstad et al. (2003), however, did not find support for such a diversification, based on an analysis of isozyme patterns and a later AFLP-based study concluded on the presence of two groups, one comprising populations from northern Scandinavia, another one comprising populations from southern Scandinavia and Iceland (Solstad et al. 2009). Elven et al. (2011, 2022) maintained that the variation in the North Atlantic area does not, with one exception, merit recognition as a subspecies. In a checklist from Iceland, Wąsowicz (2020), on the other hand, recently accepted three subspecies and the distribution of two of these subspecies have previously been mapped, showing that they reach the Arctic parts of Iceland (Nilsson 2001).

*Papaverradicatum* has been a key issue in Nordic discussions on whether plant life survived the Weichselian glaciation or immigrated post-glacially, for example, the review by Solstad et al. (1999, 2003) and the discussion by Selin (2000). The species has been extensively studied by all generations of Nordic botanists, still without a unified conclusion on its taxonomy. However, Elven et al. (2022) remarked that future application of other genetic markers might instigate a revised taxonomic concept for this species. To reflect the Nordic name tradition and facilitate communication, Elven et al. (2022), therefore, treated nine formerly named taxa of *P.radicatum* from mainland Norway at the variety level; this was done as an informal treatment without providing the required recombinations. The same approach has been followed by Artsdatabanken (2024).

Below, we present a review of the classifications of the taxa within *P.radicatum*, with all important synonyms cited as presented in the original literature, involving a number of deviations from those listed by Nilsson (2001) and POWO (2023). In the absence of modern molecular data, we use morphological criteria and vicariant evolution as criteria for accepting subspecies, as underlined by Molinari (2023) and POWO (2023). Assessments of morphological differentiation rely on the morphometric studies by Selin and Prentice (1988), Selin (1998; 2000) and Øvstedal and Grung (2015).

In a study on five of the entities from southern Norway, Selin and Prentice (1988) concluded that P.radicatumsubsp.intermedium (Nordh.) Knaben and P.radicatumsubsp.oeksendalense Knaben were distinct, whereas P.radicatumsubsp.groevudalensis Knaben and P.radicatumsubsp.gjaerevollii Knaben were clustered quite closely with P.radicatumsubsp.ovatilobum Tolm. from a neighbouring mountain area. When recombining these taxa in *Oreomecon* below, we therefore treat P.radicatumsubsp.ovatilobum, P.radicatumsubsp.oeksendalense and the new name of P.radicatumsubsp.intermedium at the subspecies level. The former is the older of the three names from mountains further to the north in southern Norway and takes priority when P.radicatumsubsp.groevudalensis and P.radicatumsubsp.gjaerevollii are treated as synonyms, as done also by Nilsson (2001). Øvstedal and Grung (2015) concluded that the sixth southern Norwegian entity, P.radicatumsubsp.relictum was morphometrically distinct from P.radicatumsubsp.oeksendalense and deserved its position at the subspecies level, a conclusion followed here.

Concerning northern Scandinavia, Selin (1998) found P.radicatumsubsp.subglobosum Nordh. to be morphologically distinct, whereas P.radicatumsubsp.hyperboreum Nordh. and P.radicatumsubsp.macrostigma (Nordh.) Nordh. were similar. The latter was originally described as P.radicatumsubsp.hyperboreumvar.macrostigma Nordh. by Nordhagen (1932). The local endemic *P.radicatum* subsp. *avkoënse* Knaben has not been studied morphometrically by the studies referred to above. Only P.radicatumsubsp.subglobosum and P.radicatumsubsp.hyperboreum are, therefore, recombined at the subspecies level below. Material from Iceland and the Faroe Islands has not been subject to morphometric analyses, except for unpublished data referred to by Selin (2000). Here, the taxon known as P.radicatumsubsp.stefanssonii was related to south Scandinavian taxa in seed characters and to northern Scandinavian material in capsule morphology. It also deviates from all entities in its range of flower colours and is accepted here as a subspecies together with the nominate subspecies.

The conclusion below is that *Oreomeconradicata* is accepted with eight subspecies and four varieties from the Nordic area.

#### ﻿The identity of Papaverradicatumsubsp.laestadianum Nordh.

The exception referred to above is P.radicatumsubsp.laestadianum Nordh., a name used for a taxon limited to a small alpine area of Troms in north Norway and adjacent Sweden (Solstad et al. 2009). It shares the chromosome number (2*n* = 56) with *P.lapponicum*, contrasting 2*n* = 70 for *P.radicatum*. However, P.radicatumsubsp.laestadianum was not integrated into the biosystematic study by Knaben (1959a; b) as material was not available for her extensive cultivation experiments. Nannfeldt (1963) included it in *P.lapponicum* and Knaben (1983) concluded that it deserves status as a subspecies of *P.lapponicum*.

Tromsø Arctic-Alpine Botanic Garden holds material of P.radicatumsubsp.laestadianum in cultivation from the locality Isdalsfjella/Njearrečazagáisi. This corresponds to the place name “Causigaisa” used in older maps and is within the area where the type material of this taxon was collected (Nordhagen 1932). Nordhagen reported it from two additional localities and provided illustrations of samples from two of them. However, he did not designate any type or mention any type of candidate and a lectotypification is therefore required. Amongst the syntypes at O and shown by Natural History Museum, University of Oslo (2023), there are annotations regarding two alternative typifications. One includes two sheets, O-V-2017486 and O-V-2017487, with at least five different individuals, as shown by the presence of tap roots, in addition to nine rosettes and 20 single leaves and references are given to illustrations from both sheets published by Nordhagen (1932, 1970). The annotations are undated and the handwriting is by T. Engelskjøn, although not formally documented. An alternative typification is presented by the annotation “Typus: individual marked ‘NB’ (by R. Nordhagen?) 30 Jun 1978, Gunvor Knaben”. This refers to the specimen to the far left on the latter of these sheets, which is a well-defined individual, as shown by its tap root. None of these annotations fulfils the requirement of effective publication (Art. 7.10 in the Code; Turland et al. (2018)) and the taxon is, therefore, lectotypified below. The specimen with Knaben’s annotation is selected as the lectotype. All additional specimens on six sheets in O are designated as isolectotypes.

Fig. 3 shows the capsule and leaf morphology of specimens from the comparative cultivation of four taxa in the Tromsø Arctic-Alpine Botanic Garden. Material from the lectotype locality of Papaverradicatumsubsp.laestadianum shows clear affinity in capsule and leaf morphology with P.lapponicumsubsp.lapponicum and appears very distinct from the two varieties of *P.radicatum* included. The black capsule hairs of our cultivated specimens are smooth in P.radicatumsubsp.laestadianum and *P.lapponicum*, whereas they are decurrently dentate in both varieties of *P.radicatum*. It also differs in several minor characters from P.lapponicumsubsp.lapponicum. We, therefore, interpret P.radicatumsubsp.laestadianum as a subspecies of *P.lapponicum*, prior to its recombination in *Oreomecon* below. Its affinity to *P.lapponicum* is also supported by its chromosome number.

It should be added that a population of P.lapponicumsubsp.laestadianum from the Mountain Márkos, which is situated ca. 15 km north of the type locality, was studied by Solstad et al. (2009) and by Nevermo (1997), both studies concluding on an affinity to *P.radicatum*. However, both Heggelund (1993), who presented its known distribution and Nevermo (1997) commented that plants on Márkos were morphologically heterogeneous. We, therefore, intend to bring samples from more populations of this taxon into comparative cultivation to test its possible heterogeneity pending future molecular studies.

Overall, northern non-Arctic Europe includes one species, distributed within Iceland, the Faroe Islands and the mountains of Scandinavia, in addition to one endemic subspecies of a different species (Fig. 2). The nominate subspecies of *Oreomeconlapponica* is treated as taxon 3.6.6 here.

#### ﻿Rare species and red-list treatments

Only the two fully-accepted subspecies of *P.radicatum* were treated by the Norwegian Red List (Artsdatabanken 2021), both as EN. These subspecies are subsp. radicatumandsubsp.laestadianum. In Sweden, these two taxa are assessed as NT and VU, respectively (Eide et al. 2020). Papaverradicatumsubsp.stefanssonii Knaben was treated as Vulnerable (VU) in Iceland (Wąsowicz and Heiđmarsson 2019) below its homonym P.radicatumsubsp.stefanssonii (Á.Löve) Jonsell & Ö.Nilsson.

#### ﻿Accepted taxa

##### ﻿3.7.1. Oreomecon lapponica subsp. laestadiana

Taxon classificationPlantaeRanunculalesPapaveraceae

(Nordh.) Elvebakk & Bjerke nomencl. nov.

88CD2856-17E1-575D-A139-E4DD52AD00CB

urn:lsid:ipni.org:names:77350937-1

≡ Papaverradicatumsubsp.laestadianum Nordh. Bergens Mus. Årbog (Årbok) 1931 (2): 49. 1932. Type: Norway: Troms, Målselv, Rostadalen: Causigaisa nær grensen mot Moskovarre-Pältsa, på kalkholdig glimmerskifer, ca. 1100 moh., 28 Jul 1930, *R. Nordhagen* (lectotype: O, specimen marked “NB” to the far left on sheet O-V-2017487, lectotypification designated here, isolectotypes designated here: O [barcodes O-V-2017486; O-V-2014582; O-V-2014583; O-V-362047; O-V-362048, specimens other than the lectotype on O-V-2017487]) ≡ Papaverlaestadiananum (Nordh.) Nordh.; Bot. Not. 1939: 693. 1939 ≡ Oreomeconlaestadiana (Nordh.) Krivenko, Nov. Syst. Pl. Vasc. 54: e06:2. 2023.

##### ﻿3.7.2. Oreomecon radicata

Taxon classificationPlantaeRanunculalesPapaveraceae

(Rottb.) Banfi, Bartolucci, J.-M.Tison & Galasso, Nat. Hist. Sci. 9(1): 71. 2022

0EA60CA3-34B4-5F00-9BEA-CCE3AB9176FB

≡ Papaverradicatum Rottb., Skr. Kiøbenhavnske Selsk. Laerd. Elsk. 10: 455. 1770. Type: Ill. in Rottbøll (1770), Tab. VIII, No. XXIV (lectotype designated by Knaben [1958], p. 62. Epitype: Iceland, Barđastrandarsýsla, Brjánslækur, 23 Jul 1962, *Nannfeldt 17564*, UPS [barcode UPS 207575], lower left specimen, designated by Nilsson & Elven in Jonsell [2001], p. 520) ≡ Papavernudicaulevar.radicatum (Rottb.) DC., Syst. Nat. 2: 70. 1821 ≡ Papavernudicaulesubsp.radicatum (Rottb.) Fedde, Beibl. Bot. Jahrb. Syst. 81: 34. 1909.

##### ﻿3.7.3. Oreomecon radicata subsp. radicata

Taxon classificationPlantaeRanunculalesPapaveraceae

(Rottb.) Banfi, Bartolucci, J.-M.Tison & Galasso, Nat. Hist. Sci. 9(1): 71. 2022

C8201116-D171-5BC9-96C6-7E4C787E581F

≡ Papaverradicatum Rottb., Skr. Kiøbenhavnske Selsk. Laerd. Elsk. 10: 455. 1770. Type: Ill. in Rottbøll (1770), Tab. VIII, No. XXIV (lectotype designated by Knaben [1958], p. 62. Epitype: Iceland, Barđastrandarsýsla, Brjánslækur, 23 Jul 1962, *Nannfeldt 17564*, UPS [barcode UPS 207575], lower left specimen, designated by Nilsson & Elven in Jonsell [2001], p. 520.
= Papavernordhagenianumsubsp.islandicum Á.Löve, Nytt Mag. Bot. 4: 16. 1955. Type: Eyri in Ísafjørdur Islandiae occidentalis-septentrionalis, 1925, *I. Óskarsson* (holotype: ICEL) ≡ Papaverrelictumsubsp.faeroensevar.islandicum (Á.Löve) Á.Löve, Taxon 19: 300. 1970 ≡ Papavernordhagenianumsubsp.faeroensevar.islandicum (Á.Löve) Á.Löve, Taxon 11: 137. 1962, nom. illeg.

##### ﻿3.7.4. Oreomecon radicata var. avkoensis

Taxon classificationPlantaeRanunculalesPapaveraceae

(Knaben) Elvebakk & Bjerke, comb. et stat. nov.

B2AB6E34-1F06-5FF5-8877-570017DB85D0

urn:lsid:ipni.org:names:77350938-1

≡ Papaverradicatum subsp. *avkoënse* Knaben, Opera Bot. 2 (3): 39. 1959. Type: Plant grown from seed collected in Norway, Troms, Nordreisa, Avko in 1952 by *O. Gjærevoll* (holotype: O).
= Papavernordhagenianumsubsp.nordhagenianum var. *avkoënse* (Knaben) Á.Löve, Taxon 11: 136, nom. illeg.

##### ﻿3.7.5. Oreomecon radicata var. faeroensis

Taxon classificationPlantaeRanunculalesPapaveraceae

(C.E.Lundstr.) Elvebakk & Bjerke, comb. et stat. nov.

D0F1F843-A041-5B19-9E62-E7D64CDE3B59

urn:lsid:ipni.org:names:77350939-1

≡ Papaverradicatum subsp. *faeroënse* C.E.Lundstr., Acta Horti Berg. 7: 412. 1923. Type: Faroe Islands, Fugloy, *Harz & Ostenfeld* (lectotype: C, designated by Löve [1962a], p. 137) ≡ Papavernordhagenianum subsp. *faeroënse* (C.E.Lundstr.) Á.Löve, Nytt Mag. Bot. 4: 16. 1955 ≡ Papaverrelictumsubsp.faeroense (C.E.Lundstr.) Á.Löve, Taxon 19: 300. 1970.

##### ﻿3.7.6. Oreomecon radicata subsp. hyperborea

Taxon classificationPlantaeRanunculalesPapaveraceae

(Nordh.) Elvebakk & Bjerke comb. nov.

740797BB-7B06-5675-A559-9D2960FCCB83

urn:lsid:ipni.org:names:77350940-1

≡ Papaverradicatumsubsp.hyperboreum Nordh., Bergens Mus. Årbok 1931, Naturv. r. 2; 48. 1932. Type: Norway, Troms, Målselv, Alappen, 30 Jul 1930, *R. Nordhagen* (holotype: O).
= Papavernordhagenianumsubsp.nordhagenianum Á.Löve, Nytt Mag. Bot. 4: 15. 1955. Type: Nissontjåkko, Lapponia tornensis Sueciae, *H. Smith* (holotype: UPS).

##### ﻿3.7.7. Oreomecon radicata subsp. knabeniana

Taxon classificationPlantaeRanunculalesPapaveraceae

Elvebakk & Bjerke, comb., stat. et nom. nov.

12C36CAA-4B45-5C1E-B4AA-B283FE66D498

urn:lsid:ipni.org:names:77350941-1

≡ Papaverradicatumsubsp.dovrensevar.intermedium Nordh., Bergens Mus. Årbok 1931, Naturv. r. 2; 43. 1932, nom. nud. Type: Norway, Oppland, Vågå, Besshøe, 1923, *R. Nordhagen* (holotype: BG) ≡ Papaverradicatumsubsp.intermedium (Nordh.) Knaben, Opera Bot. 2(3): 34. 1959, nom. illeg. ≡ Papavernordhagenianumsubsp.ovatilobumvar.intermedium (Nordh.) Á.Löve, Taxon 11: 136. 1962, nom. illeg.

###### Diagnosis.

Differs from O.radicatasubsp.ovatiloba in ovoid capsules which are wider near the top and not near the middle, capsules which are densely covered by pale brown, mostly appressed and not suberect hairs and leaf lobes often lanceolate to ovate and not ellipsoid to ovate.

###### Etymology.

Oreomeconradicatasubsp.knabeniana is named in honour of the substantial contributions to the knowledge of this genus made by Gunvor Snekvik Knaben (1911–1993), who was affiliated with the University of Oslo during most of her career.

##### ﻿3.7.8. Oreomecon radicata var. macrostigma

Taxon classificationPlantaeRanunculalesPapaveraceae

(Nordh.) Elvebakk & Bjerke comb. nov.

61A5E32D-E35F-5E68-8859-0979DFB79B9F

urn:lsid:ipni.org:names:77350942-1

≡ Papaverradicatumsubsp.hyperboreavar.macrostigma Nordh., Bergens Mus. Årbok 1931, Naturv. r. 2; 48. 1932. Type: Norway, Finnmark, Stjernøya, Hundneset, 14 Jul 1930, *R. Nordhagen* (lectotype: O, designated by Elven & Nilsson in Jonsell [2001], p. 521) ≡ Papaverradicatumsubsp.macrostigma (Nordh.) Nordh., Norsk flora: 225. 1940 ≡ Papavernordhagenianumvar.macrostigma (Nordh.) Á.Löve, Nytt Mag. Bot. 4: 15. 1955.

##### ﻿3.7.9. Oreomecon radicata subsp. oeksendalensis

Taxon classificationPlantaeRanunculalesPapaveraceae

(Nordh.) Elvebakk & Bjerke comb. nov.

8166E677-E91D-5190-A0D1-9D4B7721B9E7

urn:lsid:ipni.org:names:77350943-1

≡ Papaverradicatumsubsp.oeksendalense Knaben, Opera Bot. 2(3): 38. 1959. Type: Plant grown from seed collected in Norway, Møre & Romsdal, Sunndal, Øksendalen, Jønnstadnibba, 4 Aug 1948, *R. Nordhagen* (holotype: O); ≡ Papavernordhagenianumsubsp.ovatilobumvar.oeksendalense (Knaben) Á.Löve, Taxon 11: 136. 1962, nom. illeg.
= Papaverangusticarpum Nordh., Norsk Flora: 629. 1970, nom. nud.

##### ﻿3.7.10. Oreomecon radicata subsp. ovatiloba

Taxon classificationPlantaeRanunculalesPapaveraceae

(Tolm.) Elvebakk & Bjerke comb. nov.

C52DF402-7C21-575E-9108-AAACBC7E8D76

urn:lsid:ipni.org:names:77350944-1

≡ Papaverradicatumsubsp.ovatilobum Tolm., Bot. Mater. Gerb. Glavn. Bot. Sada RSFSR 4: 85. 1923. Type: Norway, Sør-Trøndelag, Oppdal, Kongsvold, Aug 1889, *G.H. Hagelin* (lectotype: S, designated by Löve [1962a] p. 136) ≡ Papavernordhagenianumsubsp.ovatilobum (Tolm.) Á.Löve, Nytt Mag. Bot. 4: 15. 1955.
= Papaverradicatumsubsp.gjaerevollii Knaben, Opera Bot. 2 (3): 38. 1959. Type: Plant grown from seed collected in Norway, Sør-Trøndelag, Trollheimen, Gjevilvasskammen by *O. Gjærevoll* (holotype O); = Papavernordhagenianumsubsp.ovatilobumvar.gjaerevollii (Knaben) Á.Löve, Taxon 11: 136. 1962, nom. illeg.
= Papaverradicatumsubsp.groevudalense Knaben, Opera Bot. 2 (3): 38. 1959. Type: Plant grown from seed collected in Norway, Møre, Sunndalen, Grøvudalen by *R. Nordhagen* (holotype: O); = Papavernordhagenianumsubsp.ovatilobumvar.groevudalense (Knaben) Á.Löve, Taxon 11: 136. 1962, nom. illeg.

##### ﻿3.7.11. Oreomecon radicata subsp. relicta

Taxon classificationPlantaeRanunculalesPapaveraceae

(C.E.Lundstr.) Elvebakk & Bjerke comb. nov.

A239C610-A08C-5B7C-BADD-FE3A019C15BB

urn:lsid:ipni.org:names:77350945-1

≡ Papavernudicaulesubsp.relictum C.E.Lundstr., Acti Horti Berg. 7: 415. 1923. Type: Norway, Oppland, Vang, Vassendfjeld, 15 Aug 1870, *Söderén & Eisen* (lectotype: S, designated by Elven & Nilsson in Jonsell [2001], p. 521) ≡ Papaverradicatumsubsp.relictum (C.E.Lundstr.) Tolm., Svensk Bot. Tidskr. 21: 78. 1927 ≡ Papaverrelictum (C.E.Lundstr.) Nordh., Bergens Mus. Årbok 1931, Naturv. r. 2; 45. 1932; = Papavernordhagenianumsubsp.relictum (C.E.Lundstr.) Á.Löve, Taxon 11: 136. 1962, nom. illeg.

##### ﻿3.7.12. Oreomecon radicata subsp. stefanssoniana

Taxon classificationPlantaeRanunculalesPapaveraceae

(Á.Löve) Elvebakk & Bjerke, comb. et stat. nov.

EA811291-556B-5DDE-AB32-4739BEA8D9AE

urn:lsid:ipni.org:names:77350946-1

≡ Papaverstefanssonianum Á.Löve, Nytt Mag. Bot. 4: 14. 1955. Type: Iceland, Norđvestur-Ísland, Gufudalsháls, 8 Aug 1893, *S.Stefánsson & Ó.Davíđsson* (lectotype: ICEL, designated by Löve [1955] p. 14).
= Papaverradicatumf.albiflora Stefánsson, Fl. Islands: 100. 1901, nom. illeg; = Papaverradicatumf.rubriflora Stefánsson, Fl. Islands: 100. 1901, nom. illeg; = Papaverradicatumsubsp.stefanssonii Á.Löve, Izlendsk. Jurt.: 149. 1945, nom. illeg.; – Papaverradicatumsubsp.stefanssonii (Á.Löve) Jonsell & Ö.Nilsson, in Jonsell (2001): 521, nom. illeg.; – Papavernordhagenianumsubsp.stefanssonii Á.Löve, Taxon 11: 137. 1962, nom. illeg.; – Papaverrelictum subsp. *faeroënsis*var.stefanssonii (Á.Löve) Á.Löve, Taxon 19: 300. 1970, nom. illeg.

##### ﻿3.7.13. Oreomecon radicata var. steindorssoniana

Taxon classificationPlantaeRanunculalesPapaveraceae

(Á.Löve) Elvebakk & Bjerke comb. nov.

A997035C-4104-502B-BC35-721536C7956A

urn:lsid:ipni.org:names:77350947-1

≡ Papaversteindorssonianum Á.Löve, Nytt Mag. Bot. 4: 15. 1955. Type: Iceland, Austur-Ísland, Ós i Breiđdalur, Aug 1944, *S.Steindórsson* (holotype: AMNH) ≡ Papaverrelictum subsp. *faeroënsis*var.steindorssonianum (Á.Löve) Á.Löve, Taxon 19: 300. 1970 ≡ Papaverradicatumsubsp.steindorssonianum (Á.Löve) Knaben ex Ö. Nilsson, Nordic J. Bot. 20: 521. 2001.
= Papavernordhagenianum subsp. *faeroënse*var.steindorssonianum (Á.Löve) Á.Löve, Taxon 11: 137. 1962, nom illeg.

##### ﻿3.7.14. Oreomecon radicata subsp. subglobosa

Taxon classificationPlantaeRanunculalesPapaveraceae

(Nordh.) Elvebakk & Bjerke comb. nov.

E0F5A4DE-2B9C-5401-8A89-12F00DEB0B61

urn:lsid:ipni.org:names:77350948-1

≡ Papaverradicatumsubsp.subglobosum Nordh., Bergens Mus. Årbok 1931, Naturv. r. 2; 47. 1932. Type: Norway, Nordland, Meløy, Svartisen, Engabreen, 1 Aug 1930, *R. Nordhagen* (holotype: O) ≡ Papavernordhagenianumvar.subglobosum (Nordh.) Á.Löve, Nytt Mag. Bot. 4: 16. 1955.

###### Notes.

The origin of the seeds for the cultivated samples shown in Fig. 3 are:

Oreomeconlapponicasubsp.lapponica - *ex* Norway, Troms, Kvænangen, Raudfjellet, Aug 1988, *A. Elvebakk*, TAABG 1992-4053.

Oreomeconlapponicasubsp.laestadiana - Norway, Troms, Målselv, N slope of Isdalsfjellet/Njearrečazagáisi facing Čorrováhgáisi, schistose scree, 1000 m alt., 23 Aug 2011, *A. Granmo*; *L. Mølster*, *I.A. Mølster*, TAABG 2014-248/TROM V-991064.

Oreomeconradicatasubsp.ovatiloba - Botanic Garden of Tøyen, Oslo; TAABG 1992-681.

Oreomeconradicatasubsp.stefanssoniana - undocumented commercial source; TAABG 2001-76.

### ﻿3.8. Central Europe

#### ﻿Notes

Kadereit (1990) monographed the *Papaveralpinum* L. complex, including eight subspecies with mostly non-overlapping distribution ranges. All these subspecies were later subject to an RAPD analysis, which produced five weakly-supported geographically-based clusters (Bittkau and Kadereit 2002). The correlation with subspecies was low. They also analysed four subspecies with respect to ITS1 sequences, which did not show any differentiation.

Schönswetter et al. (2009) studied 12 named entities by using DNA sequencing, AFLP fingerprinting and morphological traits. They concluded with a similar set of four weakly-supported geographically-based groups (Slovenia, Balkan, most of the Alps/Tatra, Central Italy) and two more strongly-supported groups from south-eastern France and the Pyrenees. They did not find any consistent morphological or molecular characters differentiating the taxa. They concluded that all the previously named variation was best treated within a single, widely defined species, *Papaveralpinum*, except for the Iberian entity, which was accepted as P.alpinumsubsp.lapeyrousianum (Gutermann ex Greuter & Burdet) Kerguélen. They found most sampled population groups or populations to be genetically distinct and explained this as genetic drift within diploid and rapidly reproducing plants in often small populations. The concept of *P.alpinum* used by Schönswetter et al. (2009) was followed by Banfi et al. (2022) when the latter recombined P.alpinumand itssubsp.lapeyrouseanum into *Oreomecon*, although Banfi et al. (2022) applied a different subspecies epithet for the Iberian entity.

The classification system by Schönswetter et al. (2009) has been followed by many treatments, such as Hassler (2023a, b), although the two latter studies made an exception for *Papavertatricum* (A.Nyár.) Ehrend. ex Soó and P.tatricumsubsp.fatraemagnae Bernát, which were accepted as separate entities. POWO (2023) accepted a widely defined *Oreomeconalpina*. However, they also made exceptions by maintaining four taxa in the complex as separate *Papaver* species. *Flora Gallica* (Tison and de Foucault 2014) also adopted *P.alpinum* in a broad sense. In contrast, other major floras from the area, for example, *Flora Helvetica* (Lauber et al. 2018) and *Flora d’Italia* (Pignatti et al. 2017), adopted a multi-species approach.

Fragnière et al. (2020) and Pittet et al. (2020) studied in detail *Papaveroccidentale* (Markgr.) H.E.Hess et al., a white-flowered species from the western Alps. It appears similar to the other white-flowered species, *P.alpinum* s.str., *P.sendtneri* Kern. ex Hayek and *P.tatricum* (A.Nyár.) Ehrend. and might be characterised as the least distinct taxon, at least from its original description as Papaveralpinumsubsp.tatricumvar.occidentale Markgr. (Markgraf 1958a). Based on a detailed genetic study, Pittet et al. (2020) concluded that *P.occidentale* is a genetically and morphologically well-defined entity. It has apparently survived the Late Glacial Maximum both in periglacial areas and in nunatak situations. They underlined that further studies are needed to sort out the taxonomy of this complex.

The emerging pattern is that of an immigrating ancestral taxon into Central Europe, which had split into a western and a central group, with further differentiations leading to genetically distinct populations in numerous discrete areas (Schönswetter et al. 2009). As indicated by Schönswetter et al. (2009), the immigration and expansion situations are more likely to have taken place during a glacial period. In contrast, an interstadial represents a bottleneck situation favouring isolation and differentiation within this complex.

The studies by Bittkau and Kadereit (2002) and Schönswetter et al. (2009) indicate that some names do not correlate with the patterns in the molecular studies. This mismatch in the data by Bittkau and Kadereit (2002) would have been reduced if accepting *P.aurantiacum* Loisel. and P.alpinumsubsp.occidentale. The southern populations of P.alpinumsubsp.kerneri (Hayek) Fedde in Bosnia and Herzegovina and Montenegro may also represent separate entities, according to data from Schönswetter et al. (2009). With their present names, plants in central Italy do not match the molecular data shown in these two studies. Conversely, other names appear to be redundant, for example, P.alpinumsubsp.victoris (Škornik & Wraber) Wraber from Slovenia, although the conclusion by Škornik and Wraber (1988) was based on a comparison with the neighbouring species. When treating this difficult complex within *Oreomecon*, we find that plants of the central group are better maintained at the subspecies level than left unnamed. The study by Schönswetter et al. (2009) is here used as the main source on nomenclature.

Markgraf (1958a) is the only study on the complex where type localities are indicated, although with incomplete information. The Bulgarian Papaveralpinumsubsp.degenii (Urum. & Jáv.) Markgr. was cited with type specimen from “El Tepe, Pirin, 1915, *Dimonie*“, whereas the locality which had been presented in the protologue by Urumov (1920) was “in graminosis aridis m. Pirin, legi 1915” without collector information. In a biography on Mihael Dimonie, Pachschwöll et al. (2019) presented his botanical activities, indicating that he instead only visited the Pirin Mountains in June, July and August 1909. The area was then within the Ottoman Empire and the peak of Mt. Vihren was then referred to as El Tepe or Jel-tepe. His only collections surviving two fires are those distributed commercially to several herbaria under the heading “Plantae Macedonicae”. Three of his collections of this taxon from Vihren at WU, as documented by Virtual Herbaria JACQ (2024), have slightly different label texts from the one in the protologue by Urumov (1920). However, in the absence of any known collections from 1915, these are designated as lectotypes and isolectotypes below. The label texts are identical, except that the lectotype includes altitude information.

According to Schönswetter et al. (2009), *Papaveraurantiacum* Loisel. was described in Flora Gallica (Loiseleur-Deslongchamps 1807). However, it was described in a later supplement (Loiseleur-Deslongchamps 1809), where it was compared with the leaves and flowers of *Papaveralpinum* and where a type specimen was cited for having been collected at Mont Ventoux by M. Requien. We suppose the holotype specimen is at P, but we have not been able to trace it there or elsewhere.

Zapałowicz (1911) described *Papavercorona-sancta-stephani* Zapał. as common at 2000–2200 m alt. on the northwest slope of Mt. Ineu (= Vârful Ineu, Munţii Rodnei) in the Romanian Carpathians and Paszko et al. (2020) indicated that the type is housed at KRAM. Zapałowicz (1911) described the type locality, but did not select a type. We have seen six vouchers of the typical form from KRAM. The article by Zapałowicz (1911) was published in October 1911 and a collection made in August 2011 by D. Herbich is referred to. Therefore, four vouchers from the period August 2010 to August 2011 are candidates for lectotypification. Below, we designate the specimen out of the five that presents the best-developed floral buds as the lectotype. Zapałowicz (1911) also described Papavercorona-sancta-stephanif.hispidulum Zapał. and P.corona-sancta-stephanivar.angustisectum Zapał. from the same locality. The former is present as a single collection at KRAM and is interpreted by us as the holotype. The latter is lectotypified below, while both are listed as heterotypic synonyms of *P.corona-sancta-stephani* s.str.

Markgraf (1958a) is correct in stating that the name *Papaverrhaeticum* Leresche was first mentioned by Gremli (1874) in the second edition of his “Excursionsflora für die Schweiz”. however, on p. 80 and not on p. 66, as repeatedly stated in basionym citations. Page 66 instead refers to the similar treatment in the sixth flora edition (Gremli 1889), as indicated by Fedde (1909). In both these floras, the name is presented as a *nomen nudum* and the treatment by Gremli (1889) is only ‘like *P.pyrenaicum*, but only in Engadin’. Nyman (1889) recombined it as P.alpinumsubsp.rhaeticum (Leresche) Nyman and Banfi et al. (2022) corrected its author citation to P.alpinumsubsp.rhaeticum (Leresche ex Gremli) Nyman. The first valid description of the taxon was made by Markgraf (1958a: 311), who also cited its type specimen from Oberengadin in Switzerland. He did not cite its herbarium affiliation and we have not been able to trace it. Markgraf (1958a) cited his taxon as “Papaveralpinumsubsp.rhaeticum (Ler.) Mkr.”. The correct citation appears to be P.alpinumsubsp.rhaeticum (Leresche ex Gremli) Nyman ex Markgr. for what is the basionym of a described taxon. However, the name presented by Markgraf (1958a) is illegitimate (Art. 6.4 and 58.1 in the Code, Turland et al. (2018)) and a new homotypic replacement name honouring F. Markgraf is, therefore, introduced below.

Nyárády (1942) described Papaveralpinumsubsp.tatricum A.Nyár. from ‘Tatri Magni’ without adding a type. He described P.alpinumsubsp.tatricumvar.angustisectum A.Nyár. and P.alpinumsubsp.tatricumvar.latisectum A.Nyár. with illustrations of leaves from several collections from the Tatra Mountains. He also cited two collections of P.alpinumsubsp.tatricum from Haute-Savoie in France; however, did not describe the nominate variety of this subspecies. Markgraf (1958a) described the plants from France as P.alpinumsubsp.tatricumvar.occidentale Markgr. with one of the samples mentioned by Nyárády (1942) as the holotype. He also provided a diagnosis of P.alpinumsubsp.tatricumvar.tatricum A.Nyár. and listed as type a collection from Hohe Tatra with Nyárády as collector. This is defined here as the lectotype of P.alpinumsubsp.tatricum, but we have not been able to localise it. The type of *Papaversendtneri* Kern. ex Hayek at B is possibly a holotype, but we have not been able to verify whether duplicates exist, requiring a lectotypification.

Krivenko (2023) recently recombined one of the entities of the *O.alpina* complex as *Oreomecontatrica* (A.Nyár.) Krivenko, whereas Grey-Wilson (2023)introduced the name *Oreomeconcorona-sancti-stephani* (Zapał.) Grey-Wilson. Both these authors selected single taxa from amongst those included within a broad concept of *O.alpina* as defined by Banfi et al. (2022), leaving the remaining ones embedded in *O.alpina* without explaining why these did not deserve similar treatment. As they did not cite any studies supporting these selected recombinations, the two names are not accepted below.

On the other hand, the western entities were well separated from the remaining samples in the analysis by Schönswetter et al. (2009) and the Iberian plants are treated as a separate species here. As shown by Banfi et al. (2022), the name P.pyrenaicumsubsp.suaveolens P.Fourn. is heterotypic as compared with *P.suaveolens* Lapeyr. The latter is illegitimate since the earlier name *P.aurantiacum* Loisel. was cited as a synonym, which prevents the epithet “*suaveolens*” from being adopted at the species level for the basionym P.pyrenaicumsubsp.suaveolens P.Fourn. Greuter (1981)introduced the name *Papaverlapeyrousianum* Gutermann ex Greuter & Burdet. Banfi et al. (2022) concluded that this name has priority at the species level, while Ferrer-Gallego (2024) recently recombined it into *Oreomecon*.

Kropf et al. (2006) studied the Iberian populations and concluded on vicariance in the development of populations both in the Sierra Nevada, the eastern Pyrenees and the western Alps, while long-distance dispersal, surprisingly from Sierra Nevada to Central Pyrenees, explains the latter populations. Relying on genetic structure and morphological dissimilarity, they concluded that the eastern Pyrenean populations are different and deserve a separate variety name, which had been introduced by Ascherson (1869). Both Bittkau and Kadereit (2002) and Schönswetter et al. (2009) confirm that the Iberian populations are heterogeneous. Thus, we follow Kropf et al. (2006) and conclude that the Sierra Nevada and Central Pyrenean populations represent one distinct subspecies within *Papaverlapeyrouseanum*, namely subsp.lapeyrouseanum, which is different from the east Pyrenean P.lapeyrouseanumsubsp.endressii (Asch.) Greuter & Burdet.

The AFLP analysis by Schönswetter et al. (2009) showed that both the Iberian material and samples corresponding to *P.aurantiacum* are genetically very distinct from the remaining parts of the complex. We also recombine the latter taxon at the species level below to reflect this pattern.

#### ﻿Distribution

The present list of accepted taxa includes three species with 11 additional subspecies distributed within the area presented in Fig. 2.

#### ﻿Rare populations and red-listed taxa

Fragnière et al. (2020) conclude that *P.occidentale* is doomed to extinction in the wild due to rapid global warming. Their hypothesis can probably be extended to other taxa of the group. *Papaveraurantiacum*, *P.occidentale* and *P.sendtneri* are Red-listed as near threatened (NT) in Switzerland (Bornand et al. 2016) and *P.alpinum* s.lat. is threatened in Germany (Metzing et al. 2018). From Sierra Nevada, Blanca et al. (2002) presented the orange-flowered *P.lapeyrousianum* as forming a single population of less than 2,500 individuals known from four very small subareas between 3,200 and 3,450 m a.sl., near the peak of Mulhacén. The Sierra Nevada population is obviously in danger, as opposed to the larger populations in the Pyrenees, which are considered to be of least concern (LC).

*Papaverdegenii* is a local endemic of the Mountain Pirin in Bulgaria, where it is rare and occurs between 1,915 and 2,850 m alt.. It has been treated at the species level and as vulnerable (VU) both by Stoeva (2009) and by later online versions of the Bulgarian Red Data Book (Stoeva 2023), where a distribution map shows three population centres, with single minor occurrences, each consisting of 20–60 individuals over areas of only a few m^2^. The species is protected and listed as a glacial relict. Gorgorov et al. (2011) showed that the taxon had reduced sexual reproduction capacity and they tried *in vitro* propagation as an additional effort in *ex-situ* conservation. In Romania, the distribution and ecology of P.alpinumsubsp.corona-sancti-stephani (Zapał.) Borza was presented by Bartók et al. (2016) and its Red-list status in Romania was cited as “rare”, referring to Oltean et al. (1994). The rarest taxon in Central Europe is probably P.tatricumsubsp.fatraemagnae Bernát., a taxon with deviating flowers with wedge-shaped petals known from limestone slopes at only 890 m altitude, at a locality in Slovakia, where it is isolated from the distribution area of *P.tatricum* s.str. (Bernátóvá 2002).

#### ﻿Accepted taxa

##### ﻿3.8.1. Oreomecon alpina

Taxon classificationPlantaeRanunculalesPapaveraceae

(L.) Banfi, Bartolucci, J.-M.Tison & Galasso, Nat. Hist. Sci. 9(1): 69. 2021

CC38A569-D2A8-5226-9A0C-0236B9F6C08B

≡ Papaveralpinum L., Sp. Pl. 507. 1753. Type: [Austria] Niederösterreich, Mount Schneeberg (lectotype: UPS [Herb. Burser IX:58], lectotype designated by Markgraf [1965], p. 145).

##### ﻿3.8.2. Oreomecon alpina subsp. alpina

Taxon classificationPlantaeRanunculalesPapaveraceae

(L.) Banfi, Bartolucci, J.-M.Tison & Galasso, Nat. Hist. Sci. 9(1): 69. 2021

AC143D87-CC1C-5D78-AC54-377205211910

≡ Papaveralpinum L., Sp. Pl. 507. 1753. Type: [Austria] Niederösterreich, Mount Schneeberg (lectotype: UPS [Herb. Burser IX:58], lectotype designated by Markgraf [1965], p. 145).

##### ﻿3.8.3. Oreomecon alpina subsp. corona-sancti-stephani

Taxon classificationPlantaeRanunculalesPapaveraceae

(Zapał.) Elvebakk & Bjerke nomencl. nov.

50913DF3-D5B8-5426-B9F3-E840405581F3

urn:lsid:ipni.org:names:77350949-1

≡ Papavercorona-sancti-stephani Zapał., Bull. Int. Acad. Sci. Cracovie, Cl. Sci. Math., Sér. B, Sci. Nat. 1911(8B): 620. 1911. Type: [Romania: Sub culmine montis] Ineu [ (2280 m) Alpium Rodnensium in valle voraginosa versus septentrionalem occidentem sita, solo mico schistoso 2000–2200 m alt.], 18 Aug 1911, *H. Zapałowicz* (lectotype: KRAM [barcode KRAM00026854]; isolectotype KRAM [barcode KRAM00026853], designated here) ≡ Papaveralpinumsubsp.corona-sancti-stephani (Zapał.) Markgr., Phyton (Horn) 7: 306. 1958 ≡ Papaverpyrenaicumsubsp.corona-sancti-stephani (Zapał.) Borza in Bul. Grǎd. Bot. Univ. Cluj 8: 114. 1928 ≡ Oreomeconcorona-sancti-stephani (Zapał.) Grey-Wilson, Pl. Rev. 5(4): 57. 2023.
= Papavercorona-sancti-stephanif.hispidulum Zapał., Bull. Int. Acad. Sci. Cracovie, Cl. Sci. Math., Sér. B, Sci. Nat. 1911(8B): 621. 1911. Type:: [Romania: Sub culmine montis] Ineu [ (2280 m alt.) Alpium Rodnensium in valle voraginosa versus septentrionalem occidentem sita, solo mico schistoso 2000–2200 m alt.], 18 Aug 1911, *H. Zapałowicz* (holotype: KRAM [barcode KRAM00026857]).
= Papavercorona-sancti-stephanivar.angustiscum Zapał., Bull. Int. Acad. Sci. Cracovie, Cl. Sci. Math., Sér. B, Sci. Nat. 1911(8B): 621. 1911. Type:: [Romania: Sub culmine montis] Ineu [(2280 m alt.) Alpium Rodnensium in valle voraginosa versus septentrionalem occidentem sita, solo mico schistoso 2000–2200 m alt.], 3 Aug 1910, *S. Fedorowicz* (lectotype: KRAM [barcode KRAM00026858], designated here).

##### ﻿3.8.4. Oreomecon alpina subsp. degenii

Taxon classificationPlantaeRanunculalesPapaveraceae

(Urum. & Jáv.) Elvebakk & Bjerke comb. nov.

15DC448A-BE0A-5926-B0DE-72C97686E089

urn:lsid:ipni.org:names:77350950-1

≡ Papaverpyrenaicumsubsp.degenii Urum. & Jáv., Magyar Bot. Lapok 18: 33. 1920. Type: [Bulgaria, Blagoevgrad oblast] In rupestribus alpinus mt. Jel-tepe Perin dag finis turco-bulgare, 2560 m alt., Jun 1909, M. Dimonie (lectotype: WU [barcode WU 0105034]; isolectotypes WU [barcodes WU 0105032; WU 01050333], all designated here) ≡ Papaverdegenii (Urum. & Jáv.) Kuzmanov, Fl. Reipubl. Popularis Bulg. 4: 282. 1970 ≡ Papaveralpinumsubsp.degenii (Urum. & Jáv.) Markgr., Phyton (Horn) 7: 312. 1958.

##### ﻿3.8.5. Oreomecon alpina subsp. ernesti-mayeri

Taxon classificationPlantaeRanunculalesPapaveraceae

(Markgr.) Elvebakk & Bjerke comb. nov.

9B8D3439-2EEC-5818-B4F6-BBB51942EDC6

urn:lsid:ipni.org:names:77350951-1

≡ Papaveralpinumsubsp.ernesti-mayeri Markgr., Phyton (Horn) 7: 312. 1958. Type: [Slovenia] Julische Alpen, Triglav, Staničeva Koča, 1956, *F. Markgraf* (holotype not found) ≡ Papaverernesti-mayeri (Markgr.) Wraber; Proteus (Ljubljana) 44: 238 (1982).

##### ﻿3.8.6. Oreomecon alpina subsp. fatramagnae

Taxon classificationPlantaeRanunculalesPapaveraceae

(Bernát.) Elvebakk & Bjerke comb. nov.

F2F7C122-E006-56BF-B17F-70CD45FFB754

urn:lsid:ipni.org:names:77350952-1

≡ Papavertatricumsubsp.fatraemagnae Bernát., Fl. Slovenska 5(4): 765. 2002. Type: [Slovakia], Vel’ká Fatra (Fatra Magna) in calcareis declivitatum septentrionali-occidentalis montis Ostrá supra l.d. Konský dol incola], ca. 890 m alt., 8 Aug 1999, *D. Bernátová* (holotype: BBZ).

##### ﻿3.8.7. Oreomecon alpina subsp. kerneri

Taxon classificationPlantaeRanunculalesPapaveraceae

(Hayek) Elvebakk & Bjerke comb. nov.

58AEA8FA-8844-5F5A-AC55-10EEE6CEB28B

urn:lsid:ipni.org:names:77350953-1

≡ Papaverkerneri Hayek, Österr. Bot. Z. 53: 170. 1903. Type: [Slovenia] Steiermark, Sanntaler Alpen, bei den Korošicahütte, 1800 m alt., 18 Jul 1900, *A. von Hayek s.n.* (lectotype GB [barcode GB-004 8359], lectotypified by Markgraf [1958a], p. 41 ≡ Papaveralpinumsubsp.kerneri (Hayek) Fedde, Engl. Pflanzenreich IV. 104(40): 375. 1909.

##### ﻿3.8.8. Oreomecon alpina subsp. markgrafiana

Taxon classificationPlantaeRanunculalesPapaveraceae

Elvebakk & Bjerke, nom. et comb. nov.

368B242A-B249-572E-8915-4BC4372642C6

urn:lsid:ipni.org:names:77350954-1

≡ Papaveralpinumsubsp.rhaeticum (Leresche ex Gremli) Nyman ex Markgr., Phyton (Horn) 7: 311. 1958. Type: Switzerland, Oberengadin (not found), nom. illeg. ≡ Papaverrhaeticum Leresche ex Gremli, Gremli, Excursionsfl. Schweiz: 80. 1874, nom. nud. ≡ Papaveralpinumsubsp.rhaeticum (Leresche) Nyman, Consp. Fl. Eur. Suppl. 2: 16. 1889, nom. nud. ≡ Papaverpyrenaicumsubsp.rhaeticum (Leresche) Fedde, in H.G.A. Engler (ed.), Pflanzenr., IV, 104: 372. 1909, nom. nud.

###### Etymology.

Oreomeconalpinasubsp.markgrafiana is named in honour of the very large contributions to the knowledge of the *O.alpina* group made by the German botanist Friedrich Markgraf (1897–1987).

##### ﻿3.8.9. Oreomecon alpina subsp. occidentalis

Taxon classificationPlantaeRanunculalesPapaveraceae

(Markgr.) Elvebakk & Bjerke, comb. et stat. nov.

4B0917F4-98A2-582A-AF60-D8F834FEFA0F

urn:lsid:ipni.org:names:77350955-1

≡ Papaveralpinumsubsp.tatricumvar.occidentale Markgr., Phyton (Horn) 7: 313. 1958. Type: [France] Hochsavoyen, Vergy, *I. Dörfler* (holotype: B [Dörfler Herb. Norm. 5209] ≡ Papaveroccidentale (Markgr.) H.E.Hess & Landolt, Fl. Schweiz Gebiete 3: 778. 1973.

##### ﻿3.8.10. Oreomecon alpina subsp. sendtneri

Taxon classificationPlantaeRanunculalesPapaveraceae

(Kern. ex Hayek) Elvebakk & Bjerke comb. nov.

3BEAA1A9-0764-5B49-9F87-14D4CCB6B767

urn:lsid:ipni.org:names:77350956-1

≡ Papaversendtneri Kern. ex Hayek; Österr. Bot. Z. 53: 406. 1903. Type: Austria, Tirol, Hafelekar bei Innsbruck, *Kerner s.n.* (holotype?: B [barcode B_10_0294933]) ≡ Papaveralpinumsubsp.sendtneri (Kern. ex Hayek) Schinz & Keller; Fl. Schweiz, ed. 3, 1: 223. 1909.

##### ﻿3.8.11. Oreomecon alpina subsp. tatrica

Taxon classificationPlantaeRanunculalesPapaveraceae

(A.Nyár.) Elvebakk & Bjerke nomencl. nov.

98DD7A7B-89C6-5C50-BF0B-759116488C6F

urn:lsid:ipni.org:names:77350957-1

≡ Papaveralpinumsubsp.tatricum A.Nyár., Acta Geobot. Hung. 5: 19. 1942. Type: Karpaten, Hohe Tatra, *A. Nyárády* (lectotype: not found, designated by Markgraf [1958a: 313]) ≡ Papavertatricum (A.Nyár.) Ehrend., Oesterr. Bot. Z. 122: 268. 1973 ≡ Oreomecontatrica (A.Nyár.) Krivenko, Nov. Syst. Pl. Vasc. 54: e06:3. 2023.

##### ﻿3.8.12. Oreomecon alpina subsp. victoris

Taxon classificationPlantaeRanunculalesPapaveraceae

(Škornik & Wraber) Elvebakk & Bjerke comb. nov.

43D4E13C-A7D0-5D03-9A3A-3B9A2766390C

urn:lsid:ipni.org:names:77350958-1

≡ Papavervictoris Škornik & Wraber, Biol. Vestn. 36(3): 82. 1988. Type: Slovenija, Julijske Alpe, in glareosis declitivitatis septentrionalis montis Matajurski vrh, inter montes Črna prst et Rodica, solo calcareo, 1900 m alt., 20 Jul 1981, *M.Krajit & A.Podobnik 9749/3* (holotype: LJU, no. 109984) ≡ Papaveralpinumsubsp.victoris (Škornik & Wraber) Wraber, Hladnikia 10: 42. 1998.

##### ﻿3.8.13. Oreomecon aurantiaca

Taxon classificationPlantaeRanunculalesPapaveraceae

(Loisel.) Elvebakk & Bjerke comb. nov.

BE8D3CD2-B308-5500-BF8D-85D2C60A6CDA

urn:lsid:ipni.org:names:77350959-1

≡ Papaveraurantiacum Loisel., J. Bot. (Desvaux) 2: 340. 1809. Type: [France] Mont Ventoux, *M. Requien* (holotype: not found) ≡ Papaveralpinumvar.aurantiacum (Loisel.) Markgr. Phyton (Horn) 7: 311. 1958 ≡ Papaverpyrenaicumvar.aurantiacum (Loisel.) Dalla Torre, Alpenfl. 173. 1882 ≡ Papaverpyrenaicumsubsp.aurantiacum (Loisel.) Fedde, Beibl. Bot. Jahrb. Syst. 81: 38. 1905.

##### ﻿3.8.14. Oreomecon lapeyrouseana

Taxon classificationPlantaeRanunculalesPapaveraceae

(Gutermann ex Greuter & Burdet) P.P. Ferrer, Taxon 73: 919. 2024

8B28F98B-A5F3-57A2-97C8-EBBE03213A05

≡ Papaverlapeyrouseanum Gutermann ex Greuter & Burdet, Willdenowia 11: 43. 1981. Type: France, *Lapeyrouse s.n.* (lectotype: B [barcode B_10_0294931], lectotypified by Greuter [1981], p. 43) ≡ Papaveralpinumsubsp.lapeyrousianum (Gutermann ex Greuter & Burdet) Kerguélen, Index Synonym. Fl. France (Coll. Patrim. Nat., 8): xv. 1993.
– Papaverlapeyrouseanum Gutermann, Österr. Bot. Z. 122: 268. 1973, nom. illeg.
– Papaveralpinumsubsp.lapeyrousianum (Gutermann) Kadereit, Bot. Jahrb., 112(1): 84. 1990, nom. illeg.
= Papaverpyrenaicumsubsp.suaveolens P.Fourn., Quatre Fl. France 4: 372. 1936. Type: [France] Lin. sommets elevés, fentes des rochers. Mail du Crystal, Cambredases, Pic de Midy, Erezlidtz, Houle Marboré, *Lapeyrouse s.n* [before 1813] (lectotype, TLM, bottom-right individual, lectotype designated by Banfi et al. [2022], p. 69, illustrated by Banfi et al. [2022], p. 70) ≡ Oreomeconalpinasubsp.suaveolens (P.Fourn.) Banfi, Bartolucci, J.-M.Tison & Galasso, Nat. Hist. Sci. 9(1): 69. 2022 ≡ Papaveralpinumsubsp.suaveolens (P.Fourn.) Rändel, Feddes Repert. 84 (9–10): 173. 1974 ≡ Papaveralpinumsubsp.suaveolens O.Bolòs & Vigo, Bull. Inst. Catalana Hist. Nat., Secc. Bot. 38(1): 73. 1974, isonym ≡ Oreomeconsuaveolens (P.Fourn.) Krivenko, Nov. Syst. Pl. Vasc. 54: e06:3. 2023.
– Papaversuaveolens Lapeyr., Hist. Pl. Pyrenées Suppl. 72. 1818, nom. illeg.

##### ﻿3.8.15. Oreomecon lapeyrouseana subsp. lapeyrouseana

Taxon classificationPlantaeRanunculalesPapaveraceae

(Gutermann ex Greuter & Burdet) P.P.Ferrer, Taxon 73: 919. 2024

73670B4D-1722-56BE-876A-26CFE0FA6204

≡ Papaverlapeyrouseanum Gutermann ex Greuter & Burdet, Willdenowia 11: 43. 1981. Type: France, *Lapeyrouse s.n.* (lectotype: B [barcode B_10_0294931], lectotypified by Greuter [1981], p. 43).

##### ﻿3.8.16. Oreomecon lapeyrouseana subsp. endressii

Taxon classificationPlantaeRanunculalesPapaveraceae

(Asch.) Elvebakk & Bjerke comb. nov.

09E374F8-667D-59D7-B94E-ECDF6B501680

urn:lsid:ipni.org:names:77351065-1

≡ Papaversuaveolensvar.endressii Asch., Bot. Zeitung (Berlin) 27: col. 127. 1869. Type: [France] Ceuillade de Nourri, 8400 ft., Aug 1829, Unio itineraria, *P.A.C. Endress* (lectotype: W; isolectotype: JE [barcode JE00018778], designated here) ≡ Papaverlapeyrouseanumsubsp.endressii (Asch.) Greuter & Burdet, Willdenowia 11: 43. 1981 ≡ Papaveralpinumvar.endressii (Asch.) O. Bolòs & Vigo, Butl. Inst. Catalana Hist. Nat., Secc. Bot. 38(1): 73. 1974 ≡ Papaverlapeyrouseanumvar.endressii (Asch.) Rivas Mart., Itinera Geobot., 15: 705. 2002.

##### ﻿4. Meconopsis

Taxon classificationPlantaeRanunculalesPapaveraceae

Vig., Hist. Nat. Pavots: 20. 1814 nom. cons.

90AE4867-052E-571A-9DC8-2381AF8BFAE7

###### Type species

(conserved): *Meconopsisregia* G.Taylor.

###### Notes.

Grey-Wilson (2014) treated 76 species, 20 subspecies and three named hybrids. Since then, new species and revised concepts of existing taxa have been published, most of them within the monocarpic sections *Racemosae* and *Forrestianae*.

Overall, the genus *Meconopsis* currently includes 95 species and 21 subspecies. As shown in Fig. 2, its distribution largely follows the distribution map by Grey-Wilson (2014), but with a small extension into Xinjiang (China) in the western-most part following the distribution map of *Meconopsisaculeata* Royle in the same source. The westernmost species are all rare, with *M.aculeata* Royle as critically endangered in Pakistan (Majid et al. 2015), *M.latifolia* Prain as a local endemic and *M.neglecta* G.Taylor only known from its type collection (Jafri and Qaiser 2011).

##### ﻿4.1. Meconopsis sect. Racemosae

Taxon classificationPlantaeRanunculalesPapaveraceae

C.Y.Wu & H.Chuang, Acta Bot. Yunnan. 2(4): 374. 1980

D19ABD0B-CC3D-5B87-A91F-AE091C48014A

###### Type species.

*Meconopsisracemosa* Maxim.

###### Notes.

In their phylogenetic study, Xiao and Simpson (2015) concluded that species in the strongly bristly sectionRacemosae do not merit classification within the two series proposed by Grey-Wilson (2014), but show a complicated and partly reticulate evolutionary pattern. Therefore, Xiao and Simpson (2015) proposed that these species should be treated as the “*M.horridula* Hook. f. & Thomson species complex” pending further phylogeographic studies. This involves the following additional taxa not accepted at the species level by Grey-Wilson (2014): *M.calciphila* Kingdon-Ward, *M.castanea* H. Ohba, Tosh. Yoshida & H. Sun, *M.pseudohorrida* C.Y.Wu & H.Chuang and *M.rigidiuscula* Kingdon-Ward. Two more species were later described from Bhutan/adjacent India by Yoshida et al. (2016a, b) and one from Sichuan/Yunnan by Yoshida and Sun (2019b). The latter study did not accept all species included in the *M.horridula* species complex by Xiao and Simpson (2015). We conclude that the section comprises 18 species, significantly increasing from the 11 species monographed by Grey-Wilson (2014).

##### ﻿4.2. Meconopsis sect. Forrestianae

Taxon classificationPlantaeRanunculalesPapaveraceae

C.Y.Wu & H.Chuang, Acta Bot. Yunnan. 2 (4) 375. 1980

29E0E064-50CF-5A5D-8060-C64EE4077D1B

###### Type species.

*Meconopsisforrestii* Prain.

###### Notes.

Meconopsislancifoliasubsp.lepida (Prain) Grey-Wilson was raised to the species level by Yoshida and Sun (2017). Yoshida and Sun (2018) described four new species in the Forrestianaesectionand another four were described by Yoshida and Sun (2019a). They also positioned the species *M.pleurogyna* W.T.Wang (Wang 2019) here, reduced *M.sinomaculata* Grey-Wilson and *M.xiangchenensis* R.Li & Z.L.Dao to variety level and described two more subspecies of *M.lancifolia* (Franch.) Franch. ex Prain. The monograph by Yoshida and Sun (2019a) concluded that the section includes 15 species.

##### ﻿4.3. Meconopsis sect. Impeditae

Taxon classificationPlantaeRanunculalesPapaveraceae

Grey-Wilson, Gen. Meconopsis: 46. 2014

B268F5AF-8371-5CF5-A257-07CD6D3239A3

###### Type species.

*Meconopsisimpedita* Prain.

###### Notes.

SectionImpeditae Grey-Wilson (Grey-Wilson 2014) partly overlaps with sectionForrestianae as monographed later by Yoshida and Sun (2019a) and referred to above. Two new species of *Impeditae*, *M.angustipetala* W.T.Wang and *M.brachynema* W.T.Wang, were described from China (Wang 2019).

##### ﻿4.4. Meconopsis sect. Grandes

Taxon classificationPlantaeRanunculalesPapaveraceae

(Prain.) Fedde, Engl., Pflanzenr. 4, 104: 262. 1909

31BC4F34-4779-53F1-B74E-FC6C74456B3A

###### Type species.

*Meconopsisgrandis* Prain.

###### Notes.

Grey-Wilson (2014) included four conspicuously yellow species in SectionGrandes (Prain) Fedde. Subsequently, two additional species were added to the section; one is the new species, *M.wanbaensis* Tosh. Yoshida (Yoshida 2019), the other is the recombined *M.uniflora* (C.Y.Wu & H.Chuang) Tosh. Yoshida et al. (Yoshida et al. 2019). Whereas almost all *Meconopsis* species described after the publication of the monograph by Grey-Wilson (2014) are monocarpic and, in the case of the *Forrestianae* and *Impeditae*, rather small plants, one large and blue perennial poppy has also been described. This is *M.gakyidiana* Tosh. Yoshida et al., the famous blue poppy of Bhutan, which is also the country’s national flower (Yoshida et al. 2016b). It has remained in cultivation since George Forrest’s introduction in the 1930s, partly under various cultivar names (Grey-Wilson 2017), partly as M.grandissubsp.orientalis Grey-Wilson, until the latter was raised to species level under the name referring to Bhutan’s “*gakyid*” concept of national happiness.

##### ﻿5. Afropapaver

Taxon classificationPlantaeRanunculalesPapaveraceae

(Elkan) Elvebakk & Bjerke, nom. et stat. nov.

4BC68EA4-EA58-5BE3-B19E-089B15DBFD95

urn:lsid:ipni.org:names:77350960-1

≡ Papaversect.Horrida Elkan, *Tent. Monogr. Papav.* 32. 1839. Type species: Papaveraculeatum Thunb. [≡ Afropapaveraculeatum (Thunb.) Elvebakk & Bjerke].

##### ﻿5.1. Afropapaver aculeatum

Taxon classificationPlantaeRanunculalesPapaveraceae

(Thunb.) Elvebakk & Bjerke comb. nov.

4A77BFF0-16F0-592D-AD25-CA6C1829CF33

urn:lsid:ipni.org:names:77350961-1

≡ Papaveraculeatum Thunb., *Prodr. Pl. Cap.* 2: 92. 1800. Type: e Cap. b. Spei (“eastern Cape of Good Hope”), *C.P. Thunberg*, UPS-THUNB (V-106276).
= Papaverhorridum DC., Syst. Nat. 2: 79. 1821. Type: Hab. in Nova-Hollandia, *Caley* (holotype: BM).
= Papavergariepinum Burch. ex DC., Trav. S. Afr. 1: 318. 1822. Type: Africa extratropica ad ripas fluminis Gariep seu Orange-River, *Burchell 1633* (holotype: K).

###### Notes.

This is a new, monospecific genus. The basionym name “*horrida*” is an adjective in the plural. There are examples of adjectives used as nouns in the names of genera. However, according to the recommendations in the Code (20A.1.[f, g]; Turland et al. (2018)), one should avoid adjectives as nouns and one must not use the epithet or derived form of the epithet of one of the species of the genus in question. The present synonym, *Papaverhorridum*, is based on anthropogenically induced material from Australia (Kadereit 1988c) and would have priority in case Australian material is lifted to the species level by future studies. Therefore, a replacement name is a preferred alternative. Papaversect.Horrida was monographed by Kadereit (1988c), including synonyms and types.

###### Etymology.

*Afropapaver* refers to its relationship to *Papaver* and its strongly isolated occurrence in southernmost Africa (Kadereit 1988c).

###### Distribution.

**Figure 4.**
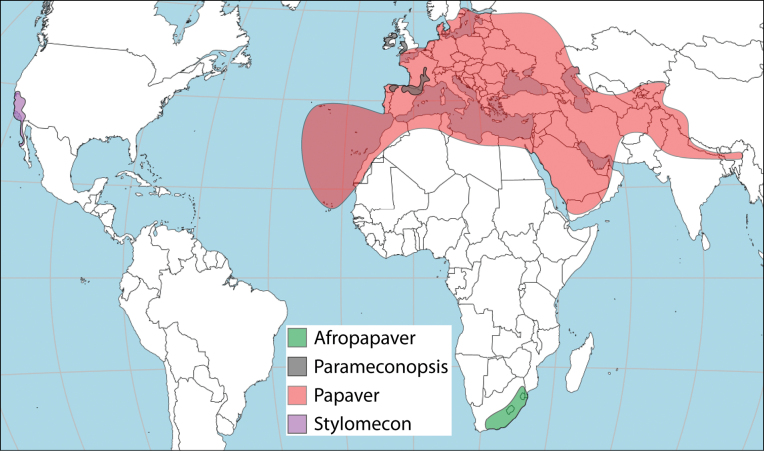
World distribution of the genera *Afropapaver* (green), *Papaver* (red), *Parameconopsis* (grey) and *Stylomecon* (violet). See Table 1 for species number per genus.

Its distribution, as shown here in Fig. 4, is based on Kadereit (1988c), who concluded that the species is an early human introduction to Australia and is also synanthropic in Namibia.

##### ﻿6. Stylomecon

Taxon classificationPlantaeRanunculalesPapaveraceae

G.Taylor, J. Bot. 68: 140. 1930

4FF0EB97-B7E9-5934-841C-075BEF8E0B91

###### Type species.

*Stylomeconheterophylla* (Benth.) G.Taylor.

###### Notes.

*Stylomeconheterophylla* (Benth.) G.Taylor and the species known as *Papavercalifornicum* A.Gray are endemic to California and adjacent parts of Mexico, where they are grossly disjunct as they are the only representatives of the mostly Eurasian clade of Papavereae in America. Samples from this group of species diverged from the remaining samples of *Papaver* and *Parameconopsis* at ca. 19–20 Ma according to the phylogenies by Valtueña et al. (2012) and Xie et al. (2014) and a similar phylogenetic position was shown by Liu et al. (2014). Catania et al. (2022) concluded that *Papavercalifornicum* was the earliest branching species from a common ancestor in the *Papaver* lineage, which had a gene fusion event basal for the further synthesis of the morphinan group of alkaloids, a divergence dated at 16.8 Ma.

According to these phylogenies, these two species definitively should be congeneric. Kadereit and Baldwin (2011) dealt with their morphology, ecology and distribution in detail and showed differences in flower and capsule morphology. They concluded that the style in *S.heterophylla* probably evolved independently from other lineages and a structure similar to the stigmatic disc of *P.californicum* and they treated both species within a broad definition of *Papaver*.

Given that these two Californian-Mexican species have an old evolutionary history, a similar phylogeny and distribution and a diverging style which probably evolved relatively recently within its lineage, the clade is best treated as a separate genus. The name *Stylomecon* is available and a new combination is needed for the species known as *P.californicum*.

*Stylomeconheterophylla* was briefly described as *Meconopsisheterophylla* Benth. by Bentham (1835), who also described *Meconopsiscrassifolia* Benth. Both type specimens shown by JSTOR Global Plants (2023) carry the label information “Nova California, 1833, Douglas’. According to Bentham (1835), David Douglas travelled from present-day Oregon to then Mexican California and carried out botany studies in the surroundings of Monterey during the years 1831 and 1832. Then he travelled to what is now named Hawaii and dispatched his plants by ship to England before he returned to Oregon. The year of the label should, therefore, refer to the year of the plants’ arrival in England and not the year of collecting. According to Brentham (1835), cultivation attempts failed and the plants preserved are, therefore, those collected by Douglas. Although classified as isotypes by JSTOR Global Plants (2023), below, we list these as holotypes in the absence of known duplicates.

Steudel (1841) included both *Meconopsisheterophylla* and *Meconopsiscrassifolia* within a widely defined genus *Stylophorum* Nutt., including two from California, two others from northern America, one from Europe and two from Nepal. In a flora of the San Francisco area, Greene (1894) accepted both names as species of *Papaver* together with *P.californicum* and *P.lemmonii* Greene. Much later, Kadereit (1988a) and Kadereit and Baldwin (2011) accepted only *Papavercalifornicum* and *Stylomeconheterophylla* and did not present interpretations of the name *Meconopsiscrassifolia*. Grey-Wilson (2014), however, considered the latter to be a synonym of the very different *Stylophorumdiphyllum* (Michx.) Nutt. distributed on the opposite side of the North American continent, an interpretation followed by POWO (2023).

Kadereit and Baldwin (2011) explained and illustrated the leaves of *Stylomeconheterophylla* to be very different from those of *Papavercalifornicum*, the latter being strikingly heterophyllous. The holotypes of *Meconopsisheterophylla* and *M.crassifolia* illustrated by JSTOR Global Plants (2023) differ in the same way and also match the diagnoses by Bentham (1835). We conclude that *Meconopsiscrassifolia* and *Papavercalifornicum* are synonyms and that the former holds priority.

#### ﻿Accepted taxa

##### ﻿6.1. Stylomecon crassifolia

Taxon classificationPlantaeRanunculalesPapaveraceae

(Benth.) Elvebakk & Bjerke comb. nov.

C8010233-5EAE-53FD-B659-473354068481

urn:lsid:ipni.org:names:77350962-1

≡ Meconopsiscrassifolia Benth., Trans. Hort. Soc. London, Ser. 2, 1: 408. 1835: Type: USA, Nova California,1833, *D. Douglas s.n.* (holotype: BM) ≡ Stylophorumcrassifolium (Benth.) Steud., Nomencl. Bot. ed. 2, 2: 650. 1841 ≡ Papavercrassifolium (Benth.) Greene, Man. Bot. San Francisco: 9. 1894 ≡ Papaverheterophyllumvar.crassifolium (Benth.) Jeps., Fl. W. California: 209. 1901.
= Papavercalifornicum A.Gray; *Proc. Ameri. Acad. Arts* 22: 323. 1887. Type: USA: California, Santa Inez Mountains, *J. Spence s.n.* (holotype: GH), syn. nov.
= Papaverlemmonii Greene, Pittonia 1: 168. Type: USA: California, San Luis Obispo County, 1887, *J.G. Lemmon s.n.* (holotype: NDG).

##### ﻿6.2. Stylomecon heterophylla

Taxon classificationPlantaeRanunculalesPapaveraceae

(Bentham) G. Taylor, J. Bot. 68: 140. 1930

0021C78D-4282-5E01-9C02-2BEBD4D087B2

≡ Meconopsisheterophylla Benth., Trans. Hort. Soc. London, Ser. 2, 1: 408. 1835: Type: USA, Nova California,1833, *D. Douglas s.n.* (holotype: BM) ≡ Stylophorumheterophyllum (Benth.) Steud. Nomencl. Bot. red. 2, 2: 650. 1841 ≡ Papaverheterophyllum (Benth.) Greene, Pittonia 1: 168. 1888.

##### ﻿7. Papaver

Taxon classificationPlantaeRanunculalesPapaveraceae

L., Sp. Pl. 1: 506. 1753

D1F5E459-FAB0-5BFA-9E13-E6FFEC01AA74

###### Type species.

*Papaversomniferum* L.

###### Notes.

The sections of *Papaver* s.str. have been thoroughly dealt with in a series of monographs; see summary below. The sections have also been compared, for example, by Kadereit (1988b) and phylogenetically explored, for example, by Carolan et al. (2006).

As circumscribed and reviewed here, the genus *Papaver* includes 59 species and 14 subspecies and most of the changes, compared with the cited reference studies, have taken place in the large sectionRhoeadium Spach. The distribution of the genus is shown in Fig. 4, where anthropogenic occurrences are excluded. The genus ranges from the endemic species *P.gorgoneum* on the Cape Verde Islands (Kadereit and Lobin 1990) through Central Europe and the Mediterranean area. Latitudinally, it occurs from Central European Russia and the western Tian Shan southwards into the Arabian Peninsula. Eastwards, the genus reaches as far east in Central Asia as Kyrgyzstan with the species *P.laevigatum* M.Bieb. (Sennikov and Tojibaev 2021) and *P.macrostomum* extends eastwards to Assam along the foothills of the Himalayas (Fig. 4).

##### ﻿7.1. Papaver L. sect. Papaver

Taxon classificationPlantaeRanunculalesPapaveraceae

, Sp. Pl. 1: 506. 1753

8B9C2136-33C8-5393-84CE-8F0414B8721E

###### Type species.

*Papaversomniferum* L.

###### Notes.

This section includes four species and one subspecies, according to the monograph by Kadereit (1986b). However, the section was not monophyletic, according to Carolan et al. (2006). Based on seed morphology, Jesus et al. (2023) recently showed archaeological evidence of the cultivation of *Papaversomniferum* in Europe dating back about seven millennia. During the first millennia, seeds indicating the wild and weedy morphotype were mostly treated as P.somniferumsubsp.setigerum (DC.) Arcang., were dominant and, after a transition period, the cultivated morphotype became dominant about 3000 years ago. This selection towards larger seeds appears from these data to be primarily a result of human domestication and not one of vicariant evolution. The latter is a major criterion applied for the subspecies category by POWO (2023). However, Hong et al. (2022) convincingly proved that the wild type is genetically clearly distinct and in a sister group position to *P.somniferum*, coupled with the morphological difference shown by Kadereit (1986b). As indicated by Liu et al. (2020), it is unlikely that the tetraploid wild type is the progenitor of the diploid *P.somniferum*. We, therefore, interpret the wild-growing taxon as the separate species *P.setigerum* DC.

##### ﻿7.2. Papaver sect. Carinatae

Taxon classificationPlantaeRanunculalesPapaveraceae

Fedde, Engler (ed.) Pflanzenr. 40 (4; 104): 334. 1909

D5D67726-3FE7-59DB-A19B-7EDFC5E2075F

###### Type species.

*Papavermacrostomum* Boiss. & Huet, Boiss. Fl. Orient. 1: 115. 1867.

###### Notes.

Includes a single species, *P.macrostomum* Boiss. & A.Huet, with a dehiscing capsule disc, which is an exclusive character according to Kadereit (1987), who only included four varieties. POWO (2023) also accepted *P.halophilum* (Fedde) Cullen and *P.piptostigma* Bien. ex Fedde. However, they were treated as synonyms of *P.macrostomum* by Kadereit (1987) and Tavakkoli and Assadi (2016).

##### ﻿7.3. Papaver sect. Macrantha

Taxon classificationPlantaeRanunculalesPapaveraceae

Elkan, Tent. Monogr. Papaver 19: 1839

654386D1-7191-5A62-8F62-C585E23DE393

###### Type species.

*Papaverorientale* L., Sp. pl. 508. 1753.

###### Notes.

This section includes the famous and perennial species *Papaverorientale* L. and two more species according to most studies, including the recent monographic treatments by Lack (2019a, b), who argued convincingly why the name *P.pseudo-orientale* (Fedde) Medw. should be conserved. Solomon et al. (2014) accepted *P.lasiothrix* Fedde as an additional species. However, this had been rejected in a study on Iranian species, which treated it as a synonym of *P.bracteatum* Lindl. (Tavakkoli and Assadi 2013), a conclusion also reached by Lack (2019a) and POWO (2023). The new species *Papaveryilderimlii* Ertekin was described from a single site in the province of Siirt in Türkiye as similar to *P.lasiothrix*, except for smaller and globose capsules (Yıldırımlı and Ertekin 2008). *Papaveryilderimlii* was accepted by POWO (2023), but not treated by Lack (2019 a, b) nor in other recent studies dealing with this complex. It is treated here as a synonym of *P.bracteatum*. The species concept in the section was also confirmed by a study on morphology, phylogeny and chemistry in Türkiye (Parmaksız and Özcan 2011). In contrast, another Turkish phylogenetic study which applied ISSR markers (Gürkök et al. 2013) did not clearly sort out the given sample names over the major clades.

##### ﻿7.4. Papaver sect. Rhoeadium

Taxon classificationPlantaeRanunculalesPapaveraceae

Spach, Hist. Nat. Veg. Phan. 7: 16. 1839

BB800424-DF38-56E5-BFA2-979C20DF3359

###### Type species.

*Papaversegetale* Schimp. & Spenn., Fl. Friburg. 3: 1829 [= *Papaverrhoeas* L.].

###### Notes.

This section was monographed by Kadereit (1989), who accepted 16 species and five subspecies, whereas the Cape Verdean *P.gorgoneum* Coutinho with one additional subspecies was added by Kadereit and Lobin (1990). The following changes indicated by POWO (2023) and Hassler (2023b) are accepted here: *Papaverguerlekense* Stapf is reduced to synonymy of *P.rhoeas* L., *P.stylatum* Boiss. is reduced to synonymy of *P.umbonatum* Boiss. and three subspecies of *P.dubium* L. are raised to species level as *P.glabrum* Royle, *P.lecoqii* Lamotte and *P.laevigatum* M. Bieb., respectively.

The following taxa were not accepted by Kadereit (1989) or were described subsequently. *Papaverpostii* Fedde, treated as a synonym of *P.rhoeas* L. by Kadereit (1989), was accepted as a deviating perennial member of *Rhoeadium* by Cullen (1965) and later also accepted by POWO (2023). In a study from Cyprus, Aghababyan et al. (2011) compared *P.postii* Fedde with the new species *P.paphium* M.V.Agab. et al. and *P.cyprium* (Chrtek & B.Slavik) M.V.Agab. et al., the latter being a taxon originally described as a subspecies of *P.rhoeas*. These taxa are all accepted by POWO (2023) and Hassler (2023b) and are also accepted here.

Some rare species were dealt with in a study on the Red-listed species of Caucasus in a broad sense by a joint effort of botanists from Türkiye, Georgia, Armenia, Azerbaijan, Russia and Iran (Solomon et al. 2014). The *Papaver* species agreed on by these authors are, with one exception, accepted here. Their treatment includes three endemic species from Armenia described by Aghababyan and Fragman-Sapir (2007), two within this section. *Papaverroseolum* M.V.Agab. & Fragman was listed as endangered and *P.gabrielianae* M.V.Agab. as vulnerable (Solomon et al. 2014). *Papaverschelkovnikovii* N.Busch from Azerbaijan was listed as endangered by Solomon et al. (2014) and included within the top 50 national conservation priorities of this country. It is known from two localities and cited as endemic, although POWO (2023) cited it to have a wider distribution. In addition, Aghababyan (2013) described *P.gorovanicum* M.V.Agab. as a local endemic from sandy soils near the village of Gorovan in the Ararat Region of Armenia.

*Papaveralbiflorum* (Elkan) Pacz., previously included as a variety within *P.dubium* by Kadereit (1989), was accepted by POWO (2023), who treated *P.paczoskii* Mikheev as a synonym. *Papaverconfine* Jord., from the same complex in Europe, was also accepted by POWO (2023) and by Hassler (2023b). Mikheev (1993), who reviewed the Papaveraceae flora of the Russian Caucasus, reduced his *P.alberti* Mikheev to a synonym of *P.stevenianum* Mikheev, which later was treated as P.dubiumsubsp.stevenianum (Mikheev) Kubát & Šipošová, an alternative followed here. Solomon et al. (2014, as *P.alberti*) treated the taxon as Nearly Threatened (NT). Mikheev (1999) described the new species *P.maschukense* Mikheev from the foot of the Maschuk Mountain in the Russian part of Caucasus.

Tavakkoli and Assadi (2016) monographed *Papaver* in Iran, but did not mention the Iranian species *P.pasquieri* Dubuis & Faurel, which is accepted by POWO (2023) and Hassler (2023b). Tavakkoli and Assadi (2016) also maintained *P.bipinnatum* C.A.Mey. as a synonym of *P.arenarium* M. Bieb. and treated *P.lacerum* as a synonym of *P.commutatum* Fisch., C.A.Mey. & Trautv. instead of *P.laevigatum*. Dar et al. (2010) described two new local species from near Srinagar in north-westernmost India and *P.kachroianum* Tabinda, Dar & Naqshi was only described from its holotype. Its etymology was explained as commemorating the botanist P. Kachroo and the orthography of the epithet is corrected to “*kachrooianum*” here according to the Code, Art. 60.8; Turland et al. (2018). *Papaverpamporicum* Tabinda, Dar & Naqshi was described from cultivated specimens originating from saffron fields in Pampore in the same area. *Papaverstewartianum* Jafri & Qaiser was described from Pakistan, based on the type specimen from grain fields at Campbellpore. It was described as a possible hybrid, but has been maintained by Jafri and Qaiser (2011) and is accepted by POWO (2023) and Hassler (2023b).

*Papavermaireii* Batt. and *P.malviflorum* from North Africa, both previously included within *P.dubium*, were accepted by POWO (2023) and Hassler (2023b), the former referring to flora treatments. The conclusion is that sectionRhoediana includes 34 species and three subspecies.

##### ﻿7.5. Papaver sect. Meconidium

Taxon classificationPlantaeRanunculalesPapaveraceae

Spach, Hist. Nat. Veg. Phan. 7: 21. 1839

550DBD14-5886-5B95-8090-B3F14C498E05

###### Type species.

*Papaverarmeniacum* (L.) DC. Prodr. 2: 79. 1821. [≡ *Argemonearmeniaca* L., Sp. pl.: 509. 1753].

###### Notes.

Kadereit (1993), in his monograph for the section of orange-flowered, biennial species with valvate capsules, accepted four species in addition to five subspecies. In addition, Tavakkoli (2012) and POWO (2023) accepted *Papaveracrochaetum* Bornm. ex Fedde. The species *P.sjunicicum* M.V.Agab. was described by Aghababyan & Fragman-Sapir (2007) from altitudes above 3200 m in the Zangezura area of Armenia. It was listed as a data-deficient species from Armenia in the Red List treatment for the widely-defined Caucasus Region (Solomon et al. 2014). POWO (2023) considers this species a synonym of P.armeniacumsubsp.armeniacum (L.) DC, which is a taxon with several other synonyms that are still widely in use, for example, *P.caucasicum* M. Bieb., *P.fugax* Poir. and *P.triniifolium* Boiss. Aghababyan & Fragman-Sapir (2007) treated *Papaversjunicicum* as different from *P.zangezuricum* Mikheev, another species from 3500 m alt. in the same area of Armenia, see Mikheev (1993). The latter species is accepted by POWO (2023) and both are accepted here.

*Papavershepardii* Post ex Dinsm. from southern Türkiye near the border of NW Syria was considered a Critically Endangered (CR) species by Cullen (1965). It is probably identical to P.persicumssp.tauricola (Boiss.) Kadereit. *Papavershepardii* was not mentioned by Kadereit (1993). It was considered unresolved by Aghababyan (2011b) and is not accepted here, although it was so by POWO (2023). Our summary thus shows that this section is comprised of seven species and five subspecies.

##### ﻿7.6. Papaver sect. Pilosa

Taxon classificationPlantaeRanunculalesPapaveraceae

Prantl, Engler & Prantl, Nat. Pflanzenfam. 3, 2: 142. 1889

B575D3E0-21F4-531A-B6A2-25D4D5223998

###### Type species.

Sibt. & Smith, Fl. Graeca Prodr. 1: 360. 1973.

###### Notes.

This section consists of a single species, *Papaverpilosum* Sm., with four subspecies, all distributed in western parts of Türkiye (Kadereit 1996).

##### ﻿7.7. Papaver sect. Pseudopilosa

Taxon classificationPlantaeRanunculalesPapaveraceae

M.Popov ex Günther, Flora 164: 436. 1975

9BB0490E-07C6-5A4C-AFCF-97871D9C5C99

###### Type species.

*Papaverrupifragum* Boiss. & Reut., Pugill. Pl. Afr. Bot. Hispan.: 6. 1852.

###### Notes.

This section was monographed as including three species with two additional subspecies by Kadereit (1996), showing a wide disjunction between the species pair *P.atlanticum* (Ball) Coss. and *P.rupifragum* Boiss & Reut. in Morocco and southern Spain and *P.lateritium* K. Koch in east Türkiye and Transcaucasus. In a more recent monograph, Aghababyan (2009) also accepted *P.oreophilum* Rupr., regarded as an endemic of central parts of the main Caucasus mountain chain. He also accepted *P.monanthum* Trautv. as an endemic distributed from east Türkiye through the Trans-Caucasian mountains from southern Georgia to northern Armenia, whereas *P.lateritium* was considered a local endemic of east Türkiye.

Furthermore, Aghababyan (2009) treated *P.lisae* N.Busch, a local endemic of the Russian Republic of Kabardino-Balkaria on the northern side of the central main Caucasus. Popov (1937) considered this species problematic and could not easily assign it to any section. The same conclusion was reached by Kadereit et al. (1997), who cited that it had been proposed that a separate section be formed by an unpublished study. Aghababyan (2009), on the other hand, considered it to be surprisingly similar to the Moroccan species *P.atlanticum*. It was mapped and treated as Endangered (EN) by Solomon et al. (2014). Grey-Wilson (2023) recently transferred *P.lisae* to *Oreomecon*. However, we follow Aghababyan (2009) and accept it as a species within sectionPseudopilosa.

*Papavertalyshense* Grossh. was considered to be a dubious species by Aghababyan (2009), but was mapped from a single locality in Azerbaijan and regarded as endangered by Solomon et al. (2014). They listed it amongst the country’s top 50 national conservation priorities. The species is accepted by Mikheev (1993), POWO (2023) and Hassler (2023b). Overall, we conclude that sectionPseudopilosae includes eight species and two subspecies.

##### ﻿8. Parameconopsis

Taxon classificationPlantaeRanunculalesPapaveraceae

Grey-Wilson, Gen. Meconopsis: 367. 2014

476FA82A-85B0-5775-A800-1CB81142779A

###### Type species.

*Parameconopsiscambrica* (L.) Grey-Wilson.

###### Notes.

This monospecific genus includes *Parameconopsiscambrica* (L.) Grey-Wilson, see treatment by Grey-Wilson (2014). The distribution map in Fig. 4 is based on Valtueña et al. (2012).

##### ﻿8.1. Parameconopsis cambrica

Taxon classificationPlantaeRanunculalesPapaveraceae

(L.) Grey-Wilson, Gen. Meconopsis: 367. 2014

04916B11-7FF1-55AC-BF02-7A371B0A76BC

≡ Papavercambricum L., Sp. Pl.: 508. 1753. Type: In Pyrenaeis. Herb. Burser IX: 45 (lectotype: UPS-BURSER, designated by Ferrer-Gallego [2015], p. 208) ≡ Meconopsiscambrica (L.) Vig., Hist. Nat. Pavots: 48. 1814.

## ﻿Discussion

The present treatment of Papavereae is based on a review of literature on the taxonomy of what Carolan et al. (2006) referred to as the Old-World clade of Papaveroideae, including the genera *Meconopsis*, *Papaver*, *Roemeria* and *Stylomecon* and comprising approximately 130 species of the two former, three of *Roemeria* and one of *Stylomecon*. Based on a set of defined reference studies and discussed deviations from these, the present study accepts eight genera, a total of 246 species and 61 subspecies, many evidently in need of further studies. Three of the genera are large and the number of accepted species and subspecies is 95 + 21 for *Meconopsis*, 68 + 29 for *Oreomecon* and 59 + 14 for *Papaver*, the latter surprisingly being only the third largest genus of the group.

### ﻿Generic concepts and phylogenies within Papaveraceae

The arrangements of the presently defined genera were quite similar in the large, mainly ITS-based phylogenies by Carolan et al. (2006), Kadereit et al. (2011), Valtueña et al. (2012), Liu et al. (2014) and Xie et al. (2014). Three of the studies also included chronologies and they indicated that the Eurasian group within Papavereae, including the eight genera accepted in the present study, diverged from the American clade during the early Tertiary, at ca. 52 Ma according to a calibration with the oldest Papaveraceae fossil by Kadereit et al. (2011). This divergence time was recently estimated to be 81.5 Ma (Peng et al. 2023). Four of the studies above showed *Cathcartia* to have the earliest divergence, a pattern confirmed by Peng et al. (2023). Kadereit et al. (2011) dated this divergence at ca. 38 Ma followed by a split at ca. 28 Ma of the clade including *Roemeria*, *Meconopsis* and *Oreomecon* vs. a clade including further divergences, first of *Afropapaver*, then *Stylomecon* and finally a split-off of *Parameconopsis* and *Papaver* at 12.7 (6.6–19.0) Ma, see Fig. 1.

As compared with Kadereit et al. (2011), Valtueña et al. (2012) added more samples of *Parameconopsiscambrica*, but their phylogeny is very similar, except for the oldest dichotomies where *Cathcartia* was lacking. Xie et al. (2014) estimated the divergence of *Meconopsis* and *Oreomecon* at 16.8 Ma vs. ca. 23.5 Ma by Kadereit et al. (2011). Xie et al. (2014) only included a single sample of the presently defined genus *Roemeria*. However, *Meconopsis* was their focal point compared to *Parameconopsis* by Kadereit et al. (2011) and Valtueña et al. (2012).

*Afropapaver* and *Stylomecon* are both represented by only one or two sequences each in the cited phylogenies on this group and further phylogenetic studies with more markers are evidently needed, cf. the exploration of new markers by Liu et al. (2020). However, their patterns clearly indicate two early diverging genera. The type material of the *Afropapaver* synonym *Papaverhorridum* DC. (POWO 2023) was collected by George *Caley* in Australia in 1803, just after the very onset of British colonisation. However, we follow Kadereit (1988b) in accepting *Afropapaveraculeatum* as a remarkably early human introduction into Australia. In addition to its isolated distribution, the monotypic genus *Afropapaver* has several distinct characters, including a spiny indumentum, yellow filaments and anthers, 2n = 11 and a racemose inflorescence (Kadereit 1988b). His description does not explicitly include the dehiscence structure, but it is illustrated as short-valvate. Thus, the combination of genetics, morphology, karyology and distribution supports the status of *Afropapaver* as a separate genus, which evolved most likely after a long-distance dispersal event, possibly > 20 Ma ago.

Thompson (2005) also cited several other examples of generic pairs between the Cape and the Mediterranean floras thought to have ancient origins, modified here as *Moraea* Mill. with neighbouring genera vs. *Iris* Tourn. ex L. (Goldblatt et al. 2002), *Lobostemon* Lehm. vs. *Echium* Tourn. ex L. (Hilger and Bohle 2000) and *Passerina* L. (Bredenkamp and van Wyk 2006) vs. *Daphne* Tourn. ex L. Cowling and Holmes (1992) concluded from a study in the Cape Region that regional or local endemics over-represented in several genera had a particular biological profile. They were primarily non-sprouting dwarf shrubs with ant-dispersal of seeds over short distances in combination with soil-stored seed banks or they had microsymbiont-mediated nutrient uptake. None of these traits is valid for the monotypic genus *Afropapaver*.

The two species in *Stylomecon* have been taxonomically challenging as their capsules have such contrasting morphology and *S.californica* was positioned in the monotypic section Papaversect.Californica Kadereit by Kadereit (1988a). The section name has been corrected to plural here according to the Code (Art. 21.2; Turland et al. (2018)). The first study treating these as sister species based on molecular data was the one by Kadereit et al. (1997), later confirmed by Carolan et al. (2006) and Kadereit et al. (2011) and supported by a study on morphology and ecology by Kadereit and Baldwin (2011).

Catania et al. (2022) indicated, based on a whole-genome analysis, that the gene fusing event basal for the synthesis of morphine-types of alkaloids had a monophyletic origin in *Stylomeconcalifornica* and it was dated at 16.8–24.1 Ma. It could have evolved in the *Stylomecon* lineage or its ancestor, but posterior to the divergence dated at 24 Ma of a branch including *Roemeria* and *Oreomecon*, where this gene fusion had not occurred. The most likely hypothesis is that it has taken place in the *Stylomecon* ancestor lineage prior to the establishment of the taxon in California/Mexico. Otherwise, one would need to postulate a re-migration into Eurasia, allowing for later diversification of this chemosyndrome. The enigmatic distribution of *Stylomecon* could be explained by an extreme long-distance dispersal event from western Eurasia, where its closest relatives occur, including the likely source area of *Afropapaver*, as indicated by Kadereit (1988b). Kadereit and Baldwin (2011) stated that there is no fossil evidence for a postulated wide distribution of the ancestral lineage in North America, which would have reduced the migration distance needed from western Eurasia. A third hypothesis is a less dramatic dispersal from East Asia to California. However, this would require postulating an extensive lineage extinction in eastern and central Eurasia. Thus, the early members of this group only survived as *Afropapaver* and *Stylomecon* and not in Eurasia, where only later divergences were present.

*Parameconopsis* and *Papaver* s.str. also diverged early and the former survived the Quarternary as a Tertiary relict species in several disjunct areas in western Europe, as shown convincingly by Valtueña et al. (2012). It is also a sister group to *Papaver* s.str. in the phylogenies by Carolan et al. (2006), Liu et al. (2014) and Xie et al. (2014). This leaves *Papaver* and *Parameconopsis* as the most recently evolved sister group among these genera. *Parameconopsis* also contains yellow flower pigments known as nudicaulins. They are absent from *Papaver*, but different nudicaulins are present in intensely yellow or orange flowers of *Oreomecon* (Tatsis et al. 2013).

In the phylogram by Kadereit et al. (2011), where *Papaver* as defined in the present study is denoted as “*Papaver* s.str.”, the earliest divergence amongst its numerous sections is sectionPseudopilosa, based on analyses of the Moroccan/Spanish species pair *P.atlanticum* and *P.rupifragum*. Together with the present distribution of *Parameconopsis*, this could indicate that *Papaver* arose in the western part of the Mediterranean. SectionPseudopilosa has a remarkable disjunction between Morocco and Türkiye (Kadereit 1996). The remaining sections of *Papaver* all have their diversity centres in the latter country and adjacent areas and an evolutionary origin here has been a prevailing view (Kadereit 1998).

Styles are very short or obsolete in the oldest genus, *Cathcartia* (Grey-Wilson 2014) and have a different ontogeny and evolutionary history in *Parameconopsis* as compared to *Meconopsis* (Kadereit and Erbar 2011). Carolan et al. (2006) consider valvate capsules to be the most ancient character in Eurasian *Papaverae*. The valvate capsules of *Cathcartia* can be interpreted as a synapomorphy being maintained within all dichotomies involving the evolution of the genera *Meconopsis*, *Oreomecon*, *Afropapaver*, *Stylomecon* and *Parameconopsis*, but lost in *Stylomeconheterophyllum*. Alternatively, the capsule dehiscence in the latter can be interpreted as short-valved from the illustrations in Kadereit and Baldwin (2011). Outside of these six genera within Old-World *Papaverae*, valvate capsules occur only in Papaversect.Meconidium (Kadereit 1993). As indicated by Carolan et al. (2006), this section represents the most recent divergence within *Papaver* and its acquisition of valvate capsules evidently occurred as a separate evolutionary event.

Poricidal capsule dehiscence occurs in *Roemeria* and *Papaver* and probably represents separate evolutionary events. As opposed to the long-valvate genera *Cathcartia*, *Meconopsis* and *Parameconopsis* from mesic habitats, the genera above occur in open, arid areas, sharing the evolutionary advantages of poricidal capsule dehiscence for seed dispersal in wind-exposed and open habitats. The seeds would be retained within the capsules during calm periods and dispersed predominantly during windy episodes when they travel further.

During the late Miocene, global cooling was coupled with increasing aridity and ecosystem changes (Herbert et al. 2016). Pound et al. (2011) showed that, during the Tortonian Miocene, 11.6–7.25 Ma, warm-temperate, wet forests covered central and southern parts of Europe, except for southern and western parts of the Iberian Peninsula. Türkiye, by contrast, had broadleaved temperate savannahs with large extensions further east. The success of the genus *Papaver*, as opposed to its three small and geographically isolated neighbouring genera, might be its adaptations to the expanding arid ecosystems of the east Mediterranean. However, its evolutionary origin could hypothetically have been further west.

*Oreomecon* is also of particular interest regarding capsule morphology. As shown by Kadereit (1988a), their capsules have valvate dehiscence. However, they are so short-valved that they share the evolutionary adaptations to open areas with the genera with true poricidal capsules. The scapose pedicels of *Oreomecon* species, rigid when capsules are mature, also add to an adaptation to harsh Arctic and Alpine habitats. Seeds will be dispersed over time and during windy episodes since the valvate openings are small. Growing on exposed sites, the scapes often protrude through a thin snow cover and are adapted to efficient long-distance dispersal also on the snow. A study from Svalbard by Alsos et al. (2007) showed that plant colonisation to these High Arctic islands had predominantly occurred across the sea-ice from the west or the east. This probably explains the broad distributions of only a few High-Arctic taxa of *Oreomecon*, such as O.dahlianasubsp.polaris and *O.cornwallisensis*, over the northernmost land areas from Canada to Arctic Europe.

The clade combining *Meconopsis* and *Oreomecon* evolved as a response to the dramatic uplift of the Himalayan and the neighbouring Hengduan Mountain ranges and the eastern Tibetan Plateau at 25–20 Ma (Xie et al. 2014). *Meconopsis* adapted to monsoonal climates initiated at ca. 20 Ma, although the major clades within the genus had a rapid early divergence involving polyploidy. Some also invaded drier plateau habitats to the north (Xie et al. 2014) and sectionAculeatae is concentrated in the drier areas and is centred in northernmost Pakistan and adjacent India (Grey-Wilson 2014). Wen et al. (2014) showed how the uplift of the Qinghai-Tibetan Plateau has worked as a driver of evolution, instigating spectacular radiations and species diversification also in numerous other genera, for example, *Pedicularis* L., *Primula* L., *Rhodiola* L., *Rhododendron* L., *Saxifraga* L. and *Saussurea* DC.

At about 20 Ma, the global climate was about 4 °C warmer than at present and alpine climates probably occurred only at altitudes between 5,000 and 6,500 m. However, the global climate cooled at ca. 12 Ma (Herbert et al. 2016), facilitating the evolution of *Oreomecon* (Xie et al. 2014). *Papaver* and *Meconopsis* are classified within systems of several sections, most well-defined in morphology and genetics. This is not the case with *Oreomecon*, which shares a median crown age of 16.6 Ma with *Meconopsis*, according to Xie et al. (2014). According to Kadereit et al. (2011), the diversification of extant species of *Oreomecon* started much later, at ca. 5 Ma. This may indicate that early clade representatives became extinct, whereas those surviving were well adapted to the extreme cooling during the Pleistocene.

The distribution maps of *Meconopsis* and *Oreomecon* in Fig. 2 show a rather narrowly overlapping zone between them. Concerning *Oreomecon*, Fig. 2 shows that it has its concentration of species in Asia, with 24 species in central parts, ten taxa in the Asian Far East and 20 taxa in Arctic Asia. It extends into Arctic Alaska and Yukon and adjacent Cordilleras with nine taxa, with another five species further to the south in the North American Cordillera. However, in the vast area ranging from central Arctic Canada throughout Greenland, including the entire Arctic Europe, only six species and subspecies occur. The Nordic *O.radicata* complex is apparently the most studied part of the genus, although different classification alternatives have been proposed. Here, we conclude on eight subspecies and four varieties, based on morphometric studies, which are pending future molecular studies. In Central Europe, where the taxonomy of this genus is also controversial, we propose to accept three species and recognise the remaining entities preliminarily at the subspecies level.

Rändel (1974) argued that *Oreomecon* had its evolutionary centre in Central Asia, as indicated by the concentration of diploids there. Later migrations northwards and longitudinally involved a high degree of polyploidy and hybridisation. Exceptions are the American *O.walpolei* and *O.pygmaea* and the European *O.alpina* s.lat. as they are diploids outside the postulated area of origin. Kadereit (1988a) found this explanation hypothetical, although the much later phylogenetic conclusions that *Oreomecon* and *Meconopsis* are sister genera support the views of Rändel (1974).

Applying molecular analyses, Solstad et al. (2009) confirmed that *O.radicata* is, indeed restricted to the previously heavily glaciated Nordic area. Early Scandinavian authors like Nordhagen (1932) used the fragmented distribution pattern of *Oreomecon* taxa as evidence of glacial survival in the Nordic countries. In contrast, later authors (i.e. Nordal (1987)) argued in favour of post-glacial immigration. Westergaard et al. (2018), however, presented evidence for Weichselian survival in the case of *Carexscirpoidea* Michx., a species widely distributed in northern North America, but absent from Europe, except for a few sites in north Norway. Here, it grows in low-alpine habitats, which starkly contrasts *Oreomecon* at higher altitudes in the high-alpine zone. Hence, *Oreomecon* species therefore appear to be better adapted to glacial survival.

*Oreomeconradicata* probably originated from neighbouring taxa within a continuous adjacent distribution and the species forms an evolutionary group with *O. lapponica* subsp. *jugorica* as its closest taxon (Solstad et al. 2009). The relationship between *O.radicata* and the *O.dahliana* group is more distant. This group is represented in the amphi-Beringian area by a still undescribed species referred to as “Papaveraff.dahlianum” by Solstad et al. (2009). Their conclusion from molecular studies is that these species do not have connections to any known diploid ancestral species.

Our recombination of O.lapponicasubsp.laestadiana is based on comparative cultivations in the rock garden exposed to the cool climate of Tromsø. This taxon was evaluated by Nordhagen (1939) to be the most exclusively high-alpine representative of the genus in Norway and very difficult to cultivate in the lowlands of south Norway, as opposed to *O.radicata*.

*Oreomeconlapponica* specimens are highly modified by their contrasting habitats. This is evident from herbarium specimens at TROM, partly collected by the first author from High Arctic Canada (subsp.occidentalis) and east Greenland (probably subsp.lapponica). Those from higher latitudes and altitudes are compact, in contrast to the very elongated specimens, for example, from 68°N in west Greenland (subsp.occidentalis) and only 60°N in the Ural Mts. (ssp.jugorica). The same contrast is shown for herbarium specimens of O.lapponicasubsp.lapponica from lowland riverbanks in north Norway vs. high-alpine populations of O.lapponicasubsp.laestadiana. Population samples can be more reliably identified and compared with other taxa by comparative cultivation, an approach strongly recommended for *Papaver* (Knaben 1959b; 1979) and other vascular plants (e.g. Emig and Kadereit (1993)).

The Central European complex of *Oreomecon* most likely originated from an extreme long-distance dispersal event. According to Popov (1937), “*P.nivale* is readily distinguished from all other Soviet representatives of the sectionScapiflora and is related to the European *P.alpinum*”. This was confirmed by Rändel (1974), who only found differences in capsule hair colour. The *Oreomeconalpina* complex is genetically not closely related to any analysed Asian species (Solstad et al. 2009) and is also confirmed by our summary of existing ITS-based phylogenies (Suppl. material 1). No recent studies have addressed the relationship between *O.alpina* and *O.nivalis*; the latter is a species belonging to the *O.pulvinata* complex, according to Petrovsky (1999). The possibility of a Weichselian long-distance dispersal event cannot be excluded; compare the case of the local endemic Oxytropisdeflexasubsp.norvegica Nordh., known from populations in two screes in continental northern Norway. The ancestor of Oxytropisdeflexasubsp.norvegica migrated postglacially across a then vast, open and gravelly periglacial landscape of present-day Russia, then becoming extinct there, leaving a large, present-day distribution gap to the nearest occurrences of Oxytropisdeflexasubsp.deflexa (Pall.) DC. in Kazakhstan (Elvebakk 1984; POWO 2023).

However, *Oreomeconnivalis* is tetraploid (Solstad et al. 2009) and the closest relative of *O.alpina* has still not been identified (Kadereit et al. 2008). The *O.alpina* complex has a distribution pattern strikingly similar to that of Primulasect.Auricula Duby (Zhang et al. 2004; Aymerich et al. 2014). The latter group comprises 26 species, which are reasonably well differentiated morphologically and genetically (Zhang et al. 2004). This suggests a considerably older origin than that of the *O.alpina* complex. Zhang et al. (2004) estimated the diversification of the former from an Asian ancestor to have taken place at 3.59 Ma.

The treatment followed here, with the formal recognition of both subspecies and varieties of *O.radicata* in northern Europe, parallels the treatment of the *Androsacevitaliana* (L.) Lapeyr. complex, previously the genus *Vitaliana* Sesl., by Dixon et al. (2016). Except for being absent from east European mountains, this group has a similar distribution pattern as the *Oreomeconalpina* complex. Dixon et al. (2016) accepted ten taxa, all with allopatric distributions. Only three of the six subspecies were considered to be morphologically distinct. The Pyrenean subspecies appears to have evolved through gene flow from entities further south in the Iberian Peninsula, where four geographically distinct varieties within one subspecies are maintained despite unconfirmed morphological and genetic characteristics.

Our chosen alternative of accepting three western taxa in addition to a complex of *O.alpina* further east was proposed already by Ascherson (1869). Claiming to follow the then-new Darwinian theory, he put forward the hypothesis that a northern Asian member of the circumpolar *P.nudicaule* s.lat. complex had dispersed to Central Europe. Here, it first evolved at the species level as the taxon now referred to as *P.aurantiacum*, with a distinct morphology, as cited from its type locality at Mt. Ventoux. Further evolution produced another distinct species in western Europe, referred to as *P.suaveolens*, which in the east Pyrenees diverged into another taxon, P.suaveolensvar.endressii Asch., distinguished by its dissected leaves. In the central and eastern parts of the Alps, various distinct forms with intermediates referred to as the *P.alpinum* complex had not yet reached differentiation corresponding to species level.

Burbrink et al. (2022) proposed to abandon the use of the subspecies category in taxonomy, as the major criterion of reproductive isolation used in their field of vertebrate zoology is often difficult to adapt. Phylogeny should, therefore, decide whether populations represent a species or whether to be left untreated by formal taxonomy. In botany, many taxa have not been studied or have been insufficiently studied by phylogeny and Molinari (2023) proposed morphology as a decisive additional criterion, whereas Wood et al. (2015) and POWO (2023) emphasised vicariant evolution. These criteria have been used to define the numerous subspecies accepted by the present study. This is particularly the case for *Oreomecon* subspecies from areas with a dramatic history of glaciation, where time has been insufficient for a taxon to develop into a fully-defined species.

Solstad et al. (2009) concluded that “*Papaveralpinum* seems to have been isolated in Europe for a fairly long time”. This is supported by the many geographically defined lineages which are genetically distinct. Many such named lineages are now threatened and Red-listed, primarily because of the effects of global warming and conservation issues are becoming imminent in many countries. Further studies need to address the patterns of genetic vs. morphological variation, including improved genetic markers and also comparative cultivation. However, we believe that maintaining a traditional, although imperfect, concept of several *Oreomeconalpina* subspecies is to be preferred over the alternative of leaving them unnamed.

## ﻿Conclusions

We propose that the evolution of the Old-World group of Papavereae is best reflected within a system of eight genera. The sister genera *Meconopsis* and *Oreomecon* are the two most speciose genera. Their evolution was instigated by the dramatic uplift of the Qinghai-Tibetan Plateau, where *Meconopsis* is concentrated in the southern monsoon-influenced parts. *Oreomecon* probably evolved in the northern rain-shadow area and eight of the Central Asian species treated by Peschkova (1994) were characterised by being from steppe or steppe-like habitats. From Central Asia, the genus radiated to high mountains and Arctic areas on a circumpolar scale (Rändel 1974), shown in Fig. 2 as a map and with numbers of accepted taxa for eight geographical areas provided in Table 1. The genus *Oreomecon* was recently described as a genus replacing Papaversect.Meconella (Banfi et al. 2022) and more recent recombinations have resulted in 82 species names proposed for the new genus. Here, we argue in favour of accepting subspecies status for many taxa according to traditional treatments by *Papaver* experts. *Oreomecon*, which comprises many incompletely understood taxa, is reduced to 68 species here, with 38 *Oreomecon* names newly introduced, most of them as recombinations, whereas 21 existing *Oreomecon* names are reduced to synonymy.

The sections *Horrida* and *Californica* of *Papaver* are here treated as the genera *Afropapaver* and *Stylomecon*, respectively. We accept the generic status *Parameconopsis* for the species now most commonly treated as *Papavercambricum* and define *Papaver* s.str. as the sister group of *Parameconopsis* after the latest major divergence in the group. The distributions of the genera are illustrated here (Figs 2, 4), with indications of the number of accepted species and subspecies based on key reference studies supplied by a review of subsequent literature.

## ﻿Additional information

### Author ORCIDs

Arve Elvebakk https://orcid.org/0000-0002-7682-3797

Jarle W. Bjerke https://orcid.org/0000-0003-2721-1492

### Data availability

All of the data that support the findings of this study are available in the main text or Supplementary Information.

## Supplementary materials

Supplementary material 1 A phylogram including all ITS sequences of *Oreomecon* available at GenBank https://doi.org/10.3897/phytokeys.248.121011.suppl1 Data type docx https://binary.pensoft.net/file/1163372This dataset is made available under the Open Database License (http://opendatacommons.org/licenses/odbl/1.0/). The Open Database License (ODbL) is a license agreement intended to allow users to freely share, modify, and use this Dataset while maintaining this same freedom for others, provided that the original source and author(s) are credited.Arve Elvebakk, Jarle W. Bjerke
