# Supplementary material for: ﻿Papaver recircumscribed: A review of neighbouring Papaveraceae genera, including Afropapaver nom. et stat. nov. and Oreomecon, a large, Arctic-Alpine genus
Source: PhytoKeys. 2024 Oct 29;248:105–88. doi: 10.3897/phytokeys.248.121011 (PMC11538858; doi:10.3897/phytokeys.248.121011)
Supplement: Supplementary material 1 — A phylogram including all ITS sequences of Oreomecon available at GenBank [file phytokeys-248-105_article-121011__-s001.docx]

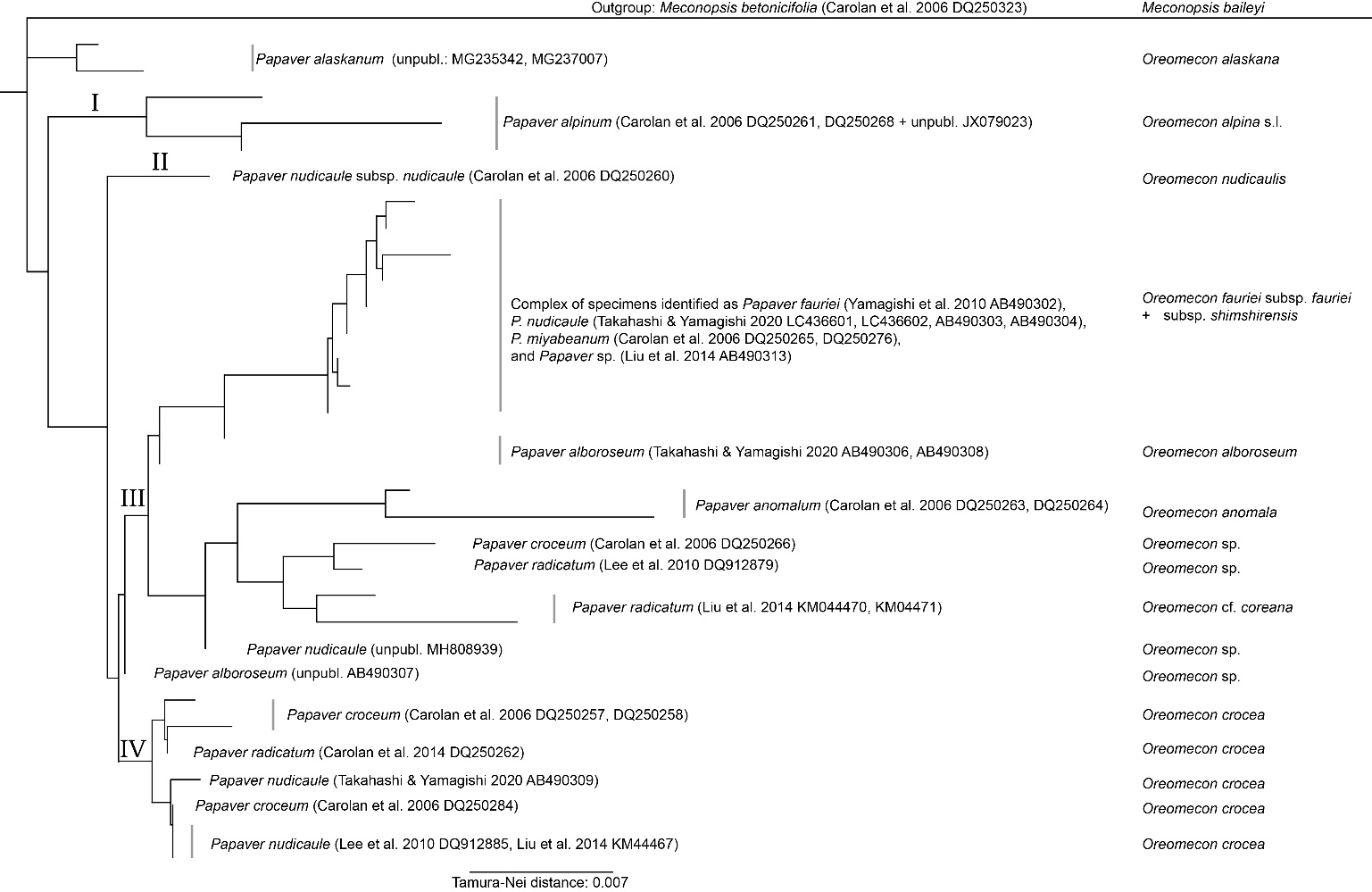


Fig.S1. A phylogram including all ITS sequences of *Oreomecon* available at GenBank. The original names and GenBank accession numbers are indicated in the phylogram, whereas the present interpretations as taxa within *Oreomecon* are indicated to the right. Tamura-Nei distance is shown on a scale-bar and four discussed clades are marked as I‒IV.
